# CYP46A1 activation by low-dose efavirenz uncovers the link between brain cholesterol metabolism, energetics, and vasculature

**DOI:** 10.1016/j.biopha.2026.119470

**Published:** 2026-04-30

**Authors:** Natalia Mast, Ilya Bederman, Nicole El-Darzi, Irina A. Pikuleva

**Affiliations:** a Department of Ophthalmology and Visual Science, Case Western Reserve University, Cleveland, OH 44106, USA; b Department of Genetics and Genome Sciences, Case Western Reserve University, Cleveland, OH 44106, USA

**Keywords:** Phosphatidylcholine, Sphingomyelin, Acetyl-CoA, Myelination, Metabolic flexibility, Vascular deficits

## Abstract

CYP46A1 converts cholesterol to 24-hydroxycholesterol, the principal mechanism for brain cholesterol removal and turnover. CYP46A1 can be allosterically activated with low-dose anti-HIV drug efavirenz and mitigate the manifestations of various neurologic diseases in mouse models and Niemann-Pick type C disease in humans. Yet the underlying reasons for such a broad range of efavirenz therapeutic effects are currently unknown. Here 5XFAD mice, a model of Alzheimer’s disease, were treated with low-dose efavirenz, and assessed for changes in their brain proteome, acetylproteome, and metabolome. Sex-independent increases in brain levels of phosphatidylcholines, sphingomyelins, and certain amino acids were documented, and various functional enrichments were identified. The most notable related to brain energy production, vascularization, and prevention of glutamatergic overactivation. Unexpectedly, these and many other enrichments were mediated by different proteins in female and male 5XFAD mice. Efavirenz treatment of 5XFAD mice was repeated, and energy-related compounds were quantified in the brain after *in vivo* isotopic labeling. Cerebral vasculature was assessed as well. We found increased glycolysis branching, carbon flux through the tricarboxylic acid cycle, and use of alternative energy sources (fatty acids, ketone bodies, and amino acids). Sex-independent improvements in brain vascularization and integrity of the blood-brain barrier were also documented. Collectively, our data suggested that CYP46A1 activation by efavirenz increases brain metabolic flexibility and thereby brain energetics. This enables the increase in production of the building blocks for cellular and tissue repair and rescue of brain pathology, thus explaining the therapeutic benefits for the broad spectrum of neurologic disorders.

## 1. Introduction

Cholesterol is abundant in the brain and essential for many brain functions [1]. The brain cannot exchange cholesterol with the systemic circulation because of the blood-brain barrier. Therefore, local biosynthesis is the major source of cholesterol for the brain, and metabolism to 24-hydroxycholesterol (24HC) is the major elimination route [1–3]. Both processes are tightly coupled to maintain homeostasis and cholesterol turnover [2,3].

Cholesterol 24-hydroxylation is a CNS-specific reaction normally occurring in neurons and catalyzed by CYP46A1 [4]. We extensively studied CYP46A1 and discovered that its activity can be modulated by an anti-HIV drug efavirenz (EFV). Indeed, *in vitr*o and when at low concentrations, EFV interacted with a specific (allosteric) site on the CYP46A1 cytosolic surface and enhanced enzymatic activity. Yet, at high concentrations, EFV binding was to the catalytic site inside the protein molecule inhibiting cholesterol 24-hydroxylation [5–7]. Similar effects were observed upon EFV administration to mice, with low EFV doses activating CYP46A1 and high doses inhibiting the enzyme [5]. Notably, when given to 5XFAD mice, a model of Alzheimer’s disease (AD) [8], low-dose EFV improved, among other effects, performance in memory tasks [9,10]. We then crossed 5XFAD and *Cyp46a1*^*−/−*^ mice and used 5XFAD*Cyp46a1*^*−/−*^ animals to confirm that EFV effects were mostly due to CYP46A1 activation [11].

Our studies prompted other laboratories to test EFV on mouse models of Niemann-Pick type C disease, prion diseases, depression, epileptic seizers, and glioblastoma and document the mitigation of many of the diseases’ manifestations [12–17]. Similarly, therapeutic benefits were observed after cerebral *CYP46A1* gene therapy in mouse models of AD, Huntington’s disease, amyotrophic lateral sclerosis, and spinocerebellar ataxia type 3 [18–22]. Increased CYP46A1 activity in various mouse models led to the effects, which were specific to each disease; hence we called them conditional. In addition. there were effects, which were similar among the tested models; therefore, we called them unconditional [23,24]. The latter were on common hallmarks of neurodegenerative diseases and included reductions in misfolded protein accumulation, improvements in cognition, motor function, and autophagy [24,25].

We extended our investigation to humans by conducting a clinical trial of EFV in subjects with early AD. We identified the EFV doses activating CYP46A1 in human brains and demonstrated their safety in the geriatric population [26]. These doses were then applied in a clinical trial for Niemann-Pick type C disease, a cholesterol transport disorder, which like AD leads to progressive cognitive and motor declines [27]. EFV treatment stopped cognitive deteriorations in all trial participants, and some even had slight neuropsychological and neurological improvements [28]. Because of its success, the trial was extended for another year. Now, low-dose EFV is being tested for Creutzfeldt-Jakob (prion) disease (NCT07482085).

**Fig. 1.**
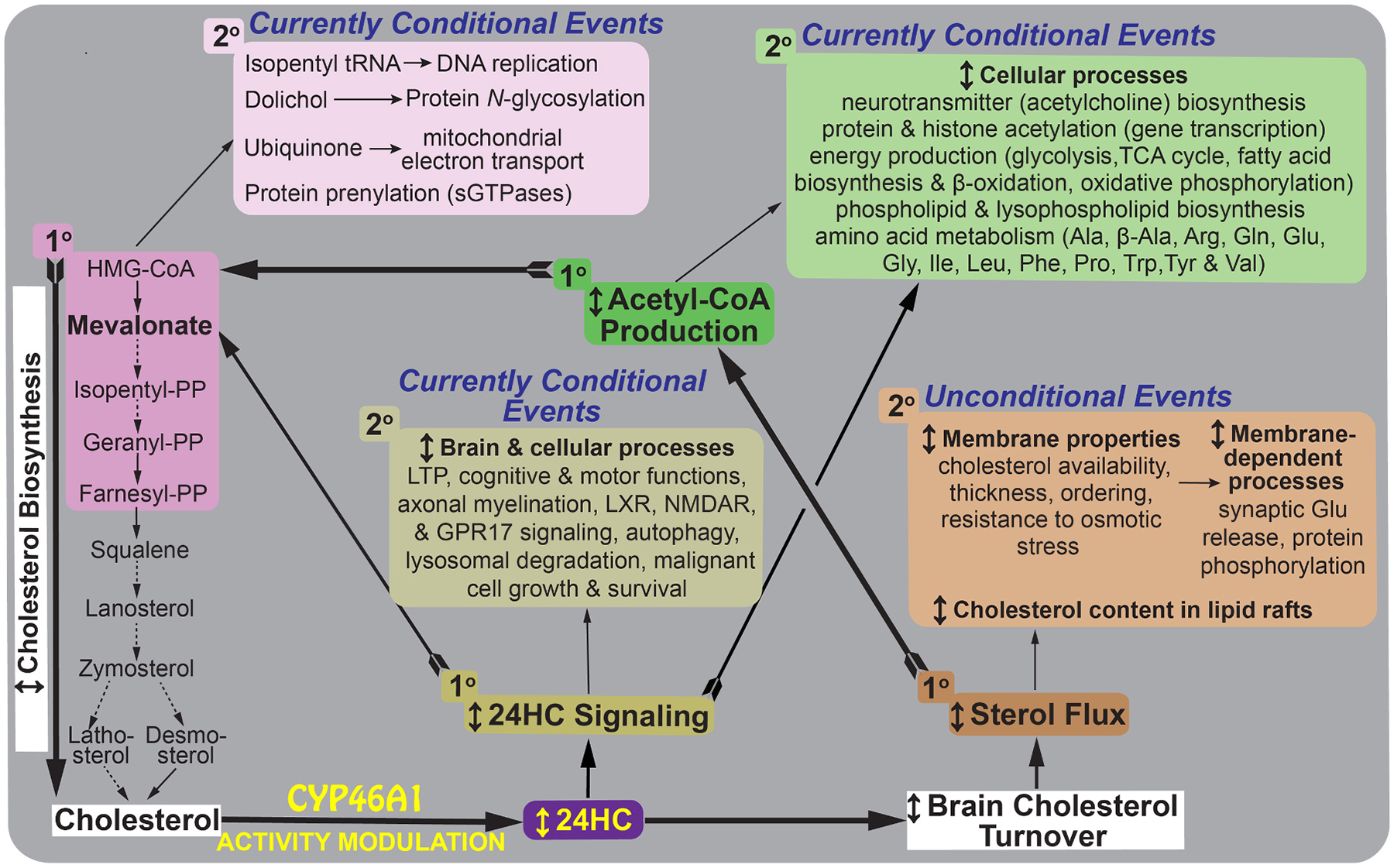
The chain reaction hypothesis. Schematic representation of the chain reaction hypothesis explaining the multiplicity of CYP46A1 activity effects in the brain. Brown, green, magenta, and olive colors indicate different primary (1°) CYP46A1 activity effects with the corresponding secondary (2°) effects being in a lighter color shade. Thick feathered black arrows start from the 1° effects, and thin black arrows link the 1° and 2° events; the latter being labeled as unconditional or currently conditional. Dashed arrows indicate multiple steps, and ↕ indicates modulation (increase or decrease). We first found that increased and decreased sterol fluxes had opposite effects (no sex differences) on physico-chemical membrane properties, synaptosomal glutamate release, and inhibition of membrane-dependent protein kinases and protein phosphatases [32]. The latter explained altered protein phosphorylation in the brain of EFV-treated *vs* control 5XFAD mice and *Cyp46a1*^*−/−*^*vs* WT mice [29,186]. Next, we linked sterol flux to the acetyl-CoA production based on changes in synaptosomal proteome in EFV-treated *vs* control 5XFAD male mice [30] as well as the measurements of the acetyl-CoA pools in brain homogenates and mitochondria of EFV-treated *vs* control 5XFAD mice of both sexes [39,187] and male *Cyp46a1*^*−/−*^ *vs* WT mice [30,31]. Both acetyl-CoA pools were increased and decreased upon increase and decrease, respectively, of sterol fluxes and affected the levels of acetylcholine [39,187], which are decreased in AD [188]. Effects on other processes were suggested as well based on functional enrichments in the mutiomics studies of EFV-treated *vs* control 5XFAD male mice [31]. Then we linked acetyl-CoA production to the mevalonate portion of the cholesterol biosynthesis pathway (the first eight steps) as a total of three acetyl-CoA molecules are necessary for mevalonate synthesis, the rate-limiting and irreversible step in cholesterol biosynthesis [189]. Despite cholesterol supply from astrocytes [98], mevalonate synthesis is active in brain neurons to ensure the production of non-sterol compounds [isopentyl tRNA, dolichol, ubiquinone, geranyl pyrophosphate (PP) and farnesyl-PP] and to couple mevalonate synthesis to cholesterol elimination by CYP46A1 [3,4,190]. The non-sterol compounds in the mevalonate pathway are essential for many brain processes [23,44], and several of these processes have already been shown to depend on CYP46A1 activity: long-term potentiation (LTP), protein prenylation, and sGTPase signaling [31,44,45]. Finally, 24HC can bind to INSIG1 (insulin-induced gene 1), LXRs (liver X receptors), NMDARs (N-methyl-D-aspartate receptors), and GPR17 (G protein-coupled receptor 17) and affect cellular cholesterol biosynthesis, efflux, and transport [191], fatty acid biosynthesis [192], cognitive and motor functions [13,14,20,22,33,34,36–38,192,193], myelination [36,194–198], autophagy [21,22], lysosomal processing [13,22,199,200], malignant cell growth [14], and cell survival [14]. We suggest that signaling *via* 24HC could serve as a shunt to both 1° and 2° CYP46A1 activity effects. Thus, altered CYP46A1 expression or activity in the brain can trigger a chain reaction, i.e., a sequence of 1° events, which in turn elicit 2° effects and collectively underlie the multiplicity of the brain CYP46A1 effects. HMG-CoA, 3-hydroxy-3-methylglutaryl-CoA; PP, pyrophosphate.

To explain the broad range of brain disorders benefiting from EFV treatment in mouse models as well as the multiplicity of CYP46A1 brain effects, we suggested the “chain reaction” (Fig. 1). We reasoned that there are primary CYP46A1 activity effects, mostly unconditional, which elicit in turn numerous secondary effects, either conditional or unconditional [24,29–32]. Since 24HC is not only a cholesterol metabolite but also a cell- and tissue-specific activator of various receptors [14, 33–38], we included 24HC signaling as a conditional primary effect (Fig. 1). Plus we identified the primary effects, which were unconditional and currently include changes in sterol flux through the plasma membranes, acetyl-CoA production, and mevalonate synthesis [30,32,39] (Fig. 1).

Of the secondary CYP46A1 effects, 24HC signaling was linked to glutamatergic neurotransmission [40,41], whereas mevalonate production to learning and function of small GTPases [31,42–45]. Sterol flux was shown to affect physico-chemical properties of plasma membranes, synaptic Glu release, and protein phosphorylation [29,32]. Acetyl-CoA production affected acetylcholine and phospholipid biosyntheses, protein and histone acetylation, energy generation, and amino acid metabolism [29–32,42]. We also established that brain effects secondary to altered sterol flux were unconditional as they were observed in mice of both sexes and different genotypes [29,32]. Conversely, the effects secondary to acetyl-CoA and mevalonate production are currently conditional as they were observed so far in our multiomics studies conducted only on mice of one sex (males) and genotype (5XFAD) [31]. Therefore, in the present work, we carried out a multiomics study on EFV-treated *vs* control 5XFAD female mice and then used both sexes for the follow up evaluation of the EFV treatment effects on brain energy production and status of cerebral vasculature. We established the biochemical basis for the multiplicity of EFV brain effects and identified novel brain processes that could benefit from CYP46A1 activation.

## 2. Materials and methods

### 2.1. Animals

All animals were from the Jackson Laboratory (Bar Harbor, ME, USA) - B6SJL (#100012) or wild type (WT) mice and 5XFAD mice (5XFAD^*Tg/0*^, #34840) on the B6SJL background. The latter were hemizygous for the mutant K670N/M671L/ I716V/V717I human amyloid precursor protein 695 and mutant M146L/L286V human presenilin 1 [8]. Female B6SJL mice were crossed with male 5XFAD mice and only the F1 generation of hemizygous animals was used. Before breeding, the *Pde6b*^*rd1*^ mutation that is present in B6SJL mice and leads to early onset severe retinal degeneration and blindness [46] was bred out from our colony. Control and treated groups were comprised of littermates, which were randomly divided and matched by sex. The pool of all available animals was used, and the sample size was based on previous experience. The investigators were not blinded with respect to treatment, as they were involved in both animal treatment and subsequent tissue isolation. Mice received EFV (the *S*-isomer, E425000, Toronto Research Chemical, Toronto, ON, Canada) in drinking water containing 0.0004% Tween 80 at a 0.1 mg/kg body weight/day dose from 3 to 9 months of age as described [10]. Control animals received aqueous 0.0004% Tween 80. Mice were maintained in a temperature- and humidity-controlled environment with 12 h light/12 h dark cycle in cages with water and food ad libitum. To minimize circadian rhythm effects, experimental manipulations and sample collection were always performed at the same time and within a short time window, occurring in a randomized order amongst experimental groups. All animal experiments were approved by the Case Western Reserve University’s Institutional Animal Care and Use Committee and conformed to recommendations by the American Veterinary Association Panel on Euthanasia.

### 2.2. Brain isolation

This was as described [31]. Briefly, mice were fasted overnight and euthanized the next morning by a bolus intraperitoneal injection of ketamine (100 mg/kg) and xylazine (10 mg/kg). Mouse brains were isolated and rinsed in cold phosphate buffered saline. The brains were blotted dry and dissected to remove the olfactory bulb and brain stem beyond the cerebellum. The remaining part was cut along the midline to obtain two hemispheres, which were flash-frozen in liquid nitrogen.

### 2.3. Brain proteomics and acetylproteomics

Samples were from female mice (n = 5/group) as a similar study was previously conducted on male mice [31]. Right brain hemispheres were used for proteomics and acetylproteomics analyses, and left brain hemispheres were used for metabolomics analysis. Brain samples were processed as described previously for male mice [31] and sent for omics quantifications to Creative Proteomics (Shirley, NY, USA), a proteomics mass spectrometry company. Relative peptide abundance was assessed by the label-free approach as described [31], except 4D proteomics was used; a fourth dimension (ion mobility) was added to peptide retention time, mass-to-charge ratio, and signal intensity to improve the detection of low-abundant peptides.

FragPipe software (version 21.1) [47] was used for qualitative and quantitative protein analyses. Proteins (acetylated peptides) with a fold change of ≤ 0.83 and ≥ 1.2 or log_2_ fold change of ≤ −0.08 and ≥ 0.08 (an arbitrary cut off), and *P* values of ≤ 0.05 were considered as differentially expressed (DEPs) or differentially acetylated (DAPs).

### 2.4. Untargeted brain metabolomics

Sample preparation and metabolomic analyses were performed as described [31] in both positive (+ESI) and negative (−ESI) electrospray ionization modes. Raw data were processed with the Compound Discoverer 3.1 software (ThermoFisher Scientific, Waltham, MA, USA) as described [31] and matched with the mzCloud and mzVault databases (50). Statistical analyses were separate for the positive (+ESI) and negative (−ESI) MS data after each metabolite abundance was expressed as a peak area and normalized by the total peak area of each sample followed by a fold-change calculation. Metabolites with a fold change of ≤ 0.83 and ≥ 1.2 or log_2_ fold change of ≤ −0.08 and ≥ 0.08 (an arbitrary cut off), and *P* values of ≤ 0.05 were considered as differentially abundant (DAMs). A two-tailed, unpaired Student’s t test was used to calculate the *P* value.

### 2.5. Multiomics data analyses

STRING [48] and PANTHER [49] were used to analyze DEPs and DAPs, and MetaboAnalyst [50] to conduct integrated analysis of DEPs, DAPs, and DAMs based on the KEGG (Kyoto Encyclopedia of Genes and Genomes) pathway database and pathway categorization [51]. Only enrichments with the *P* values of ≤ 0.05 were used. Heatmaps were generated by the GraphPad Prism software. Protein annotation was according to UniProt [52].

### 2.6. Targeted quantifications of brain glucose metabolites and other compounds

This was carried out as described [53] on samples from both sexes (n = 5/sex/group). Briefly, male and female 5XFAD mice were fasted overnight in bedding-free clean cages, and the next morning were injected intraperitoneally with 40 mg (0.2 ml) of [U-^13^C_6_]glucose (Cambridge Isotope Laboratories Inc., CLM-1396-PK) in saline. Thirty minutes post-injection animals were sacrificed, and their plasma and brains were isolated. Processing of the individual plasma samples (20 μl) was as described [53] after the addition of 0.5 ml of acetone and internal standard (20 μg of D-[1,2-^13^C_2_]-glucose from Cambridge Isotope Laboratories Inc., Tewksbury, MA, USA, CLM-504). Plasma samples were vortexed and centrifuged at room temperature, 4000 *g* for 5 min. The supernatants obtained were transferred to the gas chromatography-mass spectrometry (GC-MS) vials and evaporated to dryness. Acetic anhydride (0.15 ml) in pyridine (2:1, vol/vol) was added to the vials to obtain pentaacetate glucose derivative by reacting at 80°C for 30 min. Solutions were evaporated to dryness and reconstituted in 0.1 ml of ethyl acetate. Samples were transferred to the GC-MS inserts, crimped, and 1 μl was used for GC-MS quantifications. The plasma glucose *m/z* values of 200 (M0) and 205 (M6) were monitored, and plasma glucose enrichment was calculated as a ratio of (M6)/Σ(M0 +M6). The glucose concentration was calculated based on a calibration curve (y = 5.242 +0.6733, R^2^=0.9937) and a ratio of Σ(M0-M6) to M2 of the D-[1,2-^13^C_2_]-glucose internal standard.

For the quantifications in the brain, one brain hemisphere (left) was homogenized in 5 ml of chloroform-methanol (2:1, vol/vol) followed by centrifugation at 4°C, 4000 *g* for 15 min. The pellets were rehomogenized in 5 ml of chloroform-methanol (2:1, vol/vol) and spun down again. The supernatants from the two homogenizations were combined, mixed with 1 ml of water, vortexed, and subjected to centrifugation at room temperature, 4000 g for 15 min. The upper aqueous layers were taken and evaporated to dryness. Extracts were then dissolved in 0.5 ml of 100% methanol, transferred into separate vials for the measurements of brain glucose (0.1 ml, spiked with 20 μg of D-[1,2-^13^C]-glucose) and brain glucose metabolites [0.4 ml, spiked with 2 μg of heptadecanoic acid (MilliporeSigma, #H3500)], and evaporated to dryness. For the glucose measurements, dried samples were mixed with acetic anhydride (150 μl) in pyridine (2:1, vol/vol) and kept at 80°C for 30 min. Solutions were evaporated to dryness, dissolved in 100 μl of ethyl acetate, and analyzed (1 μl) by GC-MS. For the brain glucose metabolite quantifications, dried samples were mixed with 0.1 ml of 2% methoxyamine in pyridine (ThermoFisher Scientific, TS-45950) and kept at 70 °C for 2 hrs. Samples were then evaporated, derivatized at 70°C for 30 min in 70 μl of tert-butylbis(dimethylsilyl) trifluoroacetamide plus 10% trimethylchlorosilane (Regisil, Regis Technologies, #1–270111–200), and analyzed (1 μl) by GC-MS.

The lower organic phase after the brain chloroform-methanol extraction was evaporated to dryness, reconstituted in 1 ml of hexane, and used for free (0.3 ml) and total (0.3 ml) fatty acids quantification after spiking with 2 μg of heptadecanoic acid. Prior to further processing, samples for the total fatty acid quantifications were hydrolyzed at room temperature overnight in 1 ml of 70% ethanolic KOH and then acidified with 0.15 ml of 12 N HCl plus 1 ml of water. Samples were extracted with 6 ml of hexane, centrifuged at room temperature, 4000 *g* for 10 min, and the lower organic phase was isolated and evaporated to dryness. Samples for free and total fatty acid determination were methylated at 40 °C overnight in 2 ml of 5% (vol/vol) methanolic HCl. The next day, samples were heated at 60 °C for 30 min and mixed with 1 ml of water plus 4 ml of hexane. Samples were vortexed and centrifuged at room temperature, 4000 *g* for 10 min. The supernatants obtained were evaporated to dryness, reconstituted in 120 μl of hexane and subjected (1 μl) to GC-MS.

All GC-MS analyses were run on an Agilent 5973N-MSD mass spectrometer (Agilen Technologies, Santa Clara, CA, USA) equipped with an Agilent 6890 Gas Chromatograph. A HP-5MS capillary column (60 m × 0.25 mm × 0.25 μm, Agilent Technologies) was used for all runs with a helium flow of 1.5 ml/min. Samples were analyzed in selected ion monitoring mode using electron impact ionization; the ion dwell time was set to 10 msec. The steady state concentrations were determined as a ratio of metabolite abundance to the standard abundance. ^13^C-labeling of glucose metabolites was expressed as MPE (molar percent enrichment, ratio of abundances of ^13^C-labeled metabolites/sum of (^13^C-labeled metabolites and C-unlabeled metabolites) × 100. The fractional synthesis rate was calculated based on the precursor-product relationship using molar percent enrichment of [^13^C]glucose as the precursor and molar percent enrichment of [^13^C]metabolite as the product. The absolute rates of glucose metabolite synthesis was determined as a product of relative metabolite concentration and fractional synthesis rate.

### 2.7. Profiling of brain sterols

Cholesterol, 24HC, lathosterol, and desmosterol were quantified by isotope dilution gas chromatography-mass spectrometry as described [54] using deuterated sterol analogs as internal standards (n = 5/sex/group).

### 2.8. Acetyl-CoA and acetylcholine quantifications

Acetyl-CoA was measured in brain homogenates and mitochondria as described [30] using the acetyl-CoA assay kit (Sigma-Aldrich, St. Louis, MO, USA, #MAK039) (n = 5/sex/group). Total and free choline were quantified in brain homogenates as described [39], also by a kit (Sigma-Aldrich, #MAK056) [39], with a difference between the two compounds representing the acetylcholine levels.

### 2.9. Brain vasculature

For ultrasound imaging or angio 3D tomography, mice (3 females/group) were first anesthetized with 2% isoflurane (Aspen Veterinary Resources, Liberty, MO, USA, 46066–115–04) followed by hair removal from the cranial region with a hair removal cream. Animals were then secured in a stereotaxic frame and maintained under isoflurane anesthesia during subsequent imaging by an ultrafast ultrasound scanner (Iconeus One, 256 channels; Iconeus, Paris, France) equipped with a 15-MHz transducer (IcoPrime-Lite, 128 elements; Iconeus, Paris, France). The transducer center frequency was 15 MHz and spatial resolution was 100 × 100 μm^2^. The ultrasound probe was mounted on a motorized linear stage with a 10-mm travel range and was automatically rotated by 10° clockwise after each sweep. The integrated IcoStudio software (Iconeus, Paris, France) was used for data acquisition and image generation.

Evans blue (Sigma-Aldrich, St. Louis, MO, USA, E2129) and fluorescein isothiocyanate (FITC)-labeled dextran 2000 (Sigma-Aldrich, #52471) were used for brain fluorescence imaging after retro-orbital dye injections as described [55]. Briefly, mice (WT: 2 females and 3 males; control 5XFAD: 4 females and 5 males; EFV-treated: 5 males) were anesthetized with an intraperitoneal injection of 80 mg/kg ketamine plus 7 mg/kg xylazine. Evans blue was injected first (~0.1 ml of 2% solution in phosphate-buffered saline or 80 mg/kg body weight) followed by the FITC-dextran injection (~0.1 ml of 0.6% solution in phosphate-buffered saline or 24 mg/kg body weight) 18 min later [55]. Ten minutes post FITC-injection (or 28 min post Evans blue injection) mouse skulls were removed, and the brain surface was imaged for FITC fluorescence by a scanning laser ophthalmoscope (Spectralis HRA, Heidelberg Engineering, Franklin, MA, USA) as described previously for the retina [56]. The fluorescence quantifications were carried out by Metamorph (Molecular Devices, San Jose, CA, USA) after the brain midline and areas beyond the hemispheres were excluded due to variable blood accumulation. Brightness of the remaining brain hemispheres was adjusted to be the same across the images and normalized to their area. Cerebral vascular architecture was analyzed by the AngioTool software (Informer Technologies, Inc.) [57].

After i*n vivo* imaging by scanning laser ophthalmoscope, mouse brains were isolated and processed as described [55] by placing them in a tissue freezing medium (Leica OCT cryo compound, Leica Biosystems, Nussloch GmbH, Heidelberger, Germany) and flash-freezing in liquid nitrogen. Brain sections were cut coronally at a 20 μM thickness and imaged on a Zeiss AxioScan. Z1 (Carl Zeiss Research Microscopy Solutions, White Plains, NY, USA) equipped with the high-performance Hamamatsu ORCA Flash 4.0 v3 monochrome camera, 20x/0.8 Plan-Apochromat objective, and a Colibri 7 Illumination kit. The excitation and emission wavelengths for FITC-Dextran were 493 nm and 517 nm, respectively, and for Evan blue, 280 nm and 618 nm, respectively. Slides were also imaged for FITC and Evans blue fluorescence on an inverted microscope (DMI 6000 B; Leica Microsystems, Buffalo Grove, IL) using a Retiga EXi-Fast camera (QImaging, Surrey, BC, Canada).

### 2.10. Statistical analysis

All quantitative experiments were conducted with at least five independent biological replicates and included data (presented as mean ± standard deviation) from all animals with no exclusion of statistical outliers. Comparisons between two groups in the multiomics studies were by an unpaired two-tailed Student’s *t*-test, while multiple group comparisons in the targeted compound quantifications and vascular architecture analyses were by two-way and one ANOVA, respectively, with Tukey’s post hoc multiple comparison. When conducting these parametric tests, the Shapiro-Wilk test was used to assess normality. Data analysis and presentation was carried out by the GraphPad Prism 10.0 software (GraphPad, San Diego, CA, USA). Statistical significance was defined as *P ≤ 0.05, **P ≤ 0.01, and ***P ≤ 0.001.

## 3. Results

### 3.1. Brain proteomics

Approximately 5497 proteins were identified in the brain of female 5XFAD mice, and of them, 141 were affected by EFV treatment: 76 had decreased abundance and 65 had increased abundance (Supplementary Text 1, Supplementary Table 1). The identified DEPs were not the same as in male mice [31], and the intersex overlap was only at the level of several protein families: ARHG (the Rho guanine nucleotide exchange factors); FAM (family with sequence similarity); MRPL (the large ribosomal subunit proteins); NT5C (the cytosolic 5′-nucleotidases); SLC (small molecule transporters); SRSF (serine/arginine-rich splicing factors); and UBE2 (ubiquitin/ubiquitin-like-conjugating enzymes) (Supplementary Fig. 1).

### 3.2. Brain acetylproteomics

A total of 2519 acetylated proteins and 5286 acetylation sites were identified in the brain of female 5XFAD mice, and of them 157 proteins (173 acetylation sites) were affected by EFV treatment: 121 proteins (137 sites) had decreased abundance, and 36 proteins (36 sites) had increased abundance (Supplementary Text 1, Supplementary Table 2). Compared to male mice, EFV-treated female mice had a lower number of DAPs with increased acetylation (24% *vs* 97% [31], Supplementary Fig. 2), despite both sexes had similar absolute amounts and increases in the acetyl-CoA levels required for protein acetylation [39]. This intersex difference was also observed in 14 DAPs that were common in EFV-treated female and male 5XFAD mice (Supplementary Fig. 2): 11 DAPs (AASS, AP2M1, DLD, ETFA, GATD3, GLUD1, HIST1H4A, HADHA, IDH2, TCOF1, and YWHAZ) were at decreased abundance in female mice and increased abundance in male mice, two (CNP and NCL) were at increased abundance in both sexes, and one (GOT2) was at decreased abundance in both sexes. In addition, both sexes had DAPs from 9 common protein families: ACOT (acyl-coenzyme A thioesterases); ALDH (aldehyde dehydrogenases); ATP5 (complex V ATP synthases); HIST (histones); HSPA (chaperones); SEPTIN (filament-forming cytoskeletal GTPases); PRDX (peroxiredoxins); SLC (small molecule transporters); and SPT (spectrins) (Supplementary Fig. 2).

### 3.3. Brain metabolomics

A total of 825 metabolites were identified in the brain of female 5XFAD mice (Supplementary Table 3), not counting the compounds from targeted quantifications (Supplementary Table 3). Of them, 118 were affected by EFV treatment: 41 metabolites had decreased abundance, and 77 metabolites had increased abundance. Most of the DAMs in EFV-treated female mice were lipids and the same lipid classes as in male mice [31]: acylglycerols, N-acylethanolamines, carnitines, fatty acids (FA), glycerophospholipids, prostaglandins, sphingolipids [shin-gomyelins (SM) and ceramides], and sterols (Supplementary Fig. 3). Plus female mice had increased levels of ketone bodies (3-hydroxybutyric acid and acetoacetic acids). Of these compounds, 14 DAMs in both sexes were not only from the same lipid class but also had acyl chains of the same lengths and saturation: N-acylethanolamine 18:0; FA 17:0 and 18:1; lysophosphatidylcholine 18:0; phosphatidylcholines (PC) 15:0_15:0, 16:0_16:0, 16:0_16:1, 16:0_18:1, 18:1_18:1, 20:3_20:3, and 32:0; phosphatidylserine 40:6; phosphocholine, and SM d18:1_18:0 (Supplementary Fig. 3).

### 3.4. Omics analyses

**Fig. 2.**
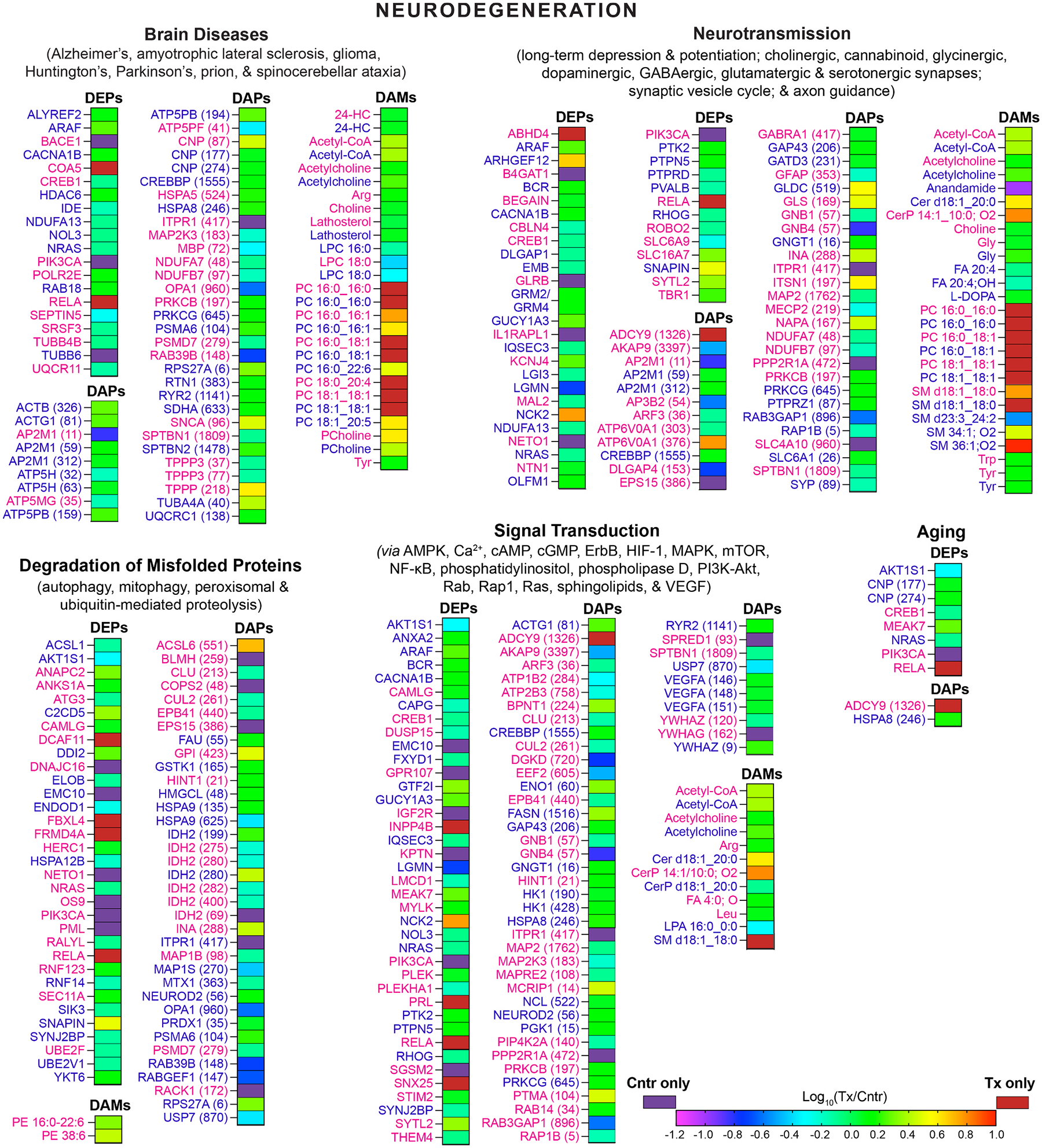
Multiomics enrichments in the neurodegeneration category. Integrated analysis (DEPs, DAPs, and DAMs) of female (magenta) and male (blue) EFV-treated *vs* control 5XFAD mice (n = 5 per sex and group) by MetaboAnalyst. Individual pathways contributing to each group significant enrichments (*P* ≤ 0.05) are in parenthesis below the group name. Data for male mice were obtained previously [31], and data for female mice were generated in the present work. The acetylation sites in DAPs are in parenthesis near the protein name. DAMs encompass those identified by metabolomics as well as by targeted compound quantifications. Protein and metabolite classification and abbreviation are according to UniProt and LIPID MAPS [52,201]. 24HC, 24-hydroxycholesterol; AMPK, AMP-activated protein kinase; cAMP, cyclic AMP; Cer, ceramide; Akt, protein kinase B; CerP, ceramide 1-phosphate; cGMP, cyclic GMP; ErbH, erythroblastic oncogene B; FA, fatty acid; HIF, hypoxia-inducible factor; L-DOPA, l-3,4-dihydroxyphenylalanine; LPA, lysophosphatidic acid; LPC, lysophosphatidyl choline; MAPK, mitogen-activated protein kinase; mTOR, mechanistic target of rapamycin; NF-κB, nuclear factor kappa B; P, phospho; PC, phosphatidyl choline; PCholine, phosphocholine; PE, phosphatidyl ethanolamine; PI3K, phosphoinositide 3-kinase; SM, sphingomyelin. See Supplementary Text 1 for the DEP and DAP abbreviations.

**Fig. 3.**
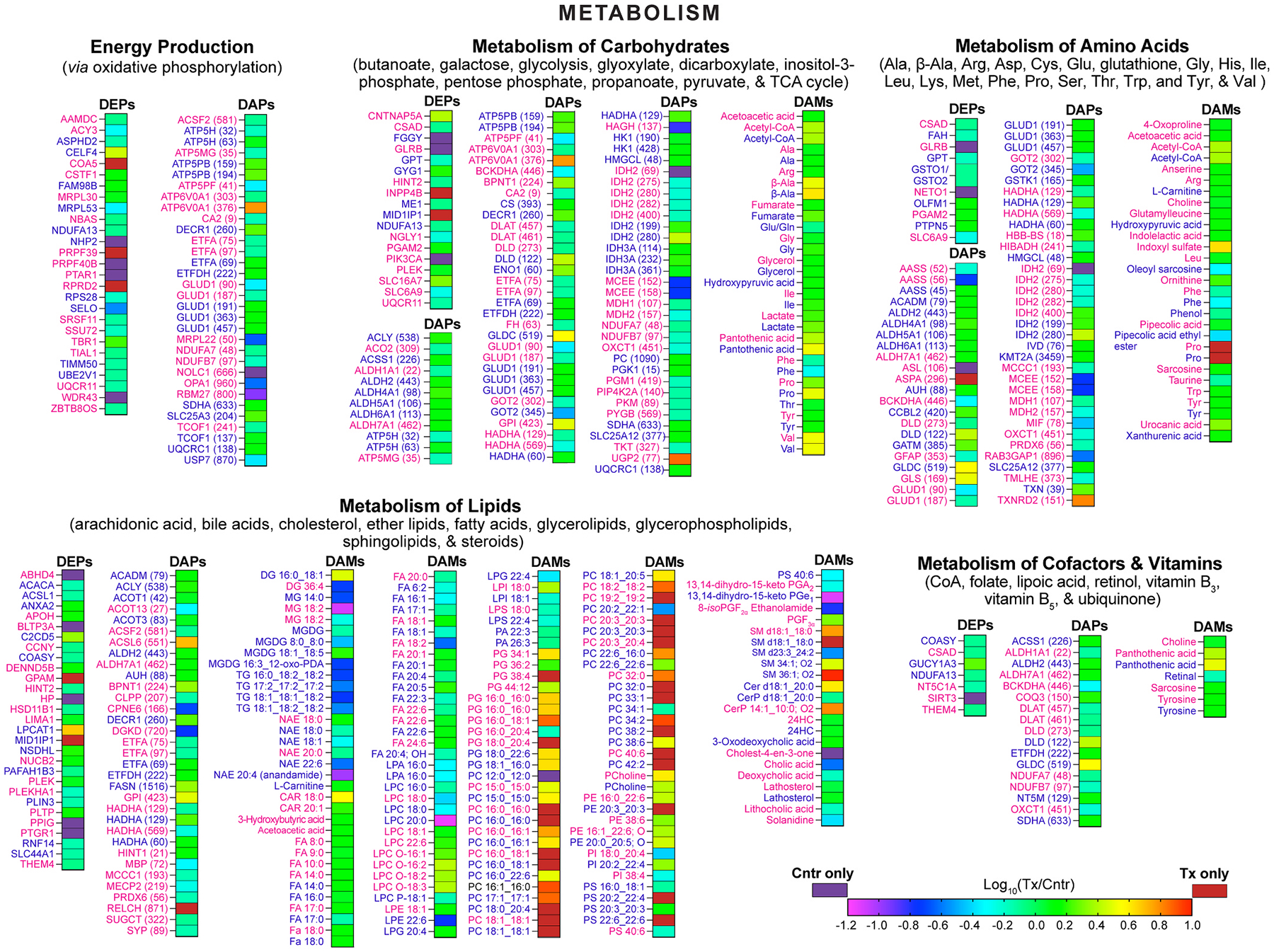
Multiomics enrichments in the metabolism category. Integrated analysis (DEPs, DAPs, and DAMs) of female (magenta) and male (blue) EFV-treated *vs* control 5XFAD mice (n = 5 per sex and group) by MetaboAnalyst. See Fig. 2 legend and Supplementary Text 1 for details and protein abbreviations. CAR, carnitine; DG, diacylglycerols; LPE, lysophosphatidylethanolamine; LPG, lysophosphatidylglycerol; LPI, lysophosphatidylinositol; LPS, lysophosphatidylserine; MG, monoacylglycerol; MGDG, monogalactosyldiacylglycerol; NAE, N-acyl ethanolamine; PA, phosphatidic acid; PDA, phytodienoic acid; PE, phosphatidylethanolamine; PG, phosphatidylglycerol; PGA_2_, prostaglandin A_2_; PGE_1_, prostaglandin E_1_; PGF, prostaglandin F; PI, phosphatidylinositol; PS, phosphatidylserine; TCA, tricarboxylic acid; TG, triacylglycerol.

**Fig. 4.**
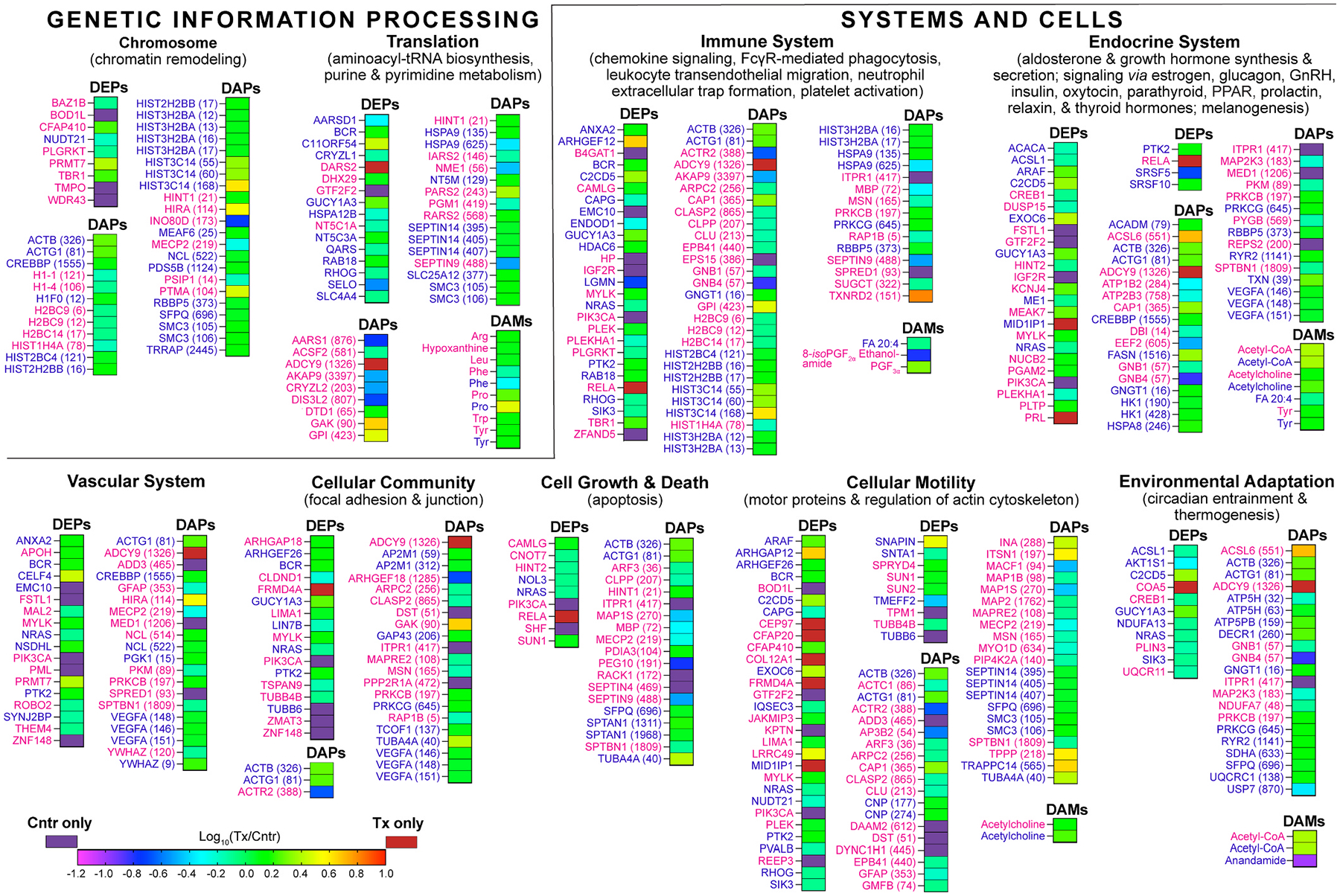
Multiomics enrichments in the genetic information processing and systems and cells categories. Integrated analysis (DEPs, DAPs, and DAMs) of female (magenta) and male (blue) EFV-treated *vs* control 5XFAD mice (n = 5 per sex and group) by MetaboAnalyst. See Fig. 2 legend and Supplementary Text 1 for details and protein abbreviations. GnRH, gonadotropin-releasing hormone; PPAR, peroxisome proliferator–activated receptors.

For each EFV-treated and control group, all differentially abundant molecules were pooled, separately for female mice and both sexes, and input into MetaboAnalyst, which can simultaneously analyze proteins and small molecules. Pathways with statistically significant enrichments were determined and combined into groups and four major categories (neurodegeneration, metabolism, genetic information processing, and systems and cells, Figs. 2–4) to obtain a broad view of EFV-treatment effects. The most notable proteins contributing to the pathways’ enrichments were identified as well (Supplementary Table 4).

In the neurodegeneration category, all enriched groups (various brain diseases, neurotransmission, degradation of misfolded proteins, and signal transduction) were the same for male [31] and female mice, except the aging group, which became significant only when both sexes were combined (Fig. 2). Similarly, all enriched groups in the metabolism category (energy production and metabolism of carbohydrates, lipids, amino acids, cofactors, and vitamins) overlapped between female and male mice as are in the category of genetic information processing (chromosome organization and translation) (Figs. 3 and 4). In the systems and cells category, sex-independent enrichments were in the vascular system, cellular community, cell growth and death, cellular motility, and environmental adaptation groups (Fig. 4). Female-specific enrichments related to immune and endocrine systems (signaling *via* estrogen, oxytocin, and prolactin).

The enrichment-contributing DEPs and DAPs were mostly different between the sexes, whereas DAMs were from the same metabolite classes. This suggested that some of the functional outcomes of the pathways’ enrichments could be similar in female and male mice, although mediated by different proteins as exemplified by the next two sections.

### 3.5. Notable proteins enriching the brain diseases group

In EFV-treated female mice, these proteins included BACE1, MBP, PIK3CA, RAB39B, and SNCA (Supplementary Table 4). BACE1 initiates the generation of Aβ peptides and is a prime target for slowing down Aβ production [58]. MBP is a major component of myelin sheath with its acetylation leading to myelin damage, observed in multiple sclerosis, AD, and other neurodegenerative diseases [59–61]. PIK3CA produces phosphatidylinositol 3,4,5-trisphosphate, activating cancer signaling in glioblastoma (an aggressive glioma type) [62–64]. RAB39B is involved in autophagy, and its mutations cause early onset PD and may contribute to etiology of amyotrophic lateral sclerosis and frontotemporal dementia [65–67]. The role of RAB39B acetylation is currently unknown. SNCA can aggregate and accumulate, a characteristic feature of synucleinopathies including PD, yet SNCA aggregation is decreased by acetylation [68]. Accordingly, a dramatic decrease in abundances of BACE1 and PIK3CA, increased SNCA acetylation and decreased MBP acetylation in EFV-treated female 5XFAD mice were likely all positive EFV-treatment effects.

In EFV-treated male mice, the notable affected proteins were different and included NOL3, NRAS, and RAB18. All three pertain to various cancers (Supplementary Table 4) and have increased expression in glioma [63,69–71]. Hence, decreased abundance of NOL3 and NRAS in male mice was likely a positive EFV effect, whereas increased abundance of RAB18 involved in intracellular membrane traffic [72] was possibly in response to increased brain cholesterol turnover due to EFV treatment.

In addition to sex-specific changes, the brain diseases group had enrichments with the proteins related to the shared hallmarks of neurodegenerative diseases - cytoskeletal dysregulation and decreased energy production [25]. In EFV-treated female mice, the cytoskeleton-related group was represented by proteins pertaining to filaments (SEPTIN5), spectrins (SPTBN1), and microtubules (TUBB4B and TPPP3). In EFV-treated male mice, this group included actins (ACTB and ACTG1), spectrin (SPTBN2), and tubulins (TUBA4A and TUBB6). The energy production group encompassed subunits from Complex 1 (NDUFA7 and NDUFB7), 3 (UQCR11), 4 (COA5), and 5 (ATP5MG and ATP5PF) of the electron transport chain in female mice, and subunits from Complex 1 (NDUFA13), 2 (SDHA), 3 (UQCRC1), and 5 (ATP5H and ATP5PB) in male mice (Supplementary Table 4).

Many of the protein acetylation effects in the cytoskeletal and energy production groups are not known [73]. Nevertheless, some of the detected changes were clearly normalizing (Supplementary Table 4). For, example, EFV-treated female mice had decreased abundance of SEPTIN5, increasing the Aβ levels and accumulated in dopaminergic neurons due to parkin dysfunction, leading to neurotoxicity [74,75]. Female mice also had increased abundance of COA5, essential for Complex 4 assembly [76]. EFV-treated male mice had increased acetylation of TUBA4A, which increases microtubule stability, impaired in AD and other neurodegenerative diseases [77]. Thus, we identified novel proteins and protein families that can putatively mediate the CYP46A1 activation effects [9,10,14,15,17–22] in mouse models of not only AD but also other brain disorders.

### 3.6. Notable proteins enriching the neurotransmission group

**Fig. 5.**
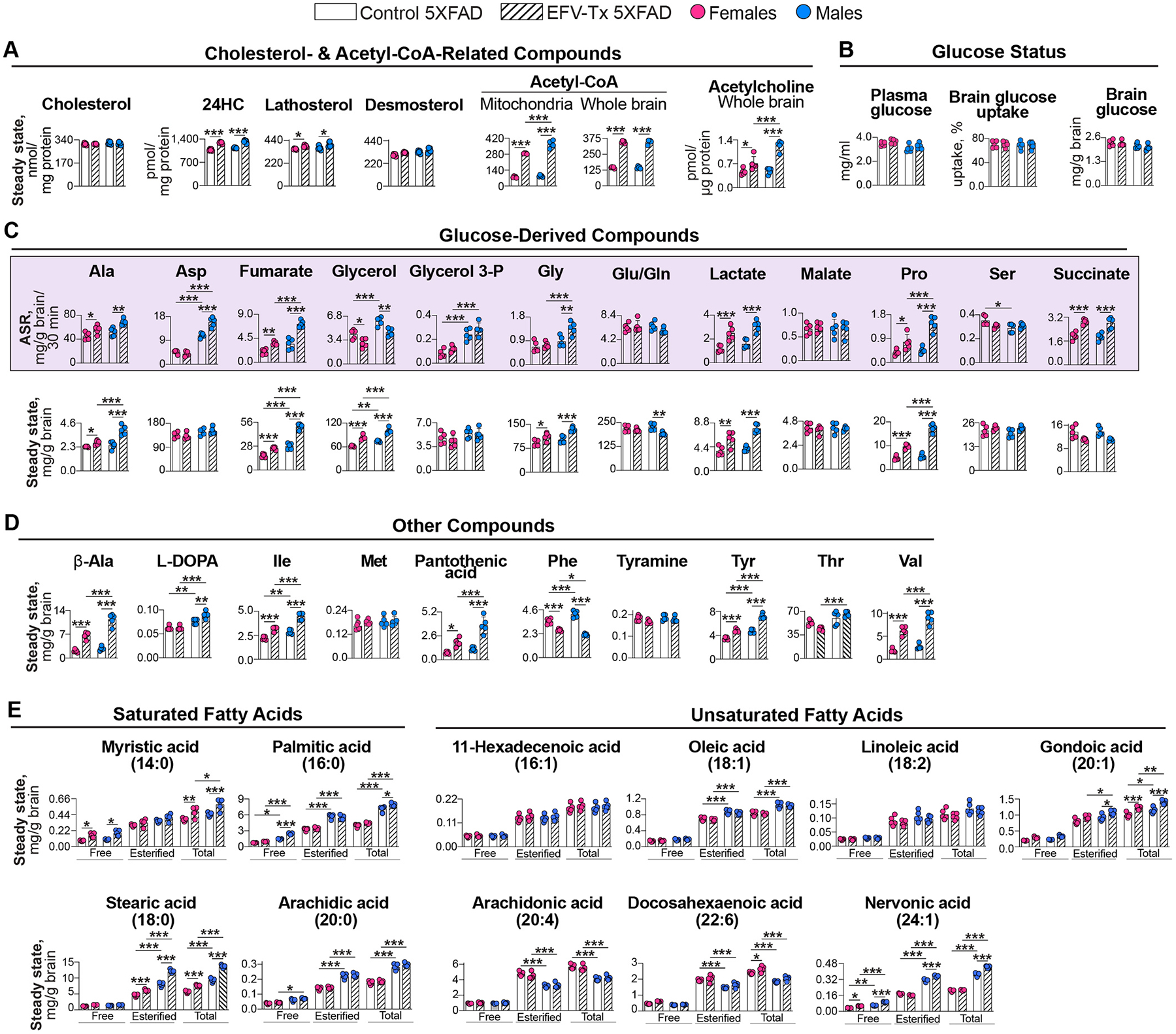
Targeted compound quantifications. Both the steady state levels and absolute synthesis rates (ASR, placed in lavender box) of various compounds were measured in the brain of EFV-treated *vs* control 5XFAD mice of both sexes (n = 5 per sex and group). Data represent the mean ± SD of the measurements in individual mice (magenta dots are female mice and blue dots are male mice). Data were analyzed by two-way ANOVA with Tukey’s multiple comparison test. *P ≤ 0.05; **P ≤ 0.01; ***P ≤ 0.001. Ala, alanine; β-Ala, β-alanine; Asp, aspartic acid; Gln, glutamine; Glu, glutamic acid; Gly, glycine; Glycerol-3-P, glycerol-3-phosphate; Ile, isoleucine; L-DOPA, levodopa (l-3,4-dihydroxyphenylalanine); Met, methionine; Phe, phenylalanine; Pro, proline; Ser, serine; Thr, threonine; Tyr, tyrosine; Val, valine.

EFV is mainly thought to enhance glutamatergic neurotransmission as 24HC is a positive allosteric modulator of NMDARs [34,40,78] activated by Glu and its co-agonist Gly [79]. EFV-treated female mice had unchanged levels of glutamate/glutamine (Glu/Gln, measured as one pool, Fig. 5), despite increased acetylation of GLS (Fig. 2, Supplementary Table 4), which decreases enzyme activity and hence Glu synthesis [80,81]. Conversely, EFV-treated male mice had decreased levels of Glu/Gln (Fig. 5), consistent with increased abundance of GRM2 and/or GRM4 (Fig. 2, Supplementary Table 4), activated by Glu and suppressing excessive neurotransmission by inhibiting Glu release [82]. In addition, both sexes had effects on the DLGAP isoforms (decreased acetylation of DLGAP4 in female mice and decreased abundance of DLGAP1 in male mice, Fig. 2, Supplementary Table 4), involved in synaptic scaling or reset of neuronal firing of glutamatergic neurons to “normal” levels [83]. Plus, EFV-treated 5XFAD female mice had decreased expression of NETO1, required for normal abundance of NMDARs [84]. Thus, both sexes of EFV-treated mice had effects on the proteins that may be protective against overstimulation of glutamatergic transmission.

Glycine (Gly) is not only a co-agonist of NMDARs but also is an agonist of Gly receptors, a dual role providing a balanced regulation between the excitatory glutamatergic and inhibitory glycinergic neurotransmission [85]. The levels of Gly were increased in both sexes of EFV-treated mice (Fig. 5), and protein changes related to glycinergic neurotransmission were consistent with this increase (Supplementary Table 4). In EFV-treated female mice, GLRB, which anchors Gly receptors at synaptic sites [85], was only detected in the control group, whereas the abundance of SLC6A9, which removes Gly from the synaptic cleft, was decreased, perhaps to compensate for a dramatic reduction of GLRB. In EFV-treated male mice, increased levels of Gly could be due to GLDC, initiating Gly breakdown but having increased acetylation (Fig. 2, Supplementary Table 4), which impairs enzyme activity [86,87]. Thus, like glutamatergic transmission, the glycinergic neurotransmission could be affected by EFV treatment as well.

Besides Gly, Glu signaling can be affected by the endocannabinoid anandamide (Fig. 2), an agonist for the brain cannabinoid receptor CB1 [88]. Anandamide can co-activate both CB1 and NMDARs and induce long-term depression and/or only interact with NMDARs and enhance receptor activity [89]. Neither control nor EFV-treated female mice had detectable anandamide levels, and ABHD4, regulating the production of anandamide [90], was only detected in control female mice (Fig. 2). Conversely, control and EFV-treated male mice had detectable anandamide levels, which were reduced 10-fold in EFV-treated mice, but ABHD4 was not detected in either group (Fig. 3). Apparently, female and male 5XFAD mice had different basal levels of anandamide and ABHD4 but could have the same EFV effect leading to a reduced anandamide production.

Finally, for the brain to function normally, the excitatory glutamatergic neurotransmission should be balanced by the inhibitory GABAergic neurotransmission [91], closely linked to dopaminergic neurotransmission (both excitatory and inhibitory) as GABA receptors are present on dopaminergic terminals [92]. While GABA and dopamine were not among the annotated molecules in our studies, EFV-treated female mice had reduced expression of CBLN4, a trans-synaptic cell-adhesion molecule that contributes to the formation and/or function of GABAergic synapses [93]; increased acetylation of GABRA1, a GABA_A_ receptor subunit contributing to the synaptic contact formation [94]; and decreased acetylation of SLC4A10, which promotes synaptic GABA release [95]. Yet, only increased acetylation of SLC6A1, which reuptakes GABA from the synapse [96] was detected in EFV-treated male mice (Supplementary Table 4), and the functional effects of these acetylations are not yet known. Nevertheless, the GABAergic and dopaminergic neurotransmissions could be affected by EFV treatment as overexpression of *CYP46A1* in mice was shown to promote their midbrain dopaminergic neurogenesis [97]. In any case, the multiomics enrichments suggested that several neurotransmission types (glutamatergic, glycinergic, GABAergic and endocannabinoid signaling) could be affected by EFV treatment.

### 3.7. Targeted quantifications

We repeated sterol quantifications to confirm EFV effects [10] on the brain levels of two cholesterol precursors (lathosterol and desmosterol), cholesterol, and 24HC, which is only generated by CYP46A1 (Fig. 5A). The levels of each sterol in each group (control and EFV-treated) were similar in female and male mice, documenting lack of sex-based differences. As previously [10], EFV treatment increased the levels of 24HC and lathosterol, a marker of cholesterol biosynthesis in neurons [98] but did not affect the brain cholesterol levels. This data confirmed CYP46A1 activation and an increase in brain cholesterol turnover. We also confirmed increases in EFV-treated mice of both sexes in the mitochondrial and whole brain acetyl-CoA levels and the whole brain levels of acetylcholine, which is synthesized from choline and acetyl-CoA (Fig. 5A).

We quantified additional compounds to ascertain the functional outcomes of the identified enrichments in EFV-treated mice in the brain pathways of glycolysis, tricarboxylic acid (TCA) cycle, fatty acid metabolism, and amino acid metabolism, all tightly linked energy-related pathways. We injected both sexes of EFV-treated and control 5XFAD mice with [U-^13^C_6_]-glucose as glucose from the systemic circulation is the primary energy source for the brain [99,100]. We then processed brain samples to obtain two extraction phases - aqueous and organic. The aqueous phase was mainly used to quantify the absolute synthesis rates (ASR) and steady state levels of glucose-derived metabolites and amino acids. The organic phase was mainly used to determine the levels of fatty acids.

**Fig. 6.**
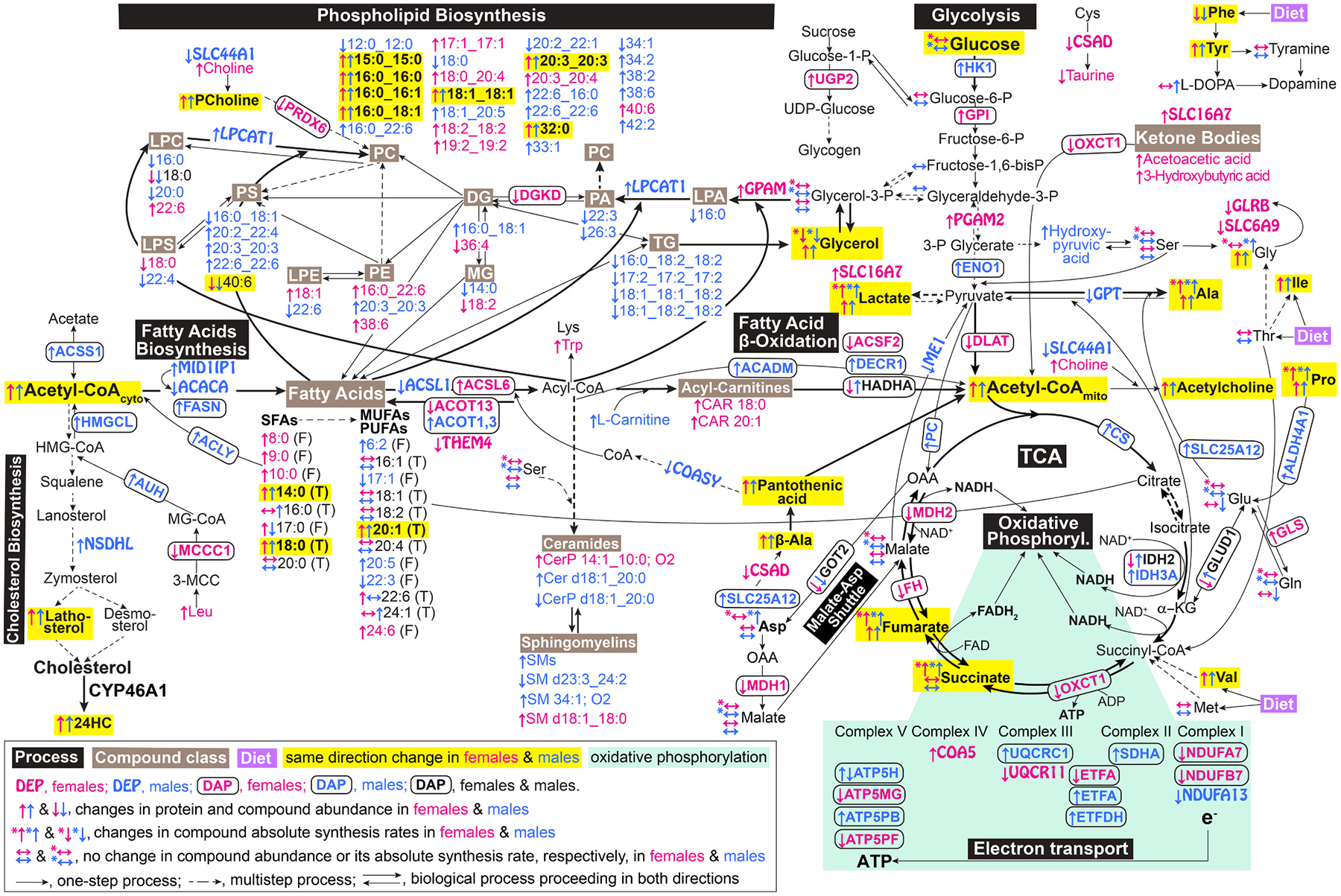
EFV effects on brain energy landscape in 5XFAD mice. A summary of the multiomics and targeted quantifications in Figs. 2–5(n = 5 per sex and group). Inset at the bottom left corner shows annotations for colored boxes, fonts, and arrows. Names for processes are in black boxes; names for compound classes are in tan boxes with acyl chains indicated below; the source (diet) of essential amino acids is in lavender box; changes of the same directionality in both sexes are in yellow boxes, and molecules involved in oxidative phosphorylation are on a green background. ↑ (increase) and ↓ (decrease), changes in EFV-treated *vs* control 5XFAD female (magenta) and male mice (blue) leading to differential protein expression (unboxed protein names), protein acetylation (protein names are in rounded rectangles) or compound abundance. ↑* (increase), ↓* (decrease), changes in compound absolute synthesis rate. ↔, no effect on compound abundance; ↔*, no effect on compound absolute synthesis rate. →, one-step process; ⇢, multistep process, ⇄, biological process proceeding in both directions. Thick lines link the major affected processes. F, free fatty acids; T, total fatty acids. See Figs. 2–5 legends and the Supplementary Text 1 for the abbreviations.

EFV treatment did not change plasma glucose levels, brain glucose uptake, and brain glucose levels (Fig. 5B). Therefore, we focused on the measurements of the ASRs and levels of glucose metabolites produced as a result of glycolysis branching (Fig. 6). Indeed, both sexes of EFV-treated mice had decreased glycerol ASRs indicating decreased glycolysis branching from fructose-1,6-bisphosphate and glyceraldehyde-3-phosphate to glycerol *via* glycerol-3-phosphate (Figs. 5B,6). Yet the glycerol steady state levels were increased (Fig. 5C), likely a reflection of increased glycerol production from tri-, di-, and monoglycerides as their levels were decreased in EFV-treated mice (Figs. 3,6). Conversely, branching from 3-phosphoglycerate to serine (Ser) was not affected in both sexes but was increased to Gly (Fig. 5C) in EFV-treated male mice, which also had increased levels of hydroxypyruvic acid (Fig. 3) and Gly along with increased Gly ASR (Figs. 5C,6).

The most notable glycolysis branching was from pyruvate to lactate as the lactate ASRs and levels were substantially (>2-fold) increased in EFV-treated mice of both sexes (Figs. 5C, 6). In the adult brain, lactate is believed to be predominantly synthesized in astrocytes but could be transferred to neurons, upon their activation, to be converted back to pyruvate and acetyl-CoA and fuel neuronal TCA cycle and fatty acid synthesis [101–104]. If so, increased lactate ASRs and levels in EFV-treated mice of both sexes along with their increased acetyl-CoA levels (both mitochondrial and whole brain, Fig. 5A) suggested that the lactate flux from astrocytes to neurons was indeed increased, consistent with increased abundance in female mice of SLC16A7 (or monocarboxylate transporter 2, Fig. 3), importing lactate and other monocarboxylates (pyruvate and ketone bodies) into neurons [100,105].

In addition, EFV treatment seemed to increase glycolysis branching from pyruvate to alanine (Ala) as its ASRs and levels were increased in both sexes (Fig. 5C), despite decreased abundance in male mice of GPT (glutamic-pyruvic transaminase or alanine aminotransferase 1, Fig. 3), which catalyzes reversible conversion of pyruvate to Ala (Fig. 6). This decrease along with increased Ala levels suggested a non-enzymatic conversion of pyruvate to Ala, but the GPT-mediated conversion of Ala to pyruvate. The latter is consistent with a transfer of not only lactate but Ala as well from astrocytes to neurons, where it is converted back to pyruvate by GPT to increase neuronal energy production [106].

Besides enhanced glycolysis branching, increased energy production was supported by the changes in the TCA cycle, namely sex-independent increases in the ASRs of succinate and fumarate (Fig. 5C). These increases were indicative of increased metabolite flux through the cycle and hence increased generation of FADH_2_ used for oxidative phosphorylation (Fig. 6). The ASRs of malate were unchanged, whereas those of Asp were increased, at least in male mice (Fig. 5C), and both sexes had increased levels of β-Ala and pantothenic acid (Fig. 5D). Changes in the latter two suggested increased flux through the malate-Asp shuttle, leading to increased synthesis of pantothenic acid (or vitamin B_5_), which could serve as an additional substrate for increased acetyl-CoA formation (Fig. 6).

Acetyl-CoA and energy can be obtained from β-oxidation of fatty acids (Fig. 6), some of which were detected and quantified in 5XFAD mice. EFV treatment led to sex-independent increases in the levels of free and total myristic acid, esterified and total stearic acid, total gondoic acid, and free nervonic acid (Fig. 5E). In addition, there was a male-specific increase in the levels of free and total palmitic acid, and a female-specific increase in the levels of total docosahexaenoic acid. These increases, especially those of abundant fatty acids – palmitic and stearic (Fig. 5E), suggested that fatty acids could be an additional source of increased energy production in EFV-treated mice,

Finally, we extended the amino acids quantifications from Ala, aspartate (Asp), Gly, Glu/Gln, and Ser produced from glycolysis or TCA cycle branching to several other amino acids. These were both non-essential amino acids [proline (Pro) and tyrosine (Tyr)], i.e. those that can be synthesized in the body, and essential amino acids [isoleucine (Ile), methionine (Met), phenylalanine (Phe), threonine (Thr), and valine (Val)], i.e. those that must be obtained from diet. Of them, EFV-treatment increased in both sexes of 5XFAD mice the ASRs and levels of Pro (Fig. 5C), a precursor of the neurotransmitter Glu and α-ketoglutarate in the TCA cycle (Fig. 6), and the levels of Ile and Val that feed into the TCA cycle at the succinate level and stimulate energy productions and protein synthesis [91,107] (Figs. 5D,6). Conversely, both sexes of 5XFAD mice had decreased levels of Phe converted to the neurotransmitter dopamine *via* metabolism to Tyr and then either L-DOPA (l-3, 4-dihydroxyphenylalanine) or tyramine (Fig. 5D). Both sexes of EFV-treated mice had increased levels of Tyr and unchanged levels of tyramine, and male mice had increased levels of L-DOPA (Figs. 5D, 6), suggesting potential effect on the production of dopamine, which, however, was not annotated in our quantifications. Thus, of the 12 amino acids that were quantified in EFV-treated mice, almost all (except Glu/Gln) had the same directionality of changs in both sexes: six (Ala, Gly, Ile, Pro, Tyr, and Val) were at increased levels, one (Phe) was at decreased levels, and the levels of the remaining four (Asp, Met, Ser, and Thr) were unchanged. Accordingly, processes (protein synthesis, energy production, and neurotransmission) that depend on the affected amino acids could be altered in the brain of EFV-treated mice as well [107–109].

### 3.8. Brain vasculature

Subjects with AD have cerebrovascular dysfunction with manifestations including a decrease in cerebral blood flow (cerebral hypoperfusion), changes in cerebral vessel density and shape, and compromised integrity of the blood-brain barrier (BBB) [110–113]. Cerebrovascular dysfunction is also present in 5XFAD mice as early as 6 months of age as indicated by their cerebral hypoperfusion, cerebrovascular deficits, and cerebrovascular structural changes [114,115]. Lactate was recently shown to promote angiogenesis in the developing mouse cortex [116], and we documented increased lactate levels in both sexes of EFV-treated mice (Fig. 5C). In addition, the vascular system was among the enriched groups in the multiomics analysis (Fig. 4, Supplementary Table 4). Therefore, we evaluated EFV for the brain vasculature effects in 5XFAD mice as this was not investigated previously. Several tests were used: 1) *in vivo* brain ultrasound assessment or angio 3D tomography to visualize the brain vascular tree; and 2) fluorescence imaging after retro-orbital dye injections of mice with Evans blue (a marker of BBB integrity) and FITC-dextran (a marker of blood vessels) [55,117, 118]. In the dye-injected mice, *in vivo* imaging of brain surface was conducted first followed by imaging of brain cross sections.

**Fig. 7.**
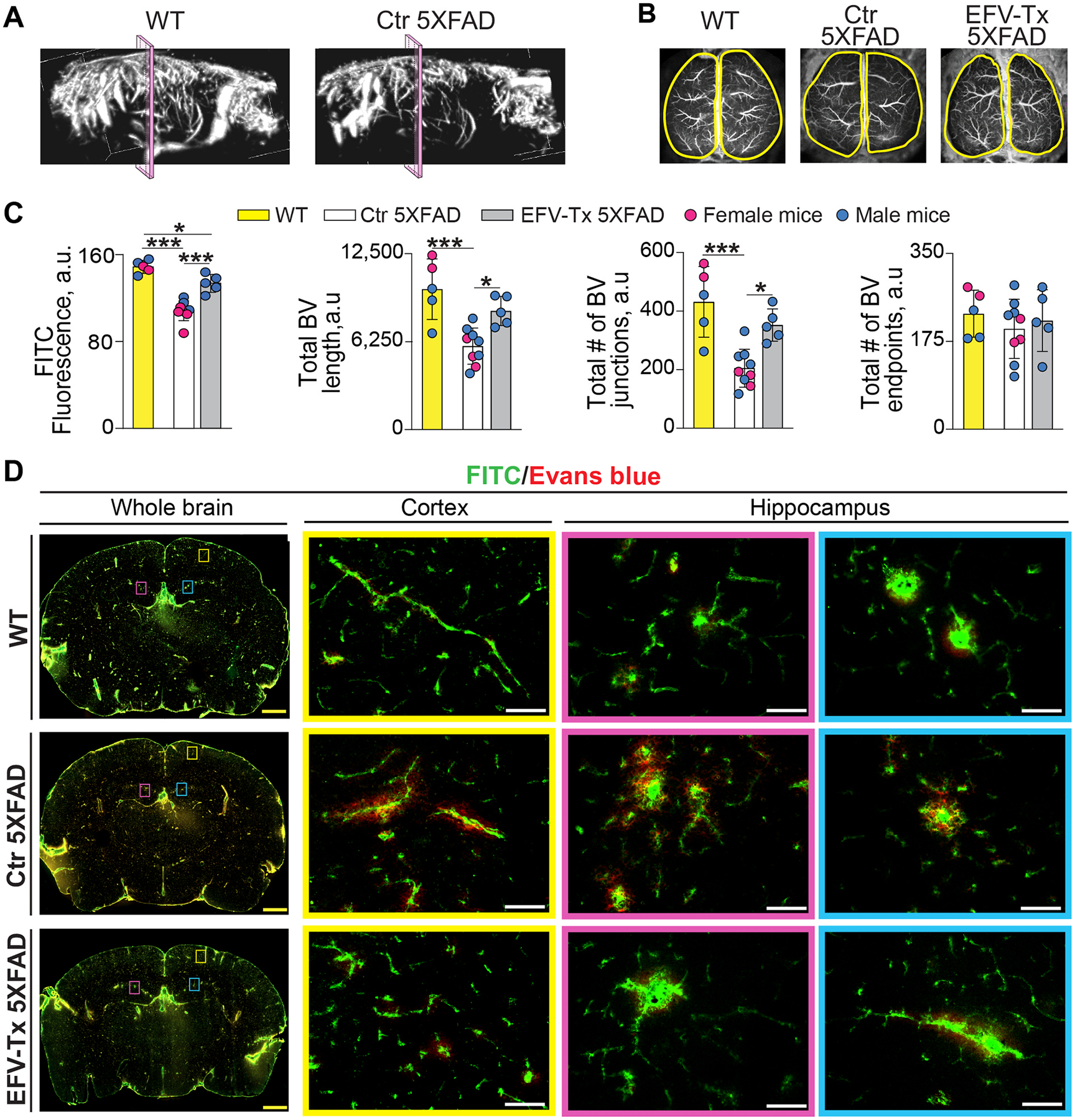
EFV effect on brain vasculature in 5XFAD mice. A: Brain vascular tree of wild type (WT) and control (Ctr) 5XFAD mice as visualized by ultrasound imaging or angio 3D tomography. Pink planes show the area of coronal brain sectioning in D. B: Brain surface vasculature as visualized by fluorescein isothiocyanate (FITC) angiography after sequential retro-orbital injections of FITC and Evans Blue to WT mice (2 female and 3 male mice), Ctr 5XFAD mice (4 female and 5 male mice), and EFV-treated (Tx) 5XFAD mice (5 male mice). C: The quantification of FITC fluorescence and blood vessel (BV) parameters in B; magenta circles are female mice and blue circles are male mice. Data were analyzed by one-way ANOVA with Tukey’s multiple comparison test. *P ≤ 0.05; ***P ≤ 0.001. D: Status of the blood brain barrier as assessed by FITC (green) and Evans blue (red) fluorescence of coronal brain sections of WT, Ctr 5XFAD mice, and EFV-Tx 5XFAD mice (the number of mice per group and sex are the same as in B). Colored rectangles denote enlarged regions of the whole brain. Yellow scale bars 1000 μm; white scale bar 50 μm.

On angio 3D tomography, the vascular tree was apparently less dense in control 5XFAD *vs* WT mice, and some of the branches in the former seemed to be missing (Fig. 7A). These findings were consistent with cerebrovascular density deficits previously documented in 5XFAD mice [115] and AD-affected individuals [113,119] and suggested that our cohort of 5XFAD mice also had these deficits. Hence, next three groups of mice were used (WT, control 5XFAD, and EFV-treated 5XFAD), and EFV effects were evaluated after the dye injections. On FITC angiography, a sex-independent decrease in brain surface fluorescence was observed in control 5XFAD *vs* WT mice (Fig. 7B,C), consistent with the angio tomography findings. Conversely, in EFV-treated *vs* control 5XFAD mice, the surface FITC fluorescence was increased, although did not reach the WT levels (Fig. 7B,C), indicating the rescuing effect. Then, we analyzed the architecture of cerebral vasculature by quantifying the total blood vessel lengths (reflects the extent of vascular coverage), total number of blood vessel junctions (represents branching complexity, essential for efficient perfusion), and total number of blood vessel endpoints (represents incomplete vessel connections, characteristic of pathologic neovascularization) [120]. The total vessel lengths and total number of junctions were decreased in control 5XFAD *vs* WT mice and increased in EFV-treated *vs* control 5XFAD (Fig. 7C), indicating impaired brain vascularization and cerebral hypoperfusion in control 5XFAD mice and restoration of these functions in EFV-treated 5XFAD mice. Yet no differences between the three groups of mice were detected in the total number of blood vessel endpoints (Fig. 7C) suggesting lack of pathologic neovascularization.

Imaging of coronal brain sections from the dye-injected mice was done next to evaluate their BBB integrity. In WT mice, the BBB appeared to be intact as no Evans blue fluorescence was detected beyond the FITC fluorescence (Fig. 7D–F). Conversely, in control 5XFAD mice, some of the brain blood vessels had leakage of Evans blue suggesting focal impairments in the BBB function or possibly cerebral microbleeds, known to occur in AD and usually detected by the magnetic resonance imaging [121]. In any case, these focal impairments seemed to be ameliorated in EFV-treated mice as the Evan blue leakage was much smaller in this group than in control mice (Fig. 7D–F). Thus, both cerebrovascular architecture and the BBB leakage were rescused in part in EFV-treated 5XFAD mice, identifying a novel, previously unconsidered EFV therapeutic effect.

## 4. Discussion

Sex and gender differences were reported for several neurodegenerative diseases [122,123] with sex being a major risk factor for AD, affecting women twice as much as men [124]. Healthy women have a considerably higher brain CYP46A1 abundance than men [125], and their CYP46A1 activity in menopause correlates with bioavailable plasma estrone and estradiol levels [126]. The unexpected, yet explainable finding of the present study was not the identification of the female-specific enrichments pertaining to immune system and hormones (estrogen, oxytocin, and prolactin, Fig. 4), which are at higher levels in women than in men and are affected by age [127–129]. Rather, these were different proteins in female and male mice that mediated enrichments of the same pathways (Figs. 2–4). In part, this could be because EFV-treated 5XFAD female and male mice had opposite changes in brain content of estradiol and possibly other steroid hormones. Indeed, previously we found that in male 5XFAD mice, EFV treatment decreased their brain estradiol levels [11], whereas in a study of C57BL/6N female mice with transgenic *CYP46A1* overexpression, brain estrogen signaling was enhanced [130]. Sex hormones are known to mediate their action *via* genomic and non-genomic effects, the former affecting gene expression and the latter activating various signaling pathways (distinct and overlapping in females and males) after binding to estrogen, androgen, and progesterone receptors [131]. Therefore, sex-specific EFV effects on brain levels of estradiol and possibly other neurosteroids were likely due to sex-specific drug effects on brain gene expression and proteins in the affected signaling pathways. Further studies are needed to test this explanation.

The sex-independent increase in brain energy production was perhaps one of the most important EFV treatment effects documented in the present study. Metabolite quantifications indicated that EFV-treated 5XFAD mice had increased, irrespective of sex, branching from glycolysis, especially from pyruvate to lactate and Ala, plus increased carbon flux thorough the TCA cycle and hence subsequent oxidative phosphorylation. Altered abundance of the enzymes involved in glycolysis (PGAM2), astrocyte to neuron lactate shuttle (SLC16A7), TCA cycle (MEI), and oxidative phosphorylation (UQCR11 and COA5) (Fig. 6) was consistent with this inference and pointed to the proteins mediating this increase. Specifically, increased expression of PGAM2 (Fig. 6, female mice) increases the acetyl-CoA levels [132]; increased expression of SLC16A7 increases lactate import into the neurons [133]; and decreased expression of ME1 (Fig. 6, male mice) increases oxidative phosphorylation [134]. Similarly, increased expression of COA5 (Fig. 6, female mice,), essential for the mitochondrial complex 4 assembly [76], can increase oxidative phosphorylation and compensate for a decreased expression of UQCR11, which can decrease oxidative phosphorylation [135]. EFV treatment effects on the acetylation of the enzymes related to glycolysis (HK1, GPI, ENO1), acetyl-CoA formation from pyruvate (DLAT), and TCA cycle (CS, IDH2, OXCT1, FH, an MDH) were also documented (Fig. 6). However, the net effect of these acetylations, especially when they are at multiple sites of multiple enzymes, are difficult to predict, although the functional consequences of the single enzyme acetylations are known [136–140].

Of the energy sources alternative to glucose, fatty acids account for ~20% of brain energy production by β-oxidation, which generates acetyl-CoA [141]. Also, when brain glucose levels are low, ketone bodies, which are delivered from the liver, as well as certain amino acids, essential and non-essential, represent additional energy sources [100,142]. Both sexes of EFV-treated mice had increased levels of abundant stearic and less abundant myristic fatty acids (Fig. 5E) plus carnitine (male mice) or fatty acylcarnitines (female mice) (Fig. 3) required for fatty acid β-oxidation (Fig. 6). Both sexes also had increased levels of the energy-contributing Ala, Val, Ile, and β-Ala (Fig. 5C,D) [107,109]. In addition, EFV-treated female mice had increased brain levels of acetoacetic and 3-hydroxybutyric acids, the two main ketone bodies (Fig. 3). Thus, the levels of alternative energy sources were increased in the brain of EFV-treated mice, suggesting increased metabolic flexibility (Fig. 6). Conversely, decreased metabolic flexibility and energy production are hallmarks of AD and other neurodegenerative brain disorders [25,100,143] that contribute to disease etiology [144, 145]. As such, they are targeted by developing therapeutics [100,146], and EFV now should be among these therapeutics.

**Fig. 8.**
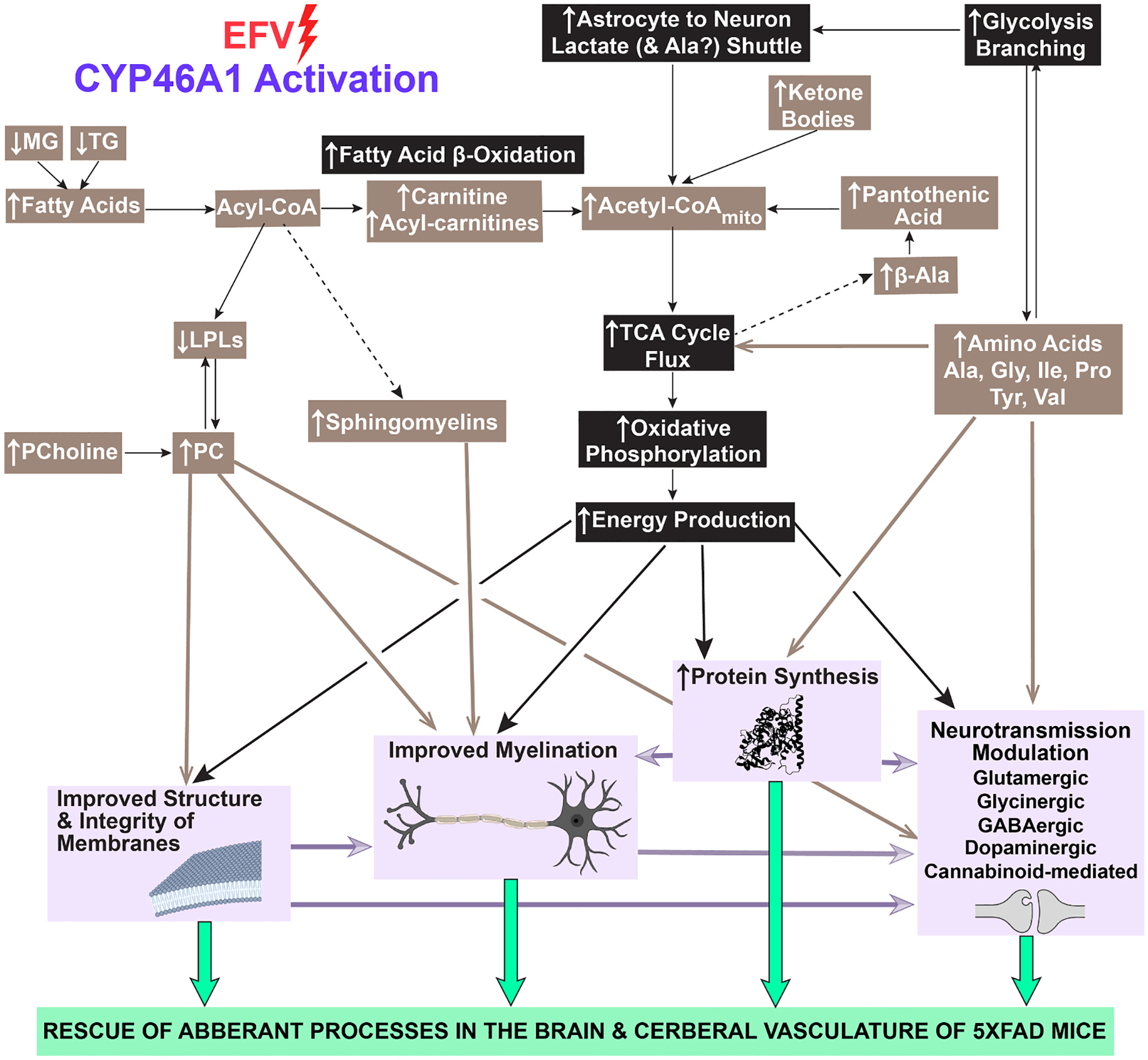
EFV effect on key brain molecules and processes. A summary of major changes in the brain of EFV-treated *vs* control 5XFAD suggested by the present work. See main text for details. Metabolic processes are in black boxes and more general brain processes are in lavender and green boxes; compounds are in tan boxes; ↑ and ↓ indicate increase and decrease, respectively; dotted arrows indicate multiple steps. LPLs, lysophospholipids; MG, monoacylglycerols; mito, mitochondrial; PCholine, phosphocholine; PC, phosphatidyl choline; TCA, tricarboxylic acid; TG, triacylglycerols.

Of unquestionable importance were increased levels in both sexes of EFV-treated mice of certain glycerophospholipids, sphingolipids, and essential amino acids (Figs. 3,5). Glycerophospholipids and sphingolipids are the two major phospholipid classes in the brain [147] with the glycerophospholipid PC being a major component of brain cell membranes, accounting for 30–35% of all phospholipids in the brain and 50% of phospholipids in neuronal membranes [147,148]. Besides this structural role, PC also serves as a source of acetylcholine and thereby supports cholinergic neurotransmission [148]. Accordingly, increasing the brain PC content is considered beneficial for AD [148] as the PC levels and cholinergic function are decreased in the affected regions of human [148–150] and 5XFAD brain [151]. EFV treatment increased in both sexes of 5XFAD mice the synthesis of various brain PC species from lysophospholipids, including dramatic increases in the levels of PC 16:0_18:1 (Fig. 3), the most abundant brain PC species [147]. This increase was consistent with a sex-independent increase in the levels of phosphocholine, a PC precursor (Fig. 3) [152], and dramatic increases in GPAM abundance in female mice and LPCAT abundance in male mice (Fig. 3), enzymes in the main pathway of PC production (Fig. 6) [153]. Thus, EFV treatment led to an overall increase in the PC content in the brain, likely improving the structure and integrity of neuronal and other membranes and contributing to the normalization of aberrant neurotransmission (Fig. 8). The latter is supported by improved performance of EFV-treated mice in memory task documented previously [10] and could contribute, along with increased acetyl-CoA production, to the observed increases in the acetylcholine levels (Fig. 5A).

The sphingolipid SM accounts for up to 12.5% of the brain phospholipid content and is a major lipid component of myelin sheaths [147, 154], which facilitate impulse propagation 20–100-fold as compared to nonmyelinated axons of the same diameter [155]. Abnormalities of white matter, mainly composed of myelinated axons, are common in AD and 5XFAD mice and include axonal demyelination [156–160]. Both sexes of EFV-treated mice had substantially increased levels of SM d18:1_18:0 (Fig. 3), the most abundant SM species in the brain [147]. Accordingly, this change, along with increased abundance of the PC species, suggested that lipid availability for remyelination was increased in EFV-treated mice. Protein availability for myelin repair seemed to increase in EFV-treated mice of both sexes as well as suggested by increased brain levels of Ile and Val (Fig. 5D), which stimulate protein synthesis [107,161]. Plus, EFV treatment could reduce myelin damage in female mice by decreasing the acetylation of the MBP Lys residues (Supplementary Table 4), which hold together myelin layers through electrostatic interactions with the negatively charged myelin lipids [59,61]. Thus, collectively, our data suggested that axonal myelination was likely improved in the brain of EFV-treated mice (Fig. 8).

Neurotransmission accounts for up to 80% of the brain energy requirements [100]. Therefore, EFV effects on several types of neurotransmission were supported by both increased energy production and changes in the metabolite and protein levels. Effects on glutamatergic neurotransmission were indicated by sex-independent increases in the levels of 24HC and Gly (Fig. 5A,C), both acting on NMDARs, and by altered abundance or acetylation of DLGAP4, GLS, and NETO1 in female mice or DLGAP1 and GRM2/GRM4 in male mice (Supplementary Table 4). Sex-independent changes pertaining to glycinergic neurotransmission included increased Gly levels (Fig. 5C) plus altered abundance of GLRB and SLC6A9 in female mice or GLDC in male mice (Supplementary Table 4). Endocannabinoid retrograde signaling was likely affected by non-detectable anandamide levels in EFV-treated male mice and non-detectable expression of ABHD4 in female mice EFV effects on the closely linked GABA and dopaminergic neurotransmissions were suggested by a sex-independent decrease in the Phe levels and increase in the Tyr levels, increased L-DOPA levels in male mice (Fig. 6) as well as altered abundance of CBLN4 and acetylation of GABRA1 and SLC4A10 in female mice and SLC6A1 in male mice (Supplementary Table 4). Finally, both sexes of EFV-treated mice had increased levels of acetylcholine (Fig. 5A) mediating cholinergic neurotransmission.

Excessive stimulation of glutamatergic neurotransmission can lead to excitotoxicity [162], and our study suggested protective mechanisms in EFV-treated mice against this cytotoxicity. Remarkably, these mechanisms seem to extend beyond the putative effects on glutamatergic neurotransmission (synaptic scaling, NMDAR abundance, Glu production and release) and involve effects on other neurotransmission types: glycinergic (increased Gly levels and altered Gly receptor abundance); GABAergic and dopaminergic (altered synapse formation, synapse function, GABA release, and GABA reuptake); cholinergic (increased acetylcholine levels); and endocannabinoid retrograde signaling (reduced anandamide levels) (Supplementary Table 4). Further studies are required to ascertain EFV effects on the interplay between all these neurotransmission types. In the meantime, we obtained insights into why increased CYP46A1 activity led to neurological improvements in mouse models of AD, Huntington’s disease, amyotrophic lateral sclerosis, and spinocerebellar ataxia type 3 that affect, in a disease-specific manner, each of these neurotransmission types [10,163–169].

Cerebrovascular dysfunction is an overlooked aspect of AD [170], although it increases the AD risk and develops before Aβ accumulation and cognitive decline [170]. Herein we showed that cerebrovascular density loss, cerebral hypoperfusion, and focal BBB dysfunction, known to be present in 5XFAD mice [114,115,171] and also detected in our cohort of mice, were mitigated in both sexes of EFV-treated 5XFAD mice (Fig. 7B–D). In addition, the vascular architecture analysis suggested that the brain angiogenesis was not prominent either in control or EFV-treated mice, at least at 9 months of age when they were evaluated. This was consistent with EFV-treated female mice having undetectable expression of angiogenic FSTL1 [172] and PIK3CA and EFV-treated male mice having increased acetylation of angiogenic VEGFA [173], probably diminishing VEFA signaling [174,175], and undetectable expression of angiogenic EMC10 [176] (vascular system, Fig. 4, Supplementary Table 4). Also, we did not detect increased HIF1α and VEGFA expression, reported to underlie the lactate-mediated angiogenesis increase in the developing mouse neocortex [116].

Rather, our multiomics studies pointed to the processes other than neovascularization that likely contributed to the normalization of cerebrovascular pathologies in EFV-treated 5XFAD. Indeed, both sexes of EFV-treated mice had functional enrichments in chemokine signaling, FcγR-mediated phagocytosis, neutrophil extracellular trap formation, and platelet activation grouped under immune system in the systems and cells category (Fig. 4). While distinct, these processes are regulated by the overlapping signaling cascades, and all can contribute to remodeling of cerebral vasculature [177–180]. Potential EFV effects on immune response in this work are consistent with a recent study in mice showing that EFV treatment initiated during the subacute phase of ischemic stroke promoted neurorestoration by the mitigation, among other processes, of neuroinflammation [181]. In addition, we found that in EFV-treated female mice, the abundance of ZNF148 linked to vascular malformations [182,183] became non-detectable, while the expression of MYLK contributing to barrier function in microvascular endothelium [184] and PRMT7 ameliorating cerebrovascular damage in traumatic brain injury [185] was increased (vascular system, Fig. 4, Supplementary Table 4). Thus, multiple processes likely contributed to rescue of cerebrovascular deficits in EFV-treated mice, and a separate investigation is required to identify the major contributors to the observed cerebrovascular improvements.

The limitation of this study is that it was conducted only on one mouse model; hence all the identified effects are currently conditional and need to be tested for being operative on other mouse models.

To summarize, we established the biochemical basis in 5XFAD mice for the EFV-mediated increase in brain energy production as well as synthesis of specific lipids (PC and SM) and amino acids (Ala, Gly, Pro, Ile, Tyr, and Val) that collectively contribute to repair of cellular and tissue damages in the 5XFAD brain and its vasculature, thus rescuing aberrant processes in 5XFAD mice (Fig. 8). Similar rescuing effects could be operative upon CYP46A1 activity increase in other neurologic conditions, thus explaining a broad spectrum of brain disorders benefiting from EFV treatment. The data obtained further detail and expand our chain reaction hypothesis (Fig. 1) and provide it with novel mechanistic insights, concepts, and future directions for research.

## Supplementary Material

1

## Data Availability

Data will be made available on request.

## Appendix A. Supporting information

Supplementary data associated with this article can be found in the online version at doi:10.1016/j.biopha.2026.119470.

## Abbreviations:

24HC: 24-hydroxycholesterol
AD: Alzheimer’s disease
ASR: absolute synthesis rates
BBB: blood-brain barrier
DAM: differentially abundant metabolite
DAP: differentially acetylated protein
DEP: differentially expressed protein
EFV: efavirenz
ESI: electrospray ionization
FA: fatty acids
FITC: fluorescein isothiocyanate
GC: gas chromatography
MS: mass spectrometry
PC: phosphatidylcholine
SM: sphingomyelin
TCA: tricarboxylic acid
WT: wild type
