# Supplementary material for: CYP46A1 activation by low-dose efavirenz uncovers the link between brain cholesterol metabolism, energetics, and vasculature
Source: Biomed Pharmacother. Author manuscript; Available in PMC 2026 Jun 8. (PMC13243862; doi:10.1016/j.biopha.2026.119470)
Supplement: 1 [file NIHMS2179271-supplement-1.pdf]

Supplementary Text 1. The abbreviations for DEPs (differentially expressed proteins) and **DAPs** (differentially acetylated proteins) according to the UniProt nomenclature (https://www.uniprot.org/).

AAMDC, mth938 domain-containing protein; **AARS1**, alanine-tRNA ligase, cytoplasmic; **AARSD1**, alanyl-tRNA editing protein Aarsd1; **AASS**, α-aminoadipic semialdehyde synthase, mitochondrial; **ABCB10**, ATP-binding cassette sub-family B member 10, mitochondrial; **ABHD4**, lyso-N-acyl-phosphatidylethanolamine lipase; **ACACA**, acetyl-CoA carboxylase 1; **ACADM**, medium-chain specific acyl-CoA dehydrogenase, mitochondrial; **ACLY**, ATP-citrate synthase; **ACO2**, aconitate hydratase, mitochondrial; **ACOT1**, acyl-CoA thioesterase 1; **ACOT13**, acyl-CoA thioesterase 13; **ACOT3**, acyl-CoA thioesterase 3, mitochondrial; **ACSF2**, medium-chain acyl-CoA ligase ACSF2, mitochondrial; **ACSL1**, long-chain-fatty-acid-CoA ligase 1; **ACSL6**, long-chain-fatty-acid-CoA ligase 6; **ACSS1**, acetyl-CoA synthetase 2-like, mitochondrial; **ACTB**, actin, cytoplasmic 1; **ACTC1**, actin, α- cardiac muscle 1; **ACTG1**, actin, gamma, cytoplasmic 1; **ACTR2**, actin-related protein 2; **ACY3**, N-acyl-aromatic-L-amino acid amidohydrolase (carboxylate-forming); **ADCY9**, adenylate cyclase type 9; **ADD3**, γ-adducin; **AKAP9**, A kinase anchor protein 9; **AKT1S1**, proline-rich AKT1 substrate 1; **ALDH1A1,** aldehyde dehydrogenase 1A1; **ALDH2**, aldehyde dehydrogenase, mitochondrial; **ALDH4A1**, delta-1-pyrroline-5-carboxylate dehydrogenase, mitochondria; **ALDH5A1**, succinate-semialdehyde dehydrogenase, mitochondrial; **ALDH6A1**, methylmalonate-semialdehyde/malonate-semialdehyde dehydrogenase [acylating], mitochondrial; **ALDH7A1**, α-aminoadipic semialdehyde dehydrogenase; **ALYREF**, THO complex subunit 4; **ALYREF2**, Aly/REF export factor 2; **ANAPC2**, anaphase-promoting complex subunit 2; **ANKRD46**, ankyrin repeat domain-containing protein 46; **ANKS1A**, ankyrin repeat and SAM domain-containing protein 1A; **ANXA2**, annexin A2; **ANXA2**, annexin A2; **AP2M1**, AP-2 complex subunit mu; **AP3B2**, AP-3 complex subunit β-2; **APOH**, β-2-glycoprotein 1; **ARAF**, serine/threonine-protein kinase A-Raf; **ARF3**, ADP-ribosylation factor 3; **ARHGAP26**, Rho GTPase-activating protein 26; **ARHGEF12**, Rho guanine nucleotide exchange factor 12; **ARHGEF18**, Rho guanine nucleotide exchange factor 18; **ARHGEF18**, Rho guanine nucleotide exchange factor 18; **ARPC2**, actin-related protein 2/3 complex subunit 2; **ASL**, argininosuccinate lyase; **ASPA**, aspartoacylase; **ASPHD2**, aspartate β-hydroxylase domain-containing protein 2; **ATAD3A**, ATPase family AAA domain-containing protein 3A; **ATG3**, ubiquitin-like-conjugating enzyme ATG3; **ATP1B2**, sodium/potassium-transporting ATPase subunit be; **ATP2B3**, calcium-transporting ATPase; **ATP5H**, ATP synthase peripheral stalk subunit d, mitochondrial; **ATP5MG**, ATP synthase F(0) complex subunit g, mitochondrial; **ATP5PB**, ATP synthase F(1) complex catalytic subunit β, mitochondrial; **ATP5PF**, ATP synthase peripheral stalk subunit F6, mitochondrial; **ATP6V0A1**, V-type proton ATPase 116 kDa subunit a 1; **AUH**, methylglutaconyl-CoA hydratase, mitochondrial; **B4GAT1**, β-1,4-glucuro-nyltransferase 1; **BACE1**, β-secretase 1; **BAZ1B**, tyrosine-protein kinase BAZ1B; **BCKDHA**, 2-oxo-isovalerate dehydrogenase subunit α; **BCR**, breakpoint cluster region protein; **BEGAIN**, brain-enriched guanylate kinase-associated protein; **BLMH**, bleomycin hydrolase; **BLTP3A**, bridge-like lipid transfer protein family member 3A; **BOD1L**, biorientation of chromosomes in cell division protein 1-like 1; **BPNT1**, 3'(2'),5'-bisphosphate nucleotidase 1; **C11ORF54**, ester hydrolase C11orf54 homolog; **C2CD5**, C2 domain-containing protein 5; CA2, carbonic anhydrase 2; **CACNA1B**, voltage-dependent N-type calcium channel subunit α-1B; **CAMLG**, guided entry of tail-anchored proteins factor CAMLG OS; **CAP1**, adenylyl cyclase-associated protein 1; **CAPG**, macrophage-capping protein; **CBLN4**, cerebellin-4 OS; **CCBL2**, kynurenine-oxoglutarate transaminase 3; **CCNY**, cyclin-Y; **CELF4**, CUGBP Elav-like family member 4; **CEP97**, centrosomal protein of 97 kDa; **CFAP20**, cilia- and flagella-associated protein 20; **CFAP410**, cilia- and flagella-associated protein 410; **CLASP2**, CLIP-associating protein 2; **CLDND1**, claudin domain-containing protein 1; **CLPP**, ATP-dependent Clp protease proteolytic subunit, mitochondrial; **CLU,** clusterin; **CNOT7**, CCR4-NOT transcription complex subunit 7; **CNP**, 2',3'-cyclic-

nucleotide 3'-phosphodiesterase; **CNTN3**, contactin-3; **CNTNAP5A**, contactin-associated protein like 5-1; **COA5**, cytochrome c oxidase assembly factor 5; **COASY**, bifunctional CoA synthase; **COG4**, conserved oligomeric Golgi complex subunit 4; **COL12A1**, collagen α-1(XII) chain; **COPS2**, COP9 signalosome complex subunit 2; **COQ3**, ubiquinone biosynthesis O-methyltransferase, mitochondrial; **CPNE6**, copine-6; **CREB1**, cyclic AMP-responsive element-binding protein 1; **CREBBP**, histone acetyltransferase; **CRYZL1**, quinone oxidoreductase-like protein 1; **CRYZL2**, protein Cryzl2; **CS**, citrate synthase, mitochondrial; **CSAD**, cysteine sulfinic acid decarboxylase; **CSTF1**, cleavage stimulation factor subunit 1; **CUL2**, cullin-2; **DAAM2**, disheveled-associated activator of morphogenesis 2; **DARS2**, aspartate--tRNA ligase, mitochondrial; **DBI**, acyl-CoA-binding protein; **DCAF11**, DDB1- and CUL4-associated factor 11; **DDI2**, protein DDI1 homolog 2; **DDX3X**, ATP-dependent RNA helicase DDX3X; **DECR1,** 2,4-dienoyl-CoA reductase [(3E)-enoyl-CoA-producing], mitochondrial; **DENND5B**, DENN domain-containing protein 5B; **DGKD**, diacylglycerol kinase delta; **DHX29**, ATP-dependent RNA helicase DHX29; **DIS3L2**, DIS3-like exonuclease 2; **DLAT**, dihydrolipoyllysine-residue acetyltransferase component of pyruvate dehydrogenase complex, mitochondrial; **DLD**, dihydrolipoyl dehydrogenase, mitochondrial; **DLGAP1**, disks large-associated protein 1; **DLGAP4**, disks large-associated protein 4; **DNAJC16**, DnaJ homolog subfamily C member 16; **DPP10**, inactive dipeptidyl peptidase 10; **DST**, dystonin; **DTD1**, D-aminoacyl-tRNA deacylase 1; **DUSP15**, dual specificity protein phosphatase 15; **DYNC1H1**, cytoplasmic dynein 1 heavy chain 1; **EEF2**, elongation factor 2; **ELOB**, transcription elongation factor B polypeptide 2; **EMB**, embigin; **EMC10**, ER membrane protein complex subunit 10; **ENDOD1**, endonuclease domain-containing 1 protein; **ENO1**, α-enolase; **EPB41**, protein 4.1; **EPS15**, epidermal growth factor receptor substrate 15; **ETFA**, electron transfer flavoprotein subunit α, mitochondrial; **ETFDH**, electron transfer flavoprotein-ubiquinone oxidoreductase, mitochondrial; **EXOC6**, exocyst complex component 6; **FAH**, fumarylacetoacetase; **FAM120C**, constitutive coactivator of PPAR-gamma-like protein 2; **FAM89B**, leucine repeat adapter protein 25; **FAM98B**, protein FAM98B; **FASN**, fatty acid synthase; **FAU**, 40S ribosomal protein S30; **FBXL4**, F-box/LRR-repeat protein 4; **FGGY**, FGGY carbohydrate kinase domain-containing protein; **FH**, fumarate hydratase, mitochondrial**;** **FRMD4A**, FERM domain-containing protein 4A; **FSTL1**, follistatin-related protein 1; **FXYD1**, phospholemman; **FYTTD1**, UAP56-interacting factor; **GABRA1**, gamma-aminobutyric acid receptor subunit α-1; **GAK**, cyclin-G-associated kinase; **GAP43**, neuromodulin; **GATD3**, glutamine amidotransferase-like class 1 domain-containing protein 3, mitochondrial; **GATM**, glycine amidinotransferase, mitochondrial; **GFAP**, glial fibrillary acidic protein; **GLDC**, glycine dehydrogenase (decarboxylating), mitochondrial; **GLRB**, glycine receptor subunit β; **GLS**, glutaminase kidney isoform, mitochondrial; **GLUD1**, glutamate dehydrogenase 1, mitochondrial; **GMFB**, glia maturation factor beta; **GNB1**, guanine nucleotide-binding protein G(I)/G(S)/G(T) subunit β-1; **GNB4**, guanine nucleotide-binding protein subunit β-4; **GNGT1**, guanine nucleotide-binding protein G(T) subunit gamma-T1; **GOT2**, aspartate aminotransferase, mitochondrial; **GPAM**, glycerol-3-phosphate acyltransferase 1, mitochondrial; **GPI**, glucose-6-phosphate isomerase; **GPR107**, protein GPR107; **GPT**, alanine aminotransferase 1; **GRM2**, metabotropic glutamate receptor 2; **GRM4**, metabotropic glutamate receptor 4; **GSTK1**, glutathione S-transferase kappa 1**;** **GSTO1**, glutathione S-transferase omega-1; **GSTO2**, glutathione S-transferase omega-2; **GTF2F2**, general transcription factor IIF subunit 2**;** **GTF2I**, general transcription factor II-I; **GUCY1A3**, guanylate cyclase soluble subunit α-3; **GYG1**, glycogenin-1; **H1-1**, histone H1.1; **H1-4**, histone H1.4; **H1F0**, histone H1.0; **H2BC14**, histone H2B type 1-M; **H2BC18**, histone H2B type 2-B; **H2BC4**, histone H2B type 1-C/E/G; **H2BC9**, histone H2B type 1-H; **H2BU2**, H2B.U histone 2; **H3C14**, H3 clustered histone 14; **H4C1**,histone H4; **HADHA**, trifunctional enzyme subunit α, mitochondrial**;** **HAGH**, hydroxyacylglutathione hydrolase, mitochondrial**;** **HAPLN2**, hyaluronan and proteoglycan link protein 2; **HBB-BS**, β-globin; **HDAC6**, histone deacetylase 6; **HEATR3**, HEAT repeat-containing protein 3; **HEPACAM**, hepatocyte cell adhesion molecule; **HERC1**,

HECT and RLD domain containing E3 ubiquitin protein ligase family member 1; **HIBADH**, 3-hydroxy-isobutyrate dehydrogenase, mitochondrial; **HINT1**, adenosine 5'-monophosphoramidase HINT1; **HINT2**, adenosine 5'-monophosphoramidase HINT2; **HIRA**,protein HIRA; **HK1**, hexokinase; **HMGCL**, hydroxymethylglutaryl-CoA lyase, mitochondrial; **HP**, haptoglobin; **HSD11B1**, 11-β-hydroxysteroid dehydrogenase 1; **HSPA12B**, heat shock 70 kDa protein 12B; **HSPA5**, endoplasmic reticulum chaperone BiP; **HSPA8**, heat shock cognate 71 kDa protein; **HSPA9**, stress-70 protein, mitochondrial; **IARS2**, isoleucine-tRNA ligase, mitochondrial; **IDE**, insulin-degrading enzyme; **IDH2**, isocitrate dehydrogenase [NADP], mitochondrial; **IDH3A**, isocitrate dehydrogenase [NAD] subunit, mitochondrial; **IGF2R**, cation-independent mannose-6-phosphate receptor; **IGHM**, immuno-globulin heavy constant mu; **IGKC**, immunoglobulin kappa constant; **IGKV12-46**, immunoglobulin kappa variable 12-46 (Fragment); **IL1RAPL1**, interleukin-1 receptor accessory protein-like 1; **INA**, α-inter-nexin; **INO80D**, INO80 complex subunit D; **INPP4B**, type II inositol 3,4-bisphosphate 4-phosphatase; **IQSEC3**, IQ motif and SEC7 domain-containing protein 3; **ITPR1**, inositol 1,4,5-trisphosphate-gated calcium channel I; ITSN1**,** intersectin-1; **IVD**, isovaleryl-CoA dehydrogenase, mitochondrial; **JAKMIP3**, janus kinase and microtubule-interacting protein 3; **KCNJ4**, inward rectifier potassium channel 4; **KCNT1**, potassium channel subfamily T member 1; **KCTD4**, BTB/POZ domain-containing protein KCTD4; **KIAA1671**, uncharacterized protein KIAA1671; **KMT2A**, histone-lysine N-methyltransferase 2A; **KPTN**, KICSTOR complex protein kaptin; **LGI3**, leucine-rich repeat LGI family member 3; **LGMN**, legumain; **LIMA1**, LIM domain and actin-binding protein 1; **LIN7B,** protein lin-7 homolog B; **LMAN2L**, VIP36-like protein; **LMCD1**, LIM and cysteine-rich domains protein 1**; LPCAT1,** lysophosphatidyl-choline acyltransferase 1; **LRRC49,** leucine-rich repeat-containing protein 49; **LRRC73,** leucine-rich repeat-containing protein 73; **LXN,** latexin; **MACF1**, microtubule-actin cross-linking factor 1, isoforms 1/2/3/4; **MAL2**, protein MAL2; **MAP1B**, microtubule-associated protein 1B; **MAP1S**, microtubule-associated protein 1S; **MAP2**, microtubule-associated protein 2; **MAP2K3**, dual specificity mitogen-activated protein kinase kinase 3; **MAPRE2**, microtubule-associated protein RP/EB family member 2; **MBP**, myelin basic protein; **MCCC1**, methylcrotonoyl-CoA carboxylase subunit α, mitochondrial; **MCEE**, methylmalonyl-CoA epimerase, mitochondrial; **MCRIP1**,mapk-regulated corepressor-interacting protein 1; **MCUR1**, mito-chondrial calcium uniporter regulator 1; **MDH1**, malate dehydrogenase, cytoplasmic; **MDH2**, malate dehydrogenase, mitochondrial; **ME1**, NADP-dependent malic enzyme; **MEAF6**, chromatin modification-related protein MEAF6; **MEAK7**, MTOR-associated protein MEAK7; **MECP2**, methyl-CpG-binding protein 2; **MED1**, mediator of RNA polymerase II transcription subunit 1; **MID1IP1**, Mid1-interacting protein 1; **MIF**, macrophage migration inhibitory factor; **MRPL22**, large ribosomal subunit protein uL22m; **MRPL30**, large ribosomal subunit protein uL30m; **MRPL53**, large ribosomal subunit protein mL53; **MSN**, moesin; **MTX1**, metaxin-1; **MYG1**, UPF0160 protein MYG1, mitochondrial; **MYLK**, myosin light chain kinase, smooth muscle; **MYO1D**, unconventional myosin-Id; **NACAD**, NAC-α domain-containing protein 1; NAPA**,** α-soluble NSF attachment protein; **NBAS,** neuroblastoma amplified sequence; **NCK2**, cytoplasmic protein NCK2; **NCL**, nucleolin; **NDUFA13**, NADH dehydrogenase [ubiquinone] 1 α subcomplex subunit 13; **NDUFA7**, NADH dehydrogenase [ubiquinone] 1 α sub-complex subunit 7; **NDUFB7**, NADH dehydrogenase [ubiquinone] 1 β subcomplex subunit 7; **NETO1**, neuropilin and tolloid-like protein 1; **NEUROD2**, neurogenic differentiation factor 2; **NGLY1**, peptide-N(4)-(N-acetyl-β-glucosaminyl) asparagine amidase; **NHP2**, H/ACA ribonucleoprotein complex subunit 2; **NME1**, nucleoside diphosphate kinase; **NOL3**, nucleolar protein 3; **NOLC1**, nucleolar and coiled-body phosphoprotein 1; **NRAS**, GTPase NRas; **NSDHL**, sterol-4-α-carboxylate 3-dehydrogenase, decarboxyl-ating; **NT5C1A**, cytosolic 5'-nucleotidase 1A; **NT5C3A**, cytosolic 5-nucleotidase 3A; **NT5M**, 5'(3')-deoxyribonucleotidase, mitochondrial; **NTN1,** netrin-1; **NUCB2**, nucleobindin-2; **NUDT21,** cleavage and poly-adenylation specificity factor subunit 5; **OLFM1**, noelin; **OPA1**, dynamin-like GTPase OPA1, mitochondrial; **OS9**, protein OS-9; **OXCT1**, succinyl-CoA:3-ketoacid CoA transferase 1, mitochondrial**;**

PAFAH1B3, platelet-activating factor acetylhydrolase IB subunit gamma; **PARS2**, probable proline--tRNA ligase, mitochondrial; **PC**, pyruvate carboxylase, mitochondrial; **PDIA3**, protein disulfide-isomerase A3; **PDS5B**, PDS5 cohesin associated factor B; **PEG10**, retrotransposon-derived protein PEG10; **PGAM2**, phosphoglycerate mutase 2; **PGK1**, phosphoglycerate kinase 1; **PGM1**, phospho-glucomutase-1; **PIK3CA**, phosphatidyl-inositol 4,5-bisphosphate 3-kinase catalytic subunit α isoform; **PIP4K2A**, phosphatidylinositol 5-phosphate 4-kinase type-2 α; **PKM**, pyruvate kinase PKM; **PLEK**, pleckstrin; **PLEKHA1**, pleckstrin homology domain-containing family A member 1; **PLGRKT**, plasminogen receptor (KT); **PLIN3**, perilipin-3; **PLTP**, phospholipid transfer protein; **PML**, protein PML; **POLR2E**, DNA-directed RNA polymerases I, II, and III subunit RPABC1; **PPIG**, peptidyl-prolyl cis-trans isomerase (Fragment); **PPP2R1A**, serine/threonine-protein phosphatase 2A 65 kDa regulatory subunit A α isoform; **PRDX1**, peroxiredoxin-1; **PRDX6**, peroxiredoxin-6; **PRKCB**, protein kinase C β type; **PRKCG**, protein kinase C gamma type; **PRL**, prolactin; **PRMT7**, protein arginine N-methyl-transferase 7; **PRPF39**, pre-mRNA processing factor 39 (Fragment); **PRPF40B**, pre-mRNA-processing factor 40 homolog B; **PRR36**, proline rich 36; **PSIP1**, PC4 and SFRS1-interacting protein; **PSMA6**, proteasome subunit α type-6; **PSMD7**, 26S proteasome non-ATPase regulatory subunit 7; **PTAR1**, protein prenyltransferase α subunit repeat containing **1; PTGR1**, prostaglandin reductase 1; **PTK2**, focal adhesion kinase 1; **PTMA**, prothymosin α; **PTPN5**, tyrosine-protein phosphatase non-receptor type 5; **PTPRD**, receptor-type tyrosine-protein phosphatase delta; **PTPRF**, receptor-type tyrosine-protein phosphatase F; **PTPRZ1**, receptor-type tyrosine-protein phosphatase zeta; **PVALB**, parvalbumin α; **PYCARD**, apoptosis-associated speck-like protein containing a CARD; **PYGB**, glycogen phosphorylase, brain form; **QARS**, glutamine--tRNA ligase; **RAB14**, Ras-related protein Rab-14; **RAB18**, Ras-related protein Rab-18; **RAB39B**, Ras-related protein Rab-39B; **RAB3GAP1**, Rab3 GTPase-activating protein catalytic subunit; **RABGEF1**, Rab5 GDP/GTP exchange factor; **RACK1**, small ribosomal subunit protein RACK1; **RALYL**, RNA-binding Raly-like protein; **RAP1B**, Ras-related protein Rap-1b; **RARS2**, probable arginine-tRNA ligase, mitochondrial; **RBBP5**, retinoblastoma binding protein 5, histone lysine methyltransferase complex subunit; **RBM27**, RNA-binding protein 27; **REEP3**, receptor expression-enhancing protein 3; **RELA**, transcription factor p65; **RELCH**, RAB11-binding protein RELCH; **REPS2**, RalBP1-associated Eps domain-containing protein 2; **RHOG**, Rho-related GTP-binding protein RhoG; **RNF123**, E3 ubiquitin-protein ligase RNF123; **RNF14**, E3 ubiquitin-protein ligase RNF14; **ROBO2**, roundabout homolog 2; **RPRD2**, regulation of nuclear pre-mRNA domain-containing protein 2; **RPS27A**, ubiquitin-ribosomal protein eS31 fusion protein; **RPS28**, 40S ribosomal protein S28;40S ribosomal protein S28-2; **RTN1**, reticulon-1; **RYR2**, ryanodine receptor 2, cardiac; **SDHA,** succinate dehydro-genase [ubiquinone] flavoprotein subunit, mitochondrial; **SEC11A**, signal peptidase complex catalytic subunit SEC11A; **SELO**, selenoprotein O; **SEPTIN14**, septin; **SEPTIN4**, septin-4; **SEPTIN5**, septin; **SEPTIN9**, septin-9; **SERPINA3N**, serine protease inhibitor A3N; **SFPQ**, splicing factor, proline- and glutamine-rich; **SGSM2**, small G protein signaling modulator 2; **SHF**, Src homology 2 domain containing F (Fragment); **SIK3**, serine/threonine-protein kinase SIK3**; SIRT3**, NAD-dependent protein deacetylase sirtuin-3; **SLC16A7**, monocarboxylate transporter 2; **SLC25A12**, electrogenic aspartate/glutamate antiporter SLC25A12, mitochondrial; **SLC25A3**, solute carrier family 25 member 3; **SLC44A1**, choline transporter-like protein 1; **SLC4A10**, sodium-driven chloride bicarbonate exchanger; **SLC4A4**, electrogenic sodium bicarbonate cotransporter 1; **SLC6A1**, sodium- and chloride-dependent GABA transporter 1; **SLC6A9**, solute carrier family 6 (neurotransmitter transporter, glycine), member 9; SMC3**,** structural maintenance of chromosomes protein 3; **SNAPIN**, SNARE-associated protein snapin; **SNCA**,α-synuclein; **SNTA1**, α-1-syntrophin; **SNX25**, sorting nexin-25; **SPRED1**, sprouty-related, EVH1 domain-containing protein 1; **SPRYD4**, SPRY domain-containing protein 4; **SPTAN1**, spectrin α chain, non-erythrocytic 1; **SPTBN1**, spectrin β chain, non-erythrocytic; **SPTBN2**, spectrin β chain; **SRSF10**, serine/arginine-rich splicing factor 10; **SRSF11**, serine and arginine-rich splicing factor 11;

SRSF3, serine/arginine-rich splicing factor 3; **SRSF5**, serine/arginine-rich splicing factor 5; **SSU72**, RNA polymerase II subunit A C-terminal domain phosphatase SSU72; **STIM2**, stromal interaction molecule 2; **SUGCT**, succinyl-CoA:glutarate CoA-transferase; **SUN1**, SUN domain-containing protein 1; **SUN2,** SUN domain-containing protein 2; **SYNJ2BP**, synaptojanin-2-binding protein; **SYP**, synaptophysin;**SYTL2**, Synaptotagmin-like protein 2; **TBR1**, T-box brain protein 1; **TCOF1**, treacle protein; **THEM4**, acyl-CoA thioesterase THEM4; **TIAL1**, nucleolysin TIAR; **TIMM50**, mitochondrial import inner membrane translocase subunit TIM50; **TIMM8A1**, mitochondrial import inner membrane translocase subunit Tim8 A**; TIMM8A2**, putative mitochondrial import inner membrane translocase subunit Tim8 A-B; **TKT**, transketolase; **TLCD4**, TLC domain-containing protein 4; **TMEFF2**, tomo-regulin-2; **TMEM143**, transmembrane protein 143; **TMLHE**, trimethyllysine dioxygenase, mito-chondrial; **TMPO**, lamina-associated polypeptide 2, isoforms α/zeta**; TMX4**, thioredoxin-related transmembrane protein 4; **TPM1**, tropomyosin α-1 chain; **TPPP**, tubulin polymerization-promoting protein; **TPPP3**, tubulin polymerization-promoting protein; **TRAPPC14**, trafficking protein particle complex subunit 14; **TRERF1**, transcriptional-regulating factor 1; **TRRAP**, transformation/transcription domain-associated protein; **TSC22D2**, TSC22 domain family protein 2; **TSPAN9**, tetraspanin-9; **TTYH3**, Protein tweety homolog 3; **TUBA4A**, tubulin α-4A chain; **TUBB4B**, tubulin β-4B chain; **TUBB6**, tubulin β-6 chain; **TXN**, thioredoxin; **TXNRD2**, thioredoxin reductase 2, mitochondrial; **UBE2F**, NEDD8-conjugating enzyme UBE2F; **UBE2V1**, ubiquitin-conjugating enzyme E2 variant 1; **UGP2**, UTP--glucose-1-phosphate uridylyltransferase; **UMAD1**, UMAP1-MVP12 associated (UMA) domain containing 1; **UQCR11**, cytochrome b-c1 complex subunit 10; **UQCRC1**, cytochrome b-c1 complex subunit 1, mitochondrial; **USP7**, ubiquitin carboxyl-terminal hydrolase 7; **VEGFA**, vascular permeability factor; **WDR17**, WD repeat domain 17; **WDR43**, WD repeat-containing protein 43; **YIF1B**, protein YIF1B; **YKT6**, synaptobrevin homolog YKT6; **YWHAG**, 14-3-3 protein gamma; **YWHAZ**, 14-3-3 protein zeta/delta; **ZBTB8OS**, protein archease; **ZFAND5**, AN1-type zinc finger protein 5; **ZMAT3**, zinc finger matrin-type protein 3; **ZNF148**, zinc finger protein 148.

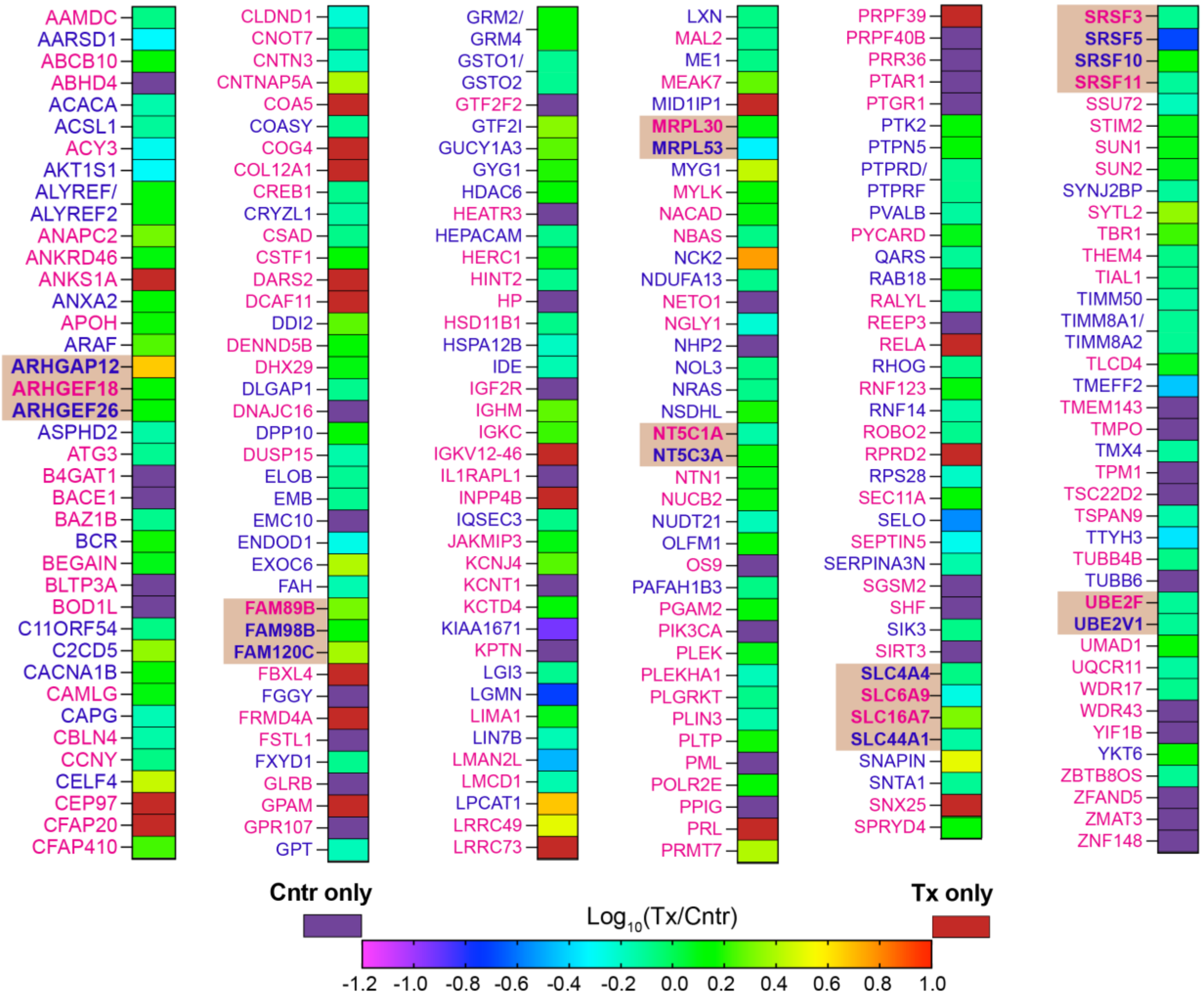

Supplementary Fig. 1. **EFV effects on brain proteome in 5XFAD mice.** DEPs from EFV-treated *vs* control female mice are in magenta and male mice are in blue (n=5 per sex and group); same protein families in both sexes are in tan boxes. See Supplementary Text 1 for the abbreviations.

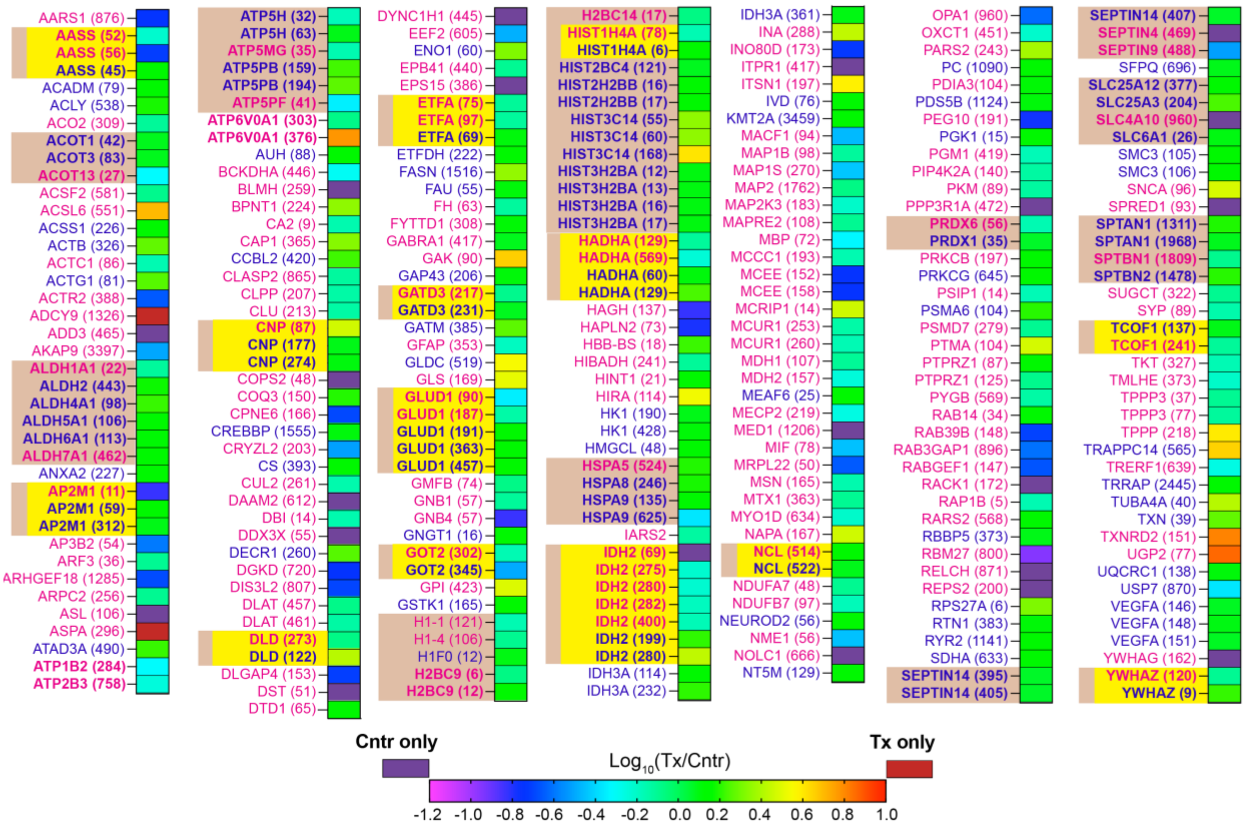

Supplementary Fig. 2. **EFV effects on brain acetylome in 5XFAD mice.** DAPs from EFV-treated *vs* control female mice are in magenta and male mice are in blue (n=5 per sex and group); same protein families in both sexes are in tan boxes, and same proteins are in yellow boxes. See Supplementary Text 1 for the abbreviations.

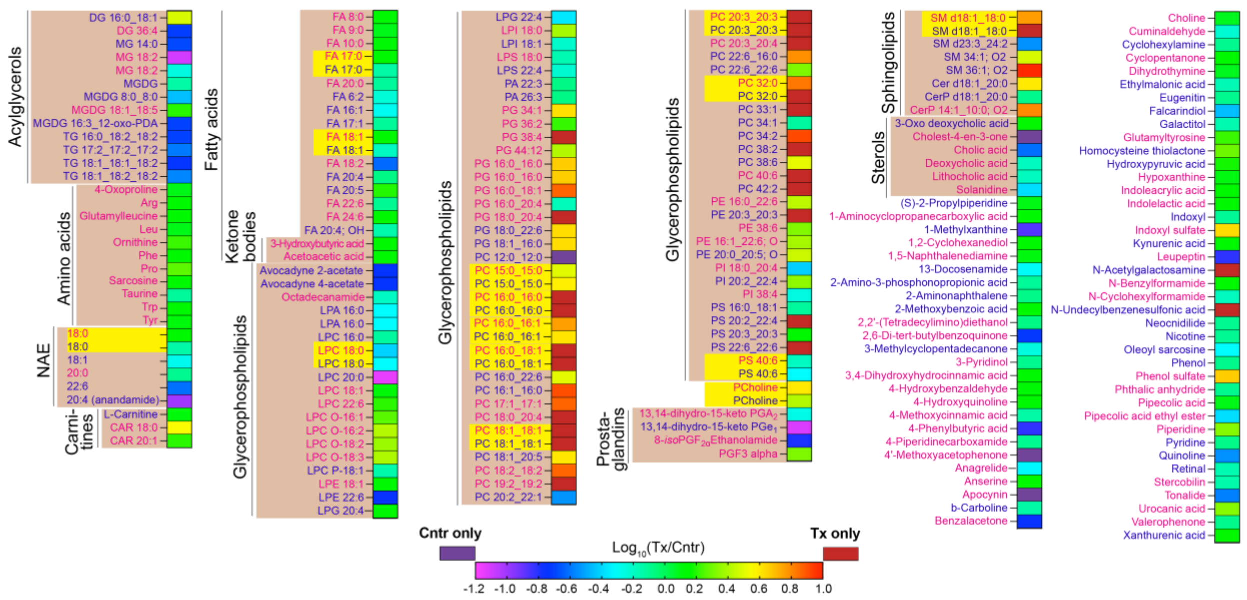

Supplementary Fig. 3. **EFV effects on brain metabolome in 5XFAD mice.** DAMs from EFV-treated *vs* control female mice are in magenta and male mice are in blue (n=5 per sex and group); same lipid classes in both sexes are in tan boxes, and same compounds are in yellow boxes. See Fig. 3 legend for the abbreviations.

Supplementary Table 1. Differentially expressed proteins (DEPs) in EFV-treated *vs* control 5XFAD female mice.

Protein IDs	Protein names	Gene names	Amino acid protein length	Peptide coverage, %	Intensity Control1	Intensity Control2	Intensity Control3	Intensity Control4	Intensity Control5	Intensity Treated1	Intensity Treated2	Intensity Treated3	Intensity Treated4	Intensity Treated5	AVE Control	AVE Treated	Fold change (Treated/Control)	P value	Total Peptide Count	Unique Peptide Count	Peptide Sequence	Modifications	M/Z	Charge
P01631	a chain V-II region 26	--	113	17.7	0.121006636	0	0.174231433	0.1999836	0.270232571	0.355981331	0.314011768	0.23609324	0.46125861	0.319827449	8.910053318	0.200085787	0.02	0.030	2	2				
		--																			FSGSGSGTDFTLK		652.3118	2
		--																			FSGVPDRFSGSGSGTDFTLK		688.0007	3
P06330	chain V region AC38 2	-----	118	57.6	0.268412649	0.284795831	0	0.37622726	0.356372398	0.465066552	0.448799041	0.66935979	0.972304505	0.577646221	28.94775632	0.296492407	0.01	0.007	4	4				
		-----																			ASGYTFTDYYMNWVK	11M(15.9949)	931.4086	2
		-----																			EVQLQQSGPELVKPGASVK		665.3687	3
		-----																			SLEWIGDINPNNGGTSYNQK		1104.0219	2
		-----																			SLTSEDSAVYYCAR	12C(57.0215)	811.3617	2
Q8R0P4	domain-containing pr	Aamdc	122	45.9	0.839838325	0.762696005	0.872154805	0.76104598	0.895283944	0.780218072	0.720794959	0.67985939	0.536089845	0.714550782	23.37071916	0.814279761	0.03	0.038	5	5				
		Aamdc																			ETGTEHSPGVQPADVK		826.3997	2
		Aamdc																			ETGTEHSPGVQPADVK		551.2689	3
		Aamdc																			EYNALVAQGVR		610.3251	2
		Aamdc																			VGGVFHSTC	9C(57.0215)	482.2213	2
		Aamdc																			VLQTEQAVK		508.2927	2
		Aamdc																			VPPSTVEYLEK		631.3373	2
Q9JI39	sub-family B member	Abcb10	9.3706	1.0	0.261712392	0.252895668	0.298090803	0.26943768	0.281878349	0.340370121	0.353436124	0.4483967	0.318067878	0.337253443	0.630856196	0.288534525	0.46	0.007	5	5				
		Abcb10																			ATQDSLAEATQLAEER		866.921	2
		Abcb10																			LPFNEGMVLDEK		696.3474	2
		Abcb10																			LQGAAATGVR		472.2696	2
		Abcb10																			LYDPNSGTVSLDGHDIR		620.3025	3
		Abcb10																			NANFVAVLDHGK		642.8383	2
Q8VD66	phosphatidylethanolar	Abhd4	342	13.2	0.322774435	0	0	0.35627658	0.356972452	0	0	0	0	0	6.740337218	0.142649806	0.02	0.000	3	3				
		Abhd4																			DVPITMIYGANTWIDTSTGKK		1156.0854	2
		Abhd4																			TLHTFDLLGFGR		459.5822	3
		Abhd4																			VAGPWGPGLVQR		618.846	2
Q91XE4	acid amidohydrolase	Acy3	318	25.5	0	0.345587153	0	0.35236989	0.364298778	0	0	0	0.276245301	0.265750792	12.73585	0.212451164	0.02	0.006	4	4				
		Acy3																			LFLYEPAGTETFSVESISK		1059.5356	2
		Acy3																			SCTLTFLGSTATPDDPYEVK	2C(57.0215)	1101.5172	2
		Acy3																			TADGDLAGTVHPQLQDHDFEPLRPGEPIFK		825.9125	4
		Acy3																			SSLPGSREPLLR	N-term(42.0106)	677.3779	2
Q8BZQ7	promoting complex su	Anapc2	837	5.1	0.260400032	0.329600714	0.391092714	0.49384259	0	0.592485382	0.72198749	0.55607912	0.359576023	0.679084955	2.698900016	0.36140428	0.13	0.043	3	3				
		Anapc2																			FGEAPMHFCEVMLK	9C(57.0215)	565.9279	3
		Anapc2																			LLQSPLCAGCGSDK	C(57.0215),7C(57.02	753.358	2
		Anapc2																			VCAEAVTTTLHQVTR	2C(57.0215)	843.4356	2
Q8BTZ5	at domain-containing	Ankrd46	228	20.2	0.545095252	0.61146018	0.632570825	0.54907938	0.558547446	0.613598437	0.668193305	0.82154546	0.812929386	0.706608471	10.36024763	0.593051254	0.06	0.014	4	4				
		Ankrd46																			GNVDICQLLHK	6C(57.0215)	432.8958	3
		Ankrd46																			LLESLEEQEVK		658.8508	2
		Ankrd46																			RLLESGFDPNIRDSR		592.3113	3
		Ankrd46																			TGLHLAAAR		455.2669	2
P59672	l SAM domain-contai	Anks1a	1150	2.3	0	0	0	0	0	0	0	0.80385814	0.6769529	0	1.13045	0	0.00	0.000	3	2				
		Anks1a																			IMSSIGEGIDFSQEQQK	2M(15.9949)	956.9514	2
		Anks1a																			NVIAEHEIR		540.7934	2
Q01339	eta-2-glycoprotein 1 C	Apoh	345	9.9	0	0.325248047	0.31200927	0.35077592	0.388265857	0.416368661	0.427373435	0.35197608	0.472194454	0.411379573	4.92755	0.358533552	0.07	0.027	3	3				
		Apoh																			ATFGCHETYK	5C(57.0215)	607.2689	2
		Apoh																			CSYTVEAHCR	(57.0215),9C(57.02	641.7688	2
		Apoh																			TSYDPGEQIVYSCK	13C(57.0215)	823.872	2
Q6P9R4	nucleotide exchange	Arhgef18	1405	5.4	0.235325345	0.35164585	0.297592845	0.36955788	0.284721519	0.359535022	0.360408092	0.49939673	0.408566148	0.364573155	2.822312672	0.332610623	0.12	0.035	6	6				
		Arhgef18																			GKSPAHLKDK		540.8116	2
		Arhgef18																			GNLLLEQER		536.2932	2
		Arhgef18																			IIQNTEAGTEDYK		741.3595	2
		Arhgef18																			RAVESCPDEEEDVFSEAEEK	6C(57.0215)	785.6691	3
		Arhgef18																			RQDVLYELMQTEAHHVR		532.0207	4
		Arhgef18																			YPVLVER		438.2529	2
Q9CPX6	ke-conjugating enzym	Atg3	314	24.2	1.167751778	1.42344236	1.50379257	1.25462034	1.196993789	1.203593771	1.116641973	0.76859212	0.917621256	1.153699448	12.68577589	1.316488567	0.10	0.042	5	5				
		Atg3																			ADAGGEDAILQTR		658.8256	2
		Atg3																			FVQAVIPTIEYDYTR		907.9697	2
		Atg3																			LWLFGYDEQRQPLTVEHMYEDISQDHVKK		721.7564	5
		Atg3																			TYDLYITYDK		647.8137	2
		Atg3																			MQNVINTVK	N-term(42.0106)	544.7921	2
Q8BWP8	,4-glucuronyltransfera	B4gat1	415	15.4	0.323891989	0	0	0	0.238236764	0	0	0	0	0	7.872795994	0.047647353	0.01	0.000	5	5				
		B4gat1																			ARYPNSPHR		366.5248	3
		B4gat1																			ISQACELHVAGFNFEVLNEGFLVH	5C(57.0215)	715.3628	4
		B4gat1																			QYGFNR		392.6905	2
		B4gat1																			SCQEVFDK	2C(57.0215)	506.7239	2
		B4gat1																			TALASGGVLDASGDYR		776.8837	2
P56818	Beta-secretase 1 OS	Bace1	501	4.2	0.22648513	0	0	0.17149427	0.177254307	0	0	0	0	0	2.209042565	0.069749715	0.03	0.000	2	2				
		Bace1																			GSFVEMVDNLR		633.8109	2
		Bace1																			GVYVPYTQGK		556.2927	2
Q9Z277	ne-protein kinase BAZ	Baz1b	1479	5.7	0.462929323	0.383173556	0.402589413	0.34625649	0.407577998	0.389136126	0.37480556	0.27425877	0.294008072	0.281390579	3.105014661	0.385746717	0.12	0.040	9	9				
		Baz1b																			EAWEEEQEVAELLK		851.9121	2
		Baz1b																			EENVLGKVDR		579.8093	2
		Baz1b																			GILNGQK		365.2163	2
		Baz1b																			RELQER		415.7276	2
		Baz1b																			RYELLEHK		363.2013	3
		Baz1b																			RYQEITHSIYLAR		550.6301	3
		Baz1b																			STGSSQLTHK		523.2672	2
		Baz1b																			TREEYEAR		527.2515	2
		Baz1b																			YWLFSNEVPGLFIEK		921.4772	2
F8WIG2	ed guanylate kinase-a	Begain	619	18.4	0.384131009	0.318979274	0.337572184	0.34010656	0.345935165	0.364593418	0.385900606	0.51845258	0.409834169	0.418411994	9.400465505	0.34143732	0.04	0.031	9	9				
		Begain																			DCNLAAQLLQCSQTYGR	C(57.0215),2C(57.02	999.462	2
		Begain																			HYLEIELRR		410.2314	3
		Begain																			LETEFDSTR		549.259	2
		Begain																			LSEDNELYRK		633.8198	2
		Begain																			MWTGGR	1M(15.9949)	362.1658	2
		Begain																			RAMSHEIVALNSHLLEAK		505.5255	4

		Begain																			SADALAGYAASDGDGDRLR		940.9402	2
		Begain																			VSLHMEK		422.2233	2
		Begain																			YYGGGGGGGAAGGGSPGDK		771.3344	2
B2KF50	(ICBP90) binding pro	Bltp3a	1429	1.5	0	0.187809257	0	0.20072322	0	0	0	0	0	0	0.7348	0.077706495	0.11	0.000	2	2				
		Bltp3a																			LDAFWLK		446.75	2
		Bltp3a																			SIEGDLSSAIHMTK		744.8717	2
E9Q6J5	nosomes in cell divisi	Bod1l	3032	1.1	0.240846786	0.343484454	0	0	0	0	0	0	0	0	0.681123393	0.068696891	0.10	0.000	3	3				
		Bod1l																			AFIATSTEGTDK		620.8063	2
		Bod1l																			EIPTDVER		479.7456	2
		Bod1l																			TGDATTTSSEVGEK		691.8177	2
P49070	il-anchored proteins f	Camlg	294	14.6	0.09855435	0.248246507	0.24550516	0.24153957	0.213020706	0.265902144	0	0.33826776	0.347431683	0.349227983	7.362227175	0.242842818	0.03	0.049	3	3				
		Camlg																			ASHHGLEQYLSR		466.5688	3
		Camlg																			LNSLSIPSVSK		572.8322	2
		Camlg																			TTVLTAALLLSGIPAEVINR		1026.6068	2
		Camlg																			TTVLTAALLLSGIPAEVINR		684.7403	3
Q8BME9	Cerebellin-4 OS	Cbln4	198	23.2	0.586974543	0.442713499	0.613654054	0.85528307	0.68795336	0.443621698	0.604647524	0.33100138	0.484048133	0.420669885	11.90963727	0.608645136	0.05	0.053	4	4				
		Cbln4																			CLVVCDSNPATDSK	(57.0215),5C(57.02	783.3504	2
		Cbln4																			EAATNGVLLYLDK		703.8799	2
		Cbln4																			GSSSSPLGISVR		573.8093	2
		Cbln4																			VAFSAVR		375.2189	2
Q8BGU5	Cyclin-Y OS	Ccny	341	16.4	0.830226674	0.673769296	0.715276098	0.6893533	0.68229976	0.661640738	0.673575735	0.47469117	0.590223004	0.589532481	8.626263337	0.684467838	0.08	0.039	4	4				
		Ccny																			ASTIFLSK		433.7527	2
		Ccny																			MLLDIFDENLHPLSK	1M(15.9949)	600.9781	3
		Ccny																			QFLELLQFNINVPSSVYAK		1105.5964	2
		Ccny																			SLFINHHPPGQTSR		530.9427	3
Q9CZ62	osomal protein of 97 k	Cep97	856	7.7	0	0	0	0	0	0	0.203601505	0	0	0.203313587	3.85515	0	0.00	0.000	4	4				
		Cep97																			EDTHSLTSLPESAGHSASR		661.3118	3
		Cep97																			LGANLPCEADVHTLILDK	7C(57.0215)	660.347	3
		Cep97																			SSVESSENSVLGNSADTVK		955.4528	2
		Cep97																			VLDGYVISQK		561.3137	2
		Cep97																						
Q8BTU1	flagella-associated pro	Cfap20	193	13.0	0	0	0	0	0	0.215571908	0	0.29517755	0	0	6.4767	0.043114382	0.01	0.000	3	3				
		Cfap20																			ASNYQSTTR		514.2438	2
		Cfap20																			LPFLVMIIK		537.3432	2
		Cfap20																			PLQIWDK		450.2529	2
Q8C6G1	lagella-associated pro	Cfap410	249	8.4	1.503518963	2.080434683	1.389239421	1.69024868	1.421007817	1.905612652	2.967905044	3.48375162	2.561085057	2.745724599	4.968609482	1.69730865	0.34	0.004	2	2				
		Cfap410																			LDNQAVTEEELTR		759.3757	2
		Cfap410																			RLSELYLR		525.3087	2
Q9CQX5	domain-containing pro	Cldnd1	253	8.7	0.369605792	0	0.300944096	0.31471765	0.287849884	0	0.276739476	0.22446841	0.244779196	0	4.532652896	0.180702326	0.04	0.033	2	2				
		Cldnd1																			IAWEDFLGDEADEK		819.3701	2
		Cldnd1																			TYNDVLFR		514.264	2
Q60809	transcription complex	Cnot7	285	10.5	0	0	0	0.34153887	0.340020422	0.282417047	0	0	0	0.287141511	5.26315	0.192795268	0.04	0.020	2	2				
		Cnot7																			GGLQEVAEQLELER		785.9071	2
		Cnot7																			YNYVAMDTEFPGVVAR		916.4378	2
		Cnot7																			YNYVAMDTEFPGVVAR		611.2943	3
Q07409	Contactin-3 OS	Cntn3	1028	3.7	0.276534229	0	0.338099849	0.29490882	0.32213959	0	0	0.40984682	0	0.39996613	1.986517115	0.191029652	0.10	0.007	3	3				
		Cntn3																			ADAGTYTCTAENQFGK	8C(57.0215)	867.3754	2
		Cntn3																			HGLIYSSAELK		609.3298	2
		Cntn3																			TPFSVGWQSVR		632.3276	2
Q0V8T9	n-associated protein lil	Cntnap5a	1304	2.1	0.435627719	0	0.39158444	0	0	0	0.640748011	0.78670113	0	0.643988902	1.25311386	0.078316888	0.06	0.010	3	3				
		Cntnap5a																			AFQGCMR	(57.0215),6M(15.99	443.1889	2
		Cntnap5a																			HASIAPVTVQK		575.8325	2
		Cntnap5a																			SDVADFDGR		491.2172	2
Q99M07	e c oxidase assembly	Coa5	74	33.8	0	0	0	0	0	0.123629463	0.072787414	0	0	0	16.8919	0.024725893	0.00	0.000	2	2				
		Coa5																			ALQYSFFECK	9C(57.0215)	646.8026	2
		Coa5																			YYEDKPEGGACAGVK	11C(57.0215)	822.3721	2
Q8R1U1	gomeric Golgi comple	Cog4	785	3.9	0	0	0	0	0	0	0	0.42868613	0.28504326	0	1.9745	0	0.00	0.000	2	2				
		Cog4																			GVTSAVNIMHSSLQQGK		878.9541	2
		Cog4																			LFSQGIGGEQAQAK		717.3728	2
Q60847	agen alpha-1(XII) chai	Col12a1	3120	1.7	0	0	0	0	0	0.538319565	0	0.33717732	0.364079393	0.585192347	0.8654	0.107663913	0.12	0.000	4	4				
		Col12a1																			HVFIVDDFESFEK		806.3881	2
		Col12a1																			NFASVQGVSLESGSFPSYSAYR		1177.0583	2
		Col12a1																			TEWQLNAHR		385.5282	3
		Col12a1																			TGPPGSTGSR		458.7278	2
Q01147	sponsive element-bind	Creb1	327	15.0	0.263080503	0.333842985	0.302219705	0.31945994	0.272303414	0.241934418	0.24268	0.24468472	0.227264625	0.241764986	7.623890252	0.293952093	0.04	0.007	3	3				
		Creb1																			DLYCHK	4C(57.0215)	418.192	2
		Creb1																			ILNDLSSDAPGVPR		727.3859	2
		Creb1																			TAPTSTIAPGVVMASSPALPTQPAEEAAR		941.1515	3
Q9DBE0	sulfinic acid decarbo	Csad	493	13.8	0.536880997	0.568707341	0.536653017	0.53862108	0.570924333	0.458582806	0.483522261	0.38604865	0.442146575	0.482452795	7.164990498	0.534697716	0.07	0.006	5	5				
		Csad																			FFNQLFSGLDPHALAGR		630.66	3
		Csad																			GAAFLGLGTDSVR		632.3381	2
		Csad																			GTMMIGYQPHGTR		724.8422	2
		Csad																			LLAAGLQCSALLLR	8C(57.0215)	749.9423	2
		Csad																			VCEWKEPEELK	2C(57.0215)	482.9026	3
Q99LC2	stimulation factor sub	Cstf1	431	16.0	0	0.227701119	0.207650066	0.24555924	0.235732836	0.244713573	0.237560225	0.32802328	0.276467988	0.262264832	8.00465	0.232271367	0.03	0.054	4	4				
		Cstf1																			AHDGAEVCSAIFSK	8C(57.0215)	746.3484	2
		Cstf1																			LGMENDDTAVQYAIGR		876.9146	2
		Cstf1																			SISFHPSGDFILVGTQHPTLR		770.406	3
		Cstf1																			TQAVFNHTEDYILLPDER		721.0235	3
Q8BIP0	-tRNA ligase, mitoch	Dars2	653	4.6	0	0	0	0	0	0.654243246	0	0	0	0.765399224	2.2971	0.130848649	0.06	0.000	3	3				
		Dars2																			EDIEFIRK		525.2849	2
		Dars2																			ILCEAPVESVVR	3C(57.0215)	686.3686	2
		Dars2																			IPEFSSFVAR		576.806	2
Q91VU6	d CUL4-associated fa	Dcaf11	549	4.2	0	0	0	0	0	0.436058834	0.516186132	0	0	0	2.0947	0.087211767	0.04	0.000	2	2				
		Dcaf11																			TALQQSFPQMLHQR		562.2911	3
		Dcaf11																			TQVGLATGR		451.7563	2
A2RSQ0	omain-containing prot	Dennd5b	1274	6.0	0	0.127426729	0.120937989	0	0	0	0	0.18190753	0	0.165378083	3.022	0.049672944	0.02	0.045	6	5				
		Dennd5b																			FLFQLHK		466.7713	2
		Dennd5b																			ILIGELMTSASDEDLGK		896.4558	2

		Dennd5b																			ILQTIQDFTIVLEGSLIK		1016.0904	2
		Dennd5b																			SALWSHLLQFQDREEK		663.0059	3
		Dennd5b																			SGSSAAPGPGSGSSPAACR	m(42.0106),18C(57.	851.8761	2
Q6PGC1	ndent RNA helicase I	Dhx29	1365	5.4	0	0.242649397	0.268921782	0.19483017	0.22167712	0.262866745	0	0.32515628	0.26405017	0.299123759	2.7106	0.238189042	0.09	0.047	7	7				
		Dhx29																			AQVHPSSVNR		547.7886	2
		Dhx29																			KLQEDGLLADVSHVIVDEVHER		626.0803	4
		Dhx29																			LACMVETAQGK	3C(57.0215)	604.2941	2
		Dhx29																			LINKLK		364.7551	2
		Dhx29																			NSLCGYQIR	4C(57.0215)	555.7716	2
		Dhx29																			SAAATEEEK		468.2194	2
		Dhx29																			SAEAGAVGEAQSK		602.7938	2
Q80TN4	olog subfamily C mem	Dnajc16	772	7.8	0.251833649	0	0.197214222	0.21493646	0	0	0	0	0	0	4.011916825	0.082430136	0.02	0.000	5	5				
		Dnajc16																			AYEILSNEEK		598.2957	2
		Dnajc16																			LLEGSLQR		458.2665	2
		Dnajc16																			QVIEDFIAQNK		652.8458	2
		Dnajc16																			QYNVNLYTPTMLIFK		922.9843	2
		Dnajc16																			TVDEETVASCDPDSSR	10C(57.0215)	884.3705	2
Q8R4V2	ficity protein phospha	Dusp15	235	18.3	0.553156635	0.586427621	0.606344617	0.63269643	0.625708166	0.522156896	0.531258677	0.48971919	0.593113495	0	9.425528317	0.594666746	0.06	0.050	5	5				
		Dusp15																			DSHQPLPLLAR		416.2349	3
		Dusp15																			ISVSDTPEVPIK		642.8558	2
		Dusp15																			ISVSDTPEVPIKK		471.6046	3
		Dusp15																			QQLEEFGWANSQK		782.8731	2
		Dusp15																			RQLEER		415.7276	2
Q9QUI1	: repeat adapter protei	Fam89b	189	15.3	0	0	0.163105404	0.16774893	0	0	0.217988885	0	0.232738844	0.209119726	7.67195	0.066170866	0.01	0.006	2	2				
		Fam89b																			HAAGSANSGSAAGPR		655.8134	2
		Fam89b																			RPVNLDSALAALRK		508.6387	3
Q8BH70	x/LRR-repeat protein	Fbxl4	621	5.3	0	0	0	0	0	0.784512262	0.663991153	0	0.904663237	0	2.657	0.156902452	0.06	0.000	2	2				
		Fbxl4																			SVCDTDIEELASNCTR	C(57.0215),3C(57.02	935.4013	2
		Fbxl4																			TALVDMNDLEDDDYEEK		1007.9252	2
Q8BIE6	omain-containing prote	Frmd4a	1020	2.9	0	0	0	0	0	0.190966931	0	0	0.223385531	0	1.4706	0.038193386	0.03	0.000	2	2				
		Frmd4a																			SIWAMAISQHQFYLDRK		698.6911	3
		Frmd4a																			SLESDQEGHYSVK		739.8415	2
Q62356	statin-related protein	Fstl1	306	16.3	2.085680356	1.727981987	0	0	0	0	0	0	0	0	9.212790178	0.345596397	0.04	0.000	3	3				
		Fstl1																			FVEQNETAINITTYADQENNK		611.2909	4
		Fstl1																			GVQTHTEEEK		579.2752	2
		Fstl1																			YFKSFDNGDSHLDSSEFLK		746.0113	3
P48168	ne receptor subunit be	Glrb	496	5.8	0	1.277387393	0	0	1.336019105	0	0	0	0	0	2.9234	0.5226813	0.18	0.000	2	2				
		Glrb																			MQLESFGYTTDDLR	1M(15.9949)	846.3826	2
		Glrb																			QYLCPSQQSPEDLAR	4C(57.0215)	896.4201	2
Q61586	hate acyltransferase 1,	Gpam	827	3.1	0	0	0	0	0	0.645030952	0.60976819	0.49280531	0	0.640838938	1.57195	0.12900619	0.08	0.000	2	2				
		Gpam																			GMFATSVTENVLSSSR		843.4118	2
		Gpam																			VDFAQPFSLK		576.3084	2
Q8BUV8	Protein GPR107 OS	Gpr107	551	5.4	0.537404029	0.469947446	0.62483085	0	0	0	0	0	0	0	2.991002014	0.218955659	0.07	0.000	4	3				
		Gpr107																			HLQEASATDGK		578.7832	2
		Gpr107																			QLPEIVFSK		530.8054	2
		Gpr107																			VHLNTFGFFK		605.3244	2
Q8R0A0	nscription factor IIF su	Gtf2f2	249	13.3	0	0	0.313502944	0.35433642	0	0	0	0	0	0	6.6265	0.133567873	0.02	0.000	3	3				
		Gtf2f2																			AECRPAASENYMK	3C(57.0215)	763.8399	2
		Gtf2f2																			PASVSAPR		392.7192	2
		Gtf2f2																			AERGELDLTGAK	N-term(42.0106)	651.3384	2
Q8BQM4	repeat-containing prote	Heatr3	679	5.6	0	0	0.19185706	0.18446325	0	0	0	0	0	0	2.79825	0.075264063	0.03	0.000	3	3				
		Heatr3																			LLAALK		314.7233	2
		Heatr3																			VLESALLCPVASMEYILLK	8C(57.0215)	1075.086	2
		Heatr3																			YSPDQLCVLDNVK	7C(57.0215)	775.8796	2
E9PZP8	ining E3 ubiquitin pro	Herc1	4859	2.6	0.267780242	0.359210737	0.390369192	0.35839023	0.349617418	0.43360678	0.350685105	0.44465674	0.391691397	0.461502237	1.440740121	0.378238871	0.26	0.049	10	9				
		Herc1																			AQCCIVENGQILTHGSGGK	(57.0215),4C(57.02	676.9912	3
		Herc1																			GYGLASTGVTSGCYQWK	13C(57.0215)	917.9251	2
		Herc1																			HSAVVTSDGK		500.7565	2
		Herc1																			KLTFEPHR		343.1943	3
		Herc1																			LGLGDSNNQSTLK		673.8491	2
		Herc1																			LVTSPNFVVTQALVALLADK		1050.1091	2
		Herc1																			SSSGFQSSTLLTSVR		778.8994	2
		Herc1																			TGLSLLFALLR		602.3766	2
		Herc1																			VVQSLLDLLCSQLK	10C(57.0215)	808.458	2
Q9D0S9	'-monophosphoramidas	Hint2	163	59.5	2.213595448	1.838783054	1.998553756	1.88295154	2.111476608	1.907776271	1.870948417	1.21310755	1.576334674	1.584577294	30.86139772	1.947908246	0.06	0.052	7	7				
		Hint2																			AAPGGASPTIFSR		616.3251	2
		Hint2																			DVAPQAPVHFLVIPR		829.9724	2
		Hint2																			DVAPQAPVHFLVIPR		553.6507	3
		Hint2																			IAQAQGLK		414.7505	2
		Hint2																			ISQAEEDDQQLLGHLLLVAK		1110.597	2
		Hint2																			ISQAEEDDQQLLGHLLLVAK		740.7338	3
		Hint2																			LVVNDGK		372.7161	2
		Hint2																			MGAQSVYHLHIHVLGGR		625.665	3
		Hint2																			MGAQSVYHLHIHVLGGR	1M(15.9949)	630.9966	3
		Hint2																			SLPADILYEDQQCLVFR	13C(57.0215)	1034.0144	2
Q61646	Haptoglobin OS	Hp	347	25.1	0.537409194	0.212303575	0	0	0	0	0	0	0	0	12.8047046	0.042460715	0.00	0.000	8	8				
		Hp																			DITPTLTLYVGK		660.8741	2
		Hp																			GSFPWQAK		460.7348	2
		Hp																			LPECEAVCGKPK	(57.0215),8C(57.02	694.339	2
		Hp																			LPECEAVCGKPK	(57.0215),8C(57.02	463.2284	3
		Hp																			LRAEGDGVYTLNDEK		840.4154	2
		Hp																			QWVNTVAGEK		566.2932	2
		Hp																			SCAVAEYGVYVR	2C(57.0215)	687.3295	2
		Hp																			VGYVSGWGR		490.7511	2
		Hp																			VMPICLPSK	5C(57.0215)	522.7826	2
P50172	droxysteroid dehydrog	Hsd11b1	292	15.1	0.416662651	0.399104516	0.337029052	0.32572429	0.36214412	0.295935747	0.300384218	0.23008965	0.347095274	0.337417065	7.742581325	0.343987546	0.04	0.052	4	4				
		Hsd11b1																			EECALEIIK	3C(57.0215)	552.7839	2
		Hsd11b1																			KSEVYYDK		516.2558	2
		Hsd11b1																			MTQPMIAPYSASK	1M(15.9949)	720.8466	2

		Hsd11b1																			SPLTPILLGNPGRK		488.2962	3
Q07113	dent mannose-6-phosp	Igf2r	2483	2.5	0	0	0.734753122	0.78655304	0	0	0	0	0	0	1.22835	0.304261232	0.25	0.000	4	4				
		Igf2r																			GNQAFDVGRPK		594.8096	2
		Igf2r																			NCPEDAAVCAVDK	'(57.0215),9C(57.02	724.8109	2
		Igf2r																			SEGGSGGNWYAMENSR		851.3497	2
		Igf2r																			YENHEGSLAETVSISNLGVAK		740.0377	3
P01872	globulin heavy constar	Ighm	454	33.9	3.219248192	1.576955586	1.803779772	2.27378926	2.209392844	3.149295379	3.42922008	4.74644595	5.850901982	3.216533034	18.5699741	2.202642568	0.12	0.008	15	15				
		Ighm																			DGFSGPAPR		452.2196	2
		Ighm																			EFVCTVTHR	4C(57.0215)	574.7795	2
		Ighm																			EFVCTVTHR	4C(57.0215)	383.5221	3
		Ighm																			ESATVTCLVK	7C(57.0215)	554.2893	2
		Ighm																			FISKPNEVHK		400.2241	3
		Ighm																			GFSPADISVQWLQR		802.4149	2
		Ighm																			GVASVCVEDWNNR	6C(57.0215)	753.3437	2
		Ighm																			GVASVCVEDWNNRK	6C(57.0215)	817.3912	2
		Ighm																			GVASVCVEDWNNRK	6C(57.0215)	545.2632	3
		Ighm																			HPPAVYLLPPAR		665.8851	2
		Ighm																			IHYGGK		337.6846	2
		Ighm																			LICEATNFTPK	3C(57.0215)	647.329	2
		Ighm																			LVESGFTTDPVTIENK		875.4489	2
		Ighm																			NLVAMGCLAR	7C(57.0215)	552.7862	2
		Ighm																			PITVSWLK		472.2842	2
		Ighm																			SILEGSDEYLVCK	12C(57.0215)	756.8661	2
		Ighm																			YLATSQVLLSPK		660.382	2
P01837	oglobulin kappa const	Igkc	107	46.7	1.109932968	2.163966861	2.427088122	2.81613186	2.818186112	3.965552812	3.28722211	3.28138521	4.841361216	3.254308006	23.91946648	2.838185153	0.12	0.033	5	5				
		Igkc																			DEYERHNSYTCEATHK	11C(57.0215)	680.6219	3
		Igkc																			DEYERHNSYTCEATHK	11C(57.0215)	510.7183	4
		Igkc																			DSTYSMSSTLTLTK		767.8688	2
		Igkc																			DSTYSMSSTLTLTK	6M(15.9949)	775.8663	2
		Igkc																			HNSYTCEATHK	6C(57.0215)	674.2909	2
		Igkc																			HNSYTCEATHK	6C(57.0215)	449.863	3
		Igkc																			IDGSER		338.6666	2
		Igkc																			QNGVLNSWTDQDSK		796.3709	2
A0A140T8P6	ı kappa variable 12-46	Igkv12-46	115	24.3	0	0	0	0	0	0	0.326503338	0.32639293	0	0	12.1739	0	0.00	0.000	2	2				
		Igkv12-46																			ASENIYSNLAWYQQK		907.939	2
		Igkv12-46																			FSGSGSGTQYSLK		659.8173	2
P59823	receptor accessory pro	Il1rapl1	695	5.9	0.310078343	0.293388043	0.245822234	0.28849425	0	0	0	0	0	0	3.104689172	0.165540906	0.05	0.000	3	3				
		Il1rapl1																			AFFGYSGDVSPLIYWMK		990.9818	2
		Il1rapl1																			GIMNYQEVEALK		697.8528	2
		Il1rapl1																			STFHNTYHSQMR		754.8386	2
Q6P1Y8	l 3,4-bisphosphate 4-p	Inpp4b	924	3.9	0	0	0	0	0	0.250020416	0	0	0	0.3407685	1.94805	0.050004083	0.03	0.000	2	2				
		Inpp4b																			DALYDVITVGAPAAHFQGFK		1060.5441	2
		Inpp4b																			IREQMSESILSFHIPK		639.0083	3
Q5DTN8	l microtubule-interact	Jakmip3	844	7.7	0.374412739	0.367202463	0.370140032	0.42800953	0.338958893	0.453447667	0.400599807	0.40797266	0.540687858	0.528470535	4.03790637	0.391551716	0.10	0.026	6	5				
		Jakmip3																			ALAEQGQDMK		545.7634	2
		Jakmip3																			DQNELLEFR		582.2882	2
		Jakmip3																			KMVDLESEK	2M(15.9949)	547.7734	2
		Jakmip3																			NALLSEERNELLK		764.9201	2
		Jakmip3																			SVYHLHQEEITR		504.5915	3
P52189	ectifier potassium char	Kcnj4	445	6.1	0	0.883190382	0.669332994	0.6974885	1.01377653	1.000432592	1.728735578	0.96570229	0.998066009	1.137659382	3.0337	0.852844199	0.28	0.050	3	3				
		Kcnj4																			MQAATLPLDNISYR		796.9086	2
		Kcnj4																			SHLVEAHVR		524.2883	2
		Kcnj4																			SHLVEAHVR		349.8613	3
		Kcnj4																			TYEVAGTPCCSAR	C(57.0215),9C(57.02	736.3189	2
Q6ZPR4	channel subfamily T m	Kcnt1	1224	4.0	0	0.196543993	0.176158206	0.17896163	0	0	0	0	0	0	2.00165	0.110332766	0.06	0.000	3	3				
		Kcnt1																			LFPSLSITTELTHPSNMR		1022.5302	2
		Kcnt1																			LSSCNPETRDETQL	4C(57.0215)	825.3754	2
		Kcnt1																			VFSISMLDTLLYQSFVK		996.0315	2
Q9D7X1	main-containing prote	Kctd4	259	19.3	0.288120138	0.275851397	0.259517098	0.30759535	0.30278537	0.345504983	0.35408991	0.39278838	0.354270399	0.365966178	9.796560069	0.29825084	0.03	0.000	4	4				
		Kctd4																			EDNTFVCTLETLK	7C(57.0215)	785.3768	2
		Kctd4																			ETTFLEITDNHDR		795.8733	2
		Kctd4																			GSIVHSDALHFIK		475.2612	3
		Kctd4																			HNSVEDAEQGK		607.2758	2
Q8VCX6	DR complex protein ka	Kptn	430	10.5	0	0.193622309	0	0.25004299	0	0	0	0	0	0	5.23255	0.088733059	0.02	0.000	2	2				
		Kptn																			FSSQSNVYGLAGGADGRGELLAATLK		861.4451	3
		Kptn																			GEAAVAEGPCPLLEDSFTR	m(42.0106),10C(57.	1030.9833	2
Q9ERG0	in and actin-binding p	Lima1	753	9.2	1.762239602	2.198180945	1.715440647	1.86440803	2.039654051	1.960879659	2.284423841	2.35042113	2.898434644	2.120882519	5.462769801	1.955712666	0.36	0.051	5	5				
		Lima1																			IAWPPPAELGGSGSALEEGIK		1040.0415	2
		Lima1																			LLANQQVFHISCFR	12C(57.0215)	866.9512	2
		Lima1																			NNPESLPQHFR		669.8311	2
		Lima1																			SEAQQPMHPK		576.7769	2
		Lima1																			SQDVGFWEGEVVR		754.3624	2
P59481	VIP36-like protein OS	Lman2l	347	11.2	0	0.335600686	0.438819247	0.34986291	0.304811731	0	0	0.2257484	0.253103499	0	5.6196	0.285818915	0.05	0.017	2	2				
		Lman2l																			FVGLGVFVDTYPNEEK		907.4539	2
		Lman2l																			GYYFGTSSITGDLSDNHDVISLK		830.3992	3
Q8VEE1	ysteine-rich domains p	Lmcd1	365	11.5	0.327757491	0.409132054	0.353373918	0.35736069	0.353474731	0.297322851	0.310472782	0.2896815	0.353336227	0	5.917278746	0.354132849	0.06	0.045	3	3				
		Lmcd1																			HFICEGCEQLLSGR	'(57.0215),7C(57.02	853.3928	2
		Lmcd1																			SEALGVGEVALPGQGGLPK		889.9859	2
		Lmcd1																			VEDLAWHRK		385.2086	3
Q91YK0	h repeat-containing pr	Lrrc49	686	5.7	0	0.136517702	0.136826802	0	0	0.262118568	0.194638112	0.16641399	0	0.251525404	2.84255	0.107092614	0.04	0.025	3	3				
		Lrrc49																			LTVCPIIDGEEHLR	4C(57.0215)	551.2872	3
		Lrrc49																			NLDVLDLHGNQITK		790.4255	2
		Lrrc49																			VANIASTQDRK		601.828	2
B2RWC4	Gm88 protein OS	Lrrc73	316	14.6	0	0	0	0	0	0.504913803	0.553427295	0.65668635	0	0.513559643	7.2785	0.100982761	0.01	0.000	3	3				
		Lrrc73																			GREPAAHQR		341.181	3
		Lrrc73																			LAIAVAHSSQVR		626.362	2
		Lrrc73																			VLNLDYNPLGDHVAGMLAVAVASSR		861.4513	3
Q8BI08	Protein MAL2 OS	Mal2	175	17.7	5.329587052	4.376577075	4.818169213	5.2209197	4.809820756	4.414399781	3.880632824	3.19190318	4.325081765	3.865731229	11.52194353	4.727977304	0.41	0.013	2	2				
		Mal2																			VTLPAGPDILR		576.3427	2

		Mal2																			SAGGAVPPPPNPAVSFPAPR	N-term(42.0106)	964.5048	2
Q8K0P3	associated protein ME	Meak7	455	10.8	0	3.279376983	0	3.401668	3.403093051	3.704369969	3.65201406	4.15412073	3.580921899	3.657984859	5.3846	2.757701601	0.51	0.012	4	4				
		Meak7																			CFLFSIAPR	1C(57.0215)	555.7918	2
		Meak7																			EALPPAMVTRLYNGMQR	M(15.9949),7M(15.9	660.3327	3
		Meak7																			EQFTAFLSQLLR		726.8958	2
		Meak7																			FLPEEQAEVDK		652.822	2
Q9D7N6	somal subunit protein	Mrpl30	160	25.0	0.186621506	0.256648267	0.233512331	0.22569917	0.20021259	0.282469515	0.249064111	0.31849891	0.288745611	0.22999678	12.59331075	0.239708375	0.02	0.028	3	3				
		Mrpl30																			STGELVVQWHLKPVEQEAK		726.7233	3
		Mrpl30																			VFQPKPEDHEK		451.8981	3
		Mrpl30																			YGGDPQNPHK		556.7596	2
Q6PDN3	t chain kinase, smootl	Mylk	1941	4.1	0.604257539	0.922781917	0.666460604	0.85932443	0.860127602	0.836870428	1.186035416	0.98742383	1.148770563	0.909031759	2.36292877	0.829112997	0.35	0.040	7	6				
		Mylk																			AGESPTPAGDAQAIGALKPVGNAK		740.7254	3
		Mylk																			DNIRQEISIMNCLHHPK	12C(57.0215)	527.0149	4
		Mylk																			QIQESEHIK		556.2907	2
		Mylk																			RPESQGSAPVFK		651.8436	2
		Mylk																			TISEEERK		496.2564	2
		Mylk																			VHSPQQVDFR		606.8096	2
Q5SWP3	ı domain-containing p	Nacad	604	8.6	0.270464162	0.324679114	0.328105271	0.30276945	0.32444194	0.331135994	0.405667727	0.41939201	0.390548028	0.356277177	4.439882081	0.322226355	0.07	0.007	4	4				
		Nacad																			IVMGEETCQVIASPR	8C(57.0215)	845.4185	2
		Nacad																			NILFVIAKPDVFK		752.4503	2
		Nacad																			QGACLEAHDGVK	4C(57.0215)	642.8037	2
		Nacad																			SATSQGAVETCR	11C(57.0215)	633.7908	2
E9Q411	astoma amplified sequ	Nbas	2356	3.7	0.449727923	0.465539065	0.490329943	0.48600242	0.47994095	0.463328862	0.378135568	0.35262165	0.418972593	0.350048979	2.092413962	0.477028248	0.23	0.020	6	6				
		Nbas																			LATYEEILGAPHASEQR		942.9761	2
		Nbas																			LLDFTQAQLLQGLRK		582.0087	3
		Nbas																			QGLYLVTEMER		669.8397	2
		Nbas																			QSPGAANELLR		578.3094	2
		Nbas																			SAVNSASIHNYLSK		745.8835	2
		Nbas																			VNFEIHPEGENISASPLPVK		726.3793	3
Q8R4I7	in and tolloid-like pro	Neto1	533	7.7	0	0.829169969	0	0.91293703	0	0	0	0	0	0	3.84615	0.3484214	0.09	0.000	2	2				
		Neto1																			ECVYIIEAAPRQCIELYFDEKYSIEPSW	15),2C(57.0215),30	982.2085	4
		Neto1																			HESEYNTTRV		618.2861	2
Q9JI78	yl-beta-glucosaminyl)a	Ngly1	651	7.8	1.182916373	1.274146293	1.228387647	0.92795287	1.123468658	1.072357037	0	0.63068734	0.843995618	0.813978142	4.508508187	1.125262501	0.25	0.050	4	4				
		Ngly1																			ALTCIPVSELK	4C(57.0215)	615.8418	2
		Ngly1																			QLSLSESR		460.2458	2
		Ngly1																			TSSQSFESGSVR		636.2967	2
		Ngly1																			VLQSNIQHVQLYENPVLQEK		793.7604	3
A3KFX0	solic 5'-nucleotidase 1	Nt5c1a	365	11.5	0.381973237	0.397305483	0.384100364	0.38415605	0.441216191	0.379976274	0.371130241	0.34126939	0.333594028	0	5.944386618	0.397350872	0.07	0.037	3	3				
		Nt5c1a																			IYTEQGVEEYVR		743.3646	2
		Nt5c1a																			LINSINHYDLFIER		582.9772	3
		Nt5c1a																			VAFDGDAVLFSDESER		878.9048	2
O09118	Netrin-1 OS	Ntn1	604	8.6	0.142884804	0	0.17581707	0.16127542	0.141662636	0.160593995	0.191339731	0.21173944	0	0.19204483	4.376092402	0.127869824	0.03	0.046	4	4				
		Ntn1																			ACDCHPVGAAGK	(57.0215),4C(57.02	621.7713	2
		Ntn1																			EANECVACNCNLHAR	215),5C(57.0215),8C	909.3774	2
		Ntn1																			EANECVACNCNLHAR	215),5C(57.0215),8C	606.5873	3
		Ntn1																			TWVPFQFYSTQCRK	12C(57.0215)	616.6347	3
P81117	Nucleobindin-2 OS	Nucb2	420	13.3	0.218793804	0.224115922	0.215121397	0.22800244	0.195936664	0.208101526	0.312904161	0.30205896	0.264714203	0.265055807	6.776046902	0.214255591	0.03	0.037	6	5				
		Nucb2																			IEPPDTGLYYDEYLK		908.4379	2
		Nucb2																			LSQELDLVSHK		634.8458	2
		Nucb2																			LSQELDLVSHK		423.5663	3
		Nucb2																			QEYHQAVQHLEQK		546.6058	3
		Nucb2																			RQEVGR		372.7092	2
		Nucb2																			VHNTEPVENAR		633.3153	2
Q8K2C7	Protein OS-9 OS	Os9	672	3.6	0	0	0	0.18667542	0.153841155	0	0	0	0	0	1.7857	0.068103314	0.04	0.000	2	2				
		Os9																			IVRPGAEGKEEDTR		519.6057	3
		Os9																			TTEEVQDTDR		597.2677	2
O70250	sphoglycerate mutase	Pgam2	253	18.6	0.659485411	0.731330693	0.665489244	0.67986394	0.752546142	0.732644023	0.762504091	0.87795995	0.897865904	1.135247611	9.618292706	0.712374808	0.07	0.042	8	4				
		Pgam2																			FLGDEETVRK		597.3116	2
		Pgam2																			FLGDEETVRK		398.5435	3
		Pgam2																			HNYYTSISK		556.7722	2
		Pgam2																			SFDTPPPPMDEK		680.8081	2
		Pgam2																			YAGLKPEELPTCESLK	12C(57.0215)	612.3133	3
P42337	ıosphate 3-kinase cata	Pik3ca	1068	2.7	0	0	0	2.13727612	2.034878643	0	0	0	0	0	1.3577	0.834430953	0.61	0.000	3	3				
		Pik3ca																			DNELRENDK		566.765	2
		Pik3ca																			LCLSICSVK	(57.0215),6C(57.02	540.283	2
		Pik3ca																			TEQEALEYFTK		679.8273	2
Q9JHK5	Pleckstrin OS	Plek	350	21.1	0.52824078	0.486795518	0.656549742	0.59639877	0.467823296	0.624773991	0.623291219	0.82676995	0.610377137	0.64122795	10.83557039	0.566468263	0.05	0.049	5	5				
		Plek																			FILREDPAYLHYYDPAGGEDPLGAVHLR		796.9017	4
		Plek																			FILREDPAYLHYYDPAGGEDPLGAVHLR		637.7228	5
		Plek																			GCVVTSVESSHDVK	2C(57.0215)	752.359	2
		Plek																			GSTLTSPCQDFGK	8C(57.0215)	699.3219	2
		Plek																			GSVFNTWK		469.7402	2
		Plek																			KAIKCIEGGQK	5C(57.0215)	616.345	2
Q8BUL6	domain-containing fa	Plekha1	383	7.8	0	0.369864773	0.459032619	0.34029721	0.431456406	0.496190118	0.562033559	0	0	0	3.91645	0.419368225	0.11	0.048	2	2				
		Plekha1																			QSDSQPASDSLSR		689.3156	2
		Plekha1																			TFYVQADSPEEMHSWIK		689.9875	3
		Plekha1																			NLVLTNGPALYPAATTSFSQK		1097.081	2
Q9D3P8	minogen receptor (KT	Plgrkt	147	27.9	4.639135353	4.139701954	4.913869943	4.61739467	4.42724709	4.387935291	3.880248476	3.65423938	3.551411854	3.166545965	16.26516768	4.49722979	0.28	0.015	4	4				
		Plgrkt																			GLITFESLEK		568.8135	2
		Plgrkt																			HLTMQNEMR		580.2709	2
		Plgrkt																			HLTMQNEMR	4M(15.9949)	588.2684	2
		Plgrkt																			NQQEFMVTHAR		680.8249	2
		Plgrkt																			NQQEFMVTHAR		454.219	3
		Plgrkt																			SEAEDILETEK		632.3011	2
P55065	pholipid transfer prote	Pltp	493	10.3	0	0.690097304	0.856083653	0.76062937	0.693719443	0.776868552	0.870612931	1.03074747	1.049993089	0.831968351	5.1724	0.755479664	0.15	0.043	4	4				
		Pltp																			EDNWSLPNR		565.7648	2
		Pltp																			EVVTNHAGFVTVGADLHFAK		704.7005	3
		Pltp																			MIMEGRLSAK	3M(15.9949)	576.2991	2
		Pltp																			TGLQLSQDSSGR		624.8125	2

Q60953	Protein PML OS	Pml	885	2.9	0.231069766	0	0.297674092	0	0	0	0	0	0	0	1.584484883	0.059534818	0.04	0.000	2	2				
		Pml																			LATSSPEQSWPSTFK		833.4095	2
		Pml																			QIVDAQAACTR	9C(57.0215)	616.8062	2
Q80UW8	olymerases I, II, and II	Polr2e	210	17.1	0.262361447	0.278827803	0.269307558	0.30730811	0.274439964	0.389752599	0.372026351	0.31040981	0.406871597	0.368011382	8.702630723	0.303927206	0.03	0.002	3	3				
		Polr2e																			ALIVVQQGMTPSAK		721.9053	2
		Polr2e																			AQFGDKPSEGRPR		482.2479	3
		Polr2e																			TIMQLCHDR	6C(57.0215)	587.2788	2
A2AR01	/l cis-trans isomerase (	Ppig	127	37.0	0.229649365	0	0.275031438	0.36555158	0.394244145	0	0	0	0	0	18.61877468	0.206965432	0.01	0.000	4	4				
		Ppig																			CFFDIAINNQPAGR	1C(57.0215)	811.8908	2
		Ppig																			DFMVQGGDFSEGNGR		808.3439	2
		Ppig																			GIKVQR		350.7269	2
		Ppig																			GKDTNGSQFFIT		657.8198	2
P06879	Prolactin OS	Prl	226	39.4	0	0	0	0	0	3.022847552	36.60291856	0	0	0	19.69025	0.60456951	0.03	0.000	8	8				
		Prl																			CQIAHQNNC	'(57.0215),9C(57.02	572.7348	2
		Prl																			EIEEQNK		445.2167	2
		Prl																			GNGIYFVWSQLPSLQGVDEESK		1227.1028	2
		Prl																			IISQAYPEAK		560.3058	2
		Prl																			QLLEGVEK		458.2609	2
		Prl																			QYVQDREFMVK		481.5747	3
		Prl																			QYVQDREFMVK	9M(15.9949)	729.8558	2
		Prl																			QYVQDREFMVK	9M(15.9949)	486.9063	3
		Prl																			VDNFLK		368.2054	2
		Prl																			VINDCPTSSLATPEDK	5C(57.0215)	873.9143	2
Q922X9	ginine N-methyltransf	Prmt7	692	4.8	0	0	0.157612866	0	0.164649919	0	0.196684953	0.17991006	0.222107723	0.219898005	2.3844	0.064452557	0.03	0.014	3	3				
		Prmt7																			FGEINDQDR		547.249	2
		Prmt7																			HLVQEDCEAVPHR	7C(57.0215)	530.5878	3
		Prmt7																			QVSSSAACHSR	8C(57.0215)	595.2725	2
A0A1Y7VM45	processing factor 39 (F	Prpf39	208	14.4	0	0	0	0	0	0.200715366	0	0.18828303	0	0	7.21155	0.040143073	0.01	0.000	2	2				
		Prpf39																			ETLDPGDQETNTTIR		845.3999	2
		Prpf39																			ILGIPTQLYSHHFQR		603.9968	3
Q80W14	processing factor 40 ho	Prpf40b	870	3.2	0	0	0.277375723	0	0.265040646	0	0	0	0	0	1.6092	0.108483274	0.07	0.000	2	2				
		Prpf40b																			ALEREEEEERER		525.585	3
		Prpf40b																			FVCDSAFEQITLESER	3C(57.0215)	965.9462	2
E9PV26	Proline rich 36 OS	Prr36	1098	3.1	0.157154747	0.18639298	0	0	0	0	0	0	0	0	1.626827373	0.037278596	0.02	0.000	2	2				
		Prr36																			ASSGIARPGPVVQK		683.8936	2
		Prr36																			LGELPLGALQASVVQHLLSR		701.0757	3
A0A494B9V8	erase alpha subunit rep	Ptar1	424	8.3	0	0.385413067	1.166477006	0	0.943105951	0	0	0	0	0	4.12735	0.498999205	0.12	0.000	3	3				
		Ptar1																			FIDQVLSTCR	9C(57.0215)	619.8135	2
		Ptar1																			GSLGAVPAER		478.7616	2
		Ptar1																			RTPAPDSLGLEMEHR		570.286	3
Q91YR9	staglandin reductase 1	Ptgr1	329	10.0	0.249026617	0.404128729	0.271536418	0	0	0	0	0	0	0	5.139713309	0.135133029	0.03	0.000	2	2				
		Ptgr1																			HFEGFPTDGNFELK		819.3833	2
		Ptgr1																			TGPCPQGPAPEVVIYQQLR	4C(57.0215)	1055.5411	2
Q9EPB4	speck-like protein co	Pycard	193	23.3	0.25429586	0.279087229	0.314000875	0.34069346	0.250199085	0.356648412	0.396606489	0.34780359	0.331799337	0.320979172	11.78519793	0.308125813	0.03	0.025	4	4				
		Pycard																			DAILDALENLSGDELK		858.4385	2
		Pycard																			DAILDALENLSGDELKK		922.486	2
		Pycard																			EESGAVAAAASVPAQSTAR		886.9422	2
		Pycard																			TGHFVDQHR		366.1809	3
Q8BTF8	binding Raly-like prot	Ralyl	293	23.2	0.645414172	0.648370513	0.601890473	0.50849507	0.602902841	0.551649133	0.42205094	0.50431649	0.423374536	0.521426271	11.92680709	0.582661607	0.05	0.016	7	6				
		Ralyl																			AAVAGENAR		429.725	2
		Ralyl																			KVDIEAIFSK		575.3293	2
		Ralyl																			SDELQTIKK		531.2954	2
		Ralyl																			STVGGSSSSGSK		520.7463	2
		Ralyl																			VFIGNLNTAIVK		644.8848	2
		Ralyl																			VIAGQPLDINMAGEPK	11M(15.9949)	834.9348	2
Q99KK1	xpression-enhancing p	Reep3	254	9.8	0	0	0.400657769	0.35025511	0.330433402	0	0	0	0	0	4.92125	0.216269256	0.04	0.000	2	2				
		Reep3																			EREIDDYIVQAK		739.8779	2
		Reep3																			QATESPAYGIPLK		687.8668	2
Q04207	anscription factor p65	Rela	549	6.9	0	0	0	0	0	0.33260112	0.308531204	0	0	0	3.46085	0.066520224	0.02	0.000	3	3				
		Rela																			GSFSQADVHR		552.265	2
		Rela																			INGYTGPGTVR		567.7987	2
		Rela																			NSGSCLGGDEIFLLCDK	C(57.0215),5C(57.02	942.927	2
Q5XPI3	iitin-protein ligase RN	Rnf123	1314	3.6	0.211334069	0.211013877	0	0.2316856	0.238195241	0.323102661	0	0.26366153	0.273717421	0.263833906	1.894117034	0.200799476	0.11	0.010	4	4				
		Rnf123																			ACINQHLMNNK	2C(57.0215)	448.2167	3
		Rnf123																			FLLSNVLFDMLR		734.405	2
		Rnf123																			PCPSTLLQQHMADLLR	2C(57.0215)	627.322	3
		Rnf123																			RLLSTMEK		489.276	2
Q7TPD3	oundabout homolog 2 (	Robo2	1470	3.1	0.184897743	0.175049236	0.228469588	0.21756007	0.209997925	0.269682131	0	0.28479462	0.25934106	0	1.657098871	0.22015179	0.13	0.002	3	2				
		Robo2																			AEGRPTPTIEWYK		516.5999	3
		Robo2																			ALDQTPGSSMDNLDSSVTGK		1011.9678	2
		Robo2																			DGERVETDKDDPR		511.2376	3
Q6NXI6	pre-mRNA domain-co	Rprd2	1469	1.7	0	0	0	0	0	0.208327782	0.203319409	0	0	0	0.8509	0.041665556	0.05	0.000	2	2				
		Rprd2																			VDVCSTETLK	4C(57.0215)	576.2843	2
		Rprd2																			VSSSCLDLPDSTEEK	5C(57.0215)	833.8774	2
Q9R0P6	complex catalytic sub	Sec11a	13.9665	1.0	0	0.243782752	0.225089858	0.2327053	0.24393527	0.28317595	0.288302812	0.24305862	0.24572387	0.316501759	0.49995	0.245737826	0.49	0.039	2	2				
		Sec11a																			GDNNAVDDRGLYK		718.8418	2
		Sec11a																			MLSLDFLDDVRR	1M(15.9949)	499.2572	3
A0A338P769	Septin OS	Septin5	378	5.0	4.108337215	2.800945935	3.613959211	3.26345748	2.056522597	2.179092502	2.022183744	0	1.522360154	2.483761307	4.567418607	2.782795544	0.61	0.032	34	2				
		Septin5																			LVEQLLSPR		527.8164	2
		Septin5																			MDSLAAPQDR	N-term(42.0106)	573.2664	2
Q80U12	rotein signaling modu	Sgsm2	1005	3.4	0	0	0.242021736	0.38044977	0.321012991	0	0	0	0	0	1.69155	0.188696899	0.11	0.000	3	3				
		Sgsm2																			EMEQVDTAVAAR		660.3166	2
		Sgsm2																			EYVESLHQNSR		681.3258	2
		Sgsm2																			VQELQQQAEGR		643.3283	2
A2AQ87	2 domain containing F	Shf	446	4.5	0	0.244631803	0.20327713	0.12373098	0.193716219	0	0	0	0	0	2.24215	0.153071227	0.07	0.000	2	2				
		Shf																			GAGGAGTGPGGGGSGGVAK		686.3341	2
		Shf																			RGAGGAGTGPGGGGSGGVAK		509.9256	3
Q8R104	ent protein deacetylas	Sirt3	334	7.5	0.4987995	0	0.414512993	0	0	0	0	0	0	0	3.99189975	0.082902599	0.02	0.000	2	2				
		Sirt3																			DVVQLGDVVHGVER		761.4046	2

		Sirt3																			LYTQNIDGLER		661.3409	2
O70451	carboxylate transporte	Slc16a7	484	3.7	0.214012259	0	0.22825913	0.27864437	0.204002882	0.333660251	0.345261524	0.38135383	0.486565486	0.353618615	1.96650613	0.208913326	0.11	0.001	2	2				
		Slc16a7																			PVGPSPNTK		448.7454	2
		Slc16a7																			SKQDEVTVK		517.2798	2
E9PWC4	urotransmitter transpor	Slc6a9	126	11.1	0.258134525	0.24913114	0.262698075	0.23058356	0.270339138	0.232208608	0.230267267	0	0.230007174	0	5.684617263	0.248992104	0.04	0.025	3	2				
		Slc6a9																			GMLNGAVPSEATK		637.824	2
		Slc6a9																			GMLNGAVPSEATKK		701.8715	2
Q3ZT31	Sorting nexin-25 OS	Snx25	840	3.1	0	0	0	0	0	0.272608611	0.296624224	0	0	0.235405235	1.5476	0.054521722	0.04	0.000	2	2				
		Snx25																			ILQFEDIMTNPFYR		893.9452	2
		Snx25																			LINSNSDVDFLK		682.8564	2
Q91WK1	omain-containing prot	Spryd4	207	26.1	0	0.361763015	0.409446067	0.35645838	0.341294912	0.438471361	0.377503924	0.46216623	0.45877514	0.408123878	13.0435	0.381486746	0.03	0.026	4	4				
		Spryd4																			HYWEVTVK		531.2743	2
		Spryd4																			ISVIHTLQTDFR		477.2648	3
		Spryd4																			ISVIHTLQTDFRGPVAPAFALWDGELLTHSGLEVPK		979.5255	4
		Spryd4																			TAHSSLALFR		368.2049	3
E9Q6E5	arginine-rich splicing f	Srsf11	511	11.7	0.854786787	0.814749436	0.814542619	0.72466492	0.867029212	0.624936566	0.648962409	0.60327365	0.61176998	0.617808497	6.298243394	0.769184551	0.12	0.000	4	4				
		Srsf11																			ALIVVPYAEGVIPDETK		907.5009	2
		Srsf11																			FHDPDSAVVAQHLTNTVFVDR		790.0608	3
		Srsf11																			LNHVAAGLVSPSLK		469.2769	3
		Srsf11																			TLFGFLGK		441.7578	2
P84104	ginine-rich splicing fa	Srsf3	164	49.4	5.442222844	5.592266229	5.792209286	4.58841878	4.945232113	4.593616295	4.725557552	3.02461689	3.757849994	4.816522496	27.41621142	5.10234854	0.19	0.051	9	9				
		Srsf3																			AFGYYGPLR		522.2691	2
		Srsf3																			DSCPLDCK	(57.0215),7C(57.02	497.702	2
		Srsf3																			NPPGFAFVEFEDPRDAADAVR		1160.5532	2
		Srsf3																			NPPGFAFVEFEDPRDAADAVR		774.0379	3
		Srsf3																			NPPGFAFVEFEDPRDAADAVRELDGR		723.3493	4
		Srsf3																			NRGPPPSWGR		562.2913	2
		Srsf3																			NRGPPPSWGR		375.1966	3
		Srsf3																			TLCGCR	(57.0215),5C(57.02	383.6704	2
		Srsf3																			VELSNGEK		438.2271	2
		Srsf3																			VRVELSNGEK		565.8118	2
		Srsf3																			VRVELSNGEK		377.5436	3
		Srsf3																			VYVGNLGNNGNK		624.8201	2
Q9CY97	iit A C-terminal doma	Ssu72	194	10.3	0.422709726	0.35914677	0.399667779	0.41269335	0.336653094	0.373472224	0.304536653	0	0.313687718	0.279442261	5.366004863	0.376326643	0.07	0.041	2	2				
		Ssu72																			AFLHTVCFY	7C(57.0215)	579.276	2
		Ssu72																			VAVVCSSNQNR	5C(57.0215)	617.3038	2
P83093	al interaction molecul	Stim2	746	15.3	0.364885175	0.463829763	0.41898638	0.39975367	0.405110962	0.440961304	0.508571252	0.51239963	0.513426183	0.507646835	7.823192588	0.425728416	0.05	0.005	9	9				
		Stim2																			ALSELTTCLR	8C(57.0215)	582.3081	2
		Stim2																			AQLPAHAPLAAHPR		483.9406	3
		Stim2																			ASGSAGAAASPSAAAAGER		780.374	2
		Stim2																			EGAECELSR	5C(57.0215)	525.7296	2
		Stim2																			MMKDLESLQTAEQSLMDLQERLE	(15.9949),1M(15.9	725.3527	4
		Stim2																			NFRDNNVK		503.7569	2
		Stim2																			PAGSLAR		336.1954	2
		Stim2																			QYAEQELEQVR		696.8413	2
		Stim2																			RQNAEMQLAIAK		686.8718	2
Q9D666	omain-containing prote	Sun1	913	9.3	0.184683916	0.188236501	0.206165775	0.20516202	0.18270524	0.188568005	0.260702833	0.25658497	0.259376373	0.22179885	4.747341958	0.194167509	0.04	0.031	7	6				
		Sun1																			GLSSSTSHDSSCSLR	12C(57.0215)	790.8521	2
		Sun1																			TDALTAHSAIHGTTSR		546.9446	3
		Sun1																			TDFMTFHHDHEVR		557.9159	3
		Sun1																			VASLSAQCHNHDER	8C(57.0215)	541.9146	3
		Sun1																			VLSNWGHPEYTCLYR	12C(57.0215)	632.3017	3
		Sun1																			VQHLELELNLLK		483.6205	3
Q8BJS4	omain-containing prote	Sun2	731	12.6	0.59234679	0.709136762	0.599294721	0.60682828	0.633334638	0.72504691	0.664830122	0.82464124	0.773840845	0.779149473	6.588923395	0.654728262	0.10	0.006	7	6				
		Sun2																			CSETYETK	1C(57.0215)	509.2133	2
		Sun2																			EGIVGVTEEQVHR		726.8757	2
		Sun2																			ILTNWGHPEYTCIYRFR	12C(57.0215)	742.7019	3
		Sun2																			IVQASQESEAR		609.3096	2
		Sun2																			RRGTGGSESSK		561.2864	2
		Sun2																			SALLDDHLHSEPYWSGDLR		737.6869	3
		Sun2																			SMTQEAFQESSVK		736.3403	2
Q99N50	ptotagmin-like protein	Sytl2	950	4.1	0	1.204933809	1.092255742	1.30710849	0	1.404486251	1.438804006	1.86929035	1.882243279	1.451240153	2.05265	1.001756859	0.49	0.015	3	3				
		Sytl2																			KLPTTGEVHIWVK		503.2925	3
		Sytl2																			TAPVALETENRGEMK		823.4143	2
		Sytl2																			TLNPVYNEILR		666.3695	2
Q64336	'-box brain protein 1 C	Tbr1	681	4.0	0.189908059	0.192705015	0	0	0.195547118	0.233817074	0.219021091	0	0.270224826	0.238408961	2.077354029	0.124413841	0.06	0.014	2	2				
		Tbr1																			HQTEMIITK		550.7921	2
		Tbr1																			YAMAGSFLQDQFVSNYAK		1020.4802	2
		Tbr1																						
Q3UUI3	zyme A thioesterase T	Them4	230	38.3	3.213267608	2.757198131	2.982216684	2.86076969	3.231591057	2.912282271	2.493020135	2.16786941	2.071377331	2.757289039	20.7370838	2.948811567	0.14	0.036	8	8				
		Them4																			AIQEFQTLFVDSK		763.3984	2
		Them4																			AQQFTR		375.6983	2
		Them4																			DYALPNPSWTK		646.3195	2
		Them4																			KCEDGSWK	2C(57.0215)	505.224	2
		Them4																			KPIPLLSVVVVNSQLQK		931.5773	2
		Them4																			LFVSCTIQSIDEK	5C(57.0215)	770.3898	2
		Them4																			LLFDQFMK		521.2755	2
		Them4																			LLFDQFMK	7M(15.9949)	529.2729	2
		Them4																			TLYTEATALFIK		685.8819	2
P70318	Nucleolysin TIAR OS	Tial1	392	44.1	1.026491936	0.96816297	0.948160887	0.89602511	0.876166259	0.862927287	0.820819961	0.69914789	0.760089761	0.769146529	22.57959597	0.910288503	0.04	0.004	12	9				
		Tial1																			DAAAALAAMNGRK		630.3298	2
		Tial1																			DTSNHFHVFVGDLSPEITTEDIK		867.7537	3
		Tial1																			DVTEVLILQLFSQIGPCK	17C(57.0215)	1030.5583	2
		Tial1																			FEDVVNQSSPK		625.3065	2
		Tial1																			FSTHESAAHAIVSVNGTTIEGHVVK		864.4453	3
		Tial1																			GYGFVSFYNK		591.2849	2
		Tial1																			LDAENAIVHMGGQWLGGR		962.4783	2
		Tial1																			LDAENAIVHMGGQWLGGR		641.9879	3
		Tial1																			NCTVYCGGIASGLTDQLMR	(57.0215),6C(57.02	1058.4846	2

		Tial1																			NCTVYCGGIASGLTDQLMR	949),2C(57.0215),6C	1066.482	2
		Tial1																			QLRFEDVVNQSSPK		549.6214	3
		Tial1																			QTFSPFGQIMEIR		777.3927	2
		Tial1																			TLYVGNLSR		511.7851	2
		Tial1																			VNWATTPSSQK		609.8093	2
Q8CGF5	omain-containing prote	Tlcd4	276	7.2	0.362223274	0.42030772	0.438865903	0.41414009	0.354037201	0.439495518	0.461802753	0.54909528	0.499437089	0.443253722	3.804311637	0.413369287	0.11	0.015	2	2				
		Tlcd4																			AAVGSGAMEASTK		590.2873	2
		Tlcd4																			AAVGSGAMEASTK	8M(15.9949)	598.2847	2
		Tlcd4																			KIEWNSR		466.7511	2
Q8VD26	smembrane protein 14	Tmem143	458	8.5	0.4324935	0	0.408435778	0.62172326	0	0	0	0	0	0	4.47389675	0.206031809	0.05	0.000	3	3				
		Tmem143																			EPCDWAQQYR	3C(57.0215)	676.788	2
		Tmem143																			SAQALELAHVLYYR		817.4384	2
		Tmem143																			STSNNSELLSALALR		788.4204	2
Q61033	d polypeptide 2, isofor	Tmpo	693	3.9	0	0	0	0.15191371	0.170105946	0	0	0	0	0	1.94805	0.064403932	0.03	0.000	12	2				
		Tmpo																			SRAQPLR		414.2459	2
		Tmpo																			SYEAAASALQIAAHTAFVAK		1010.5285	2
Q8BSH3	omyosin alpha-1 chai	Tpm1	284	14.1	1.099357943	0.9505065	0	0	0	0	0	0	0	0	7.591928972	0.1901013	0.03	0.000	36	3				
		Tpm1																			AISEELDHALNDMTSM	I(15.9949),16M(15.9	603.594	3
		Tpm1																			QLEDELVSLQK		651.351	2
		Tpm1																			SIDDLEDELYAQK		769.8646	2
E9Q7M2	2 domain family protei	Tsc22d2	769	3.0	0.277426396	0.284022898	0	0.30649951	0	0	0	0	0	0	1.634163198	0.118104481	0.07	0.000	5	2				
		Tsc22d2																			HSNTLEQTAER		643.3101	2
		Tsc22d2																			NSLLERENALLK		700.3988	2
Q8BJU2	Tetraspanin-9 OS	Tspan9	239	15.5	0.862968694	0.845177726	0.897117428	0.82213013	0.691250723	0.548364957	0.681278478	0.50425803	0.754092654	0.441534902	8.172084347	0.760808193	0.09	0.018	3	3				
		Tspan9																			CCMENSQGCGR	15),2C(57.0215),9C	679.7388	2
		Tspan9																			EGLLLYNTENNVGLK		838.9463	2
		Tspan9																			NAWNIIQAEMR		673.3377	2
P68372	ubulin beta-4B chain (	Tubb4b	445	9.0	59.20744569	64.3904646	69.81938832	65.3992657	63.14894918	61.0928083	60.83310541	45.2191241	48.07065874	51.22768235	34.09812284	64.77017523	1.90	0.030	38	1				
		Tubb4b																			GHYTEGAELVDSVLDVVR		979.9945	2
		Tubb4b																			GHYTEGAELVDSVLDVVR		653.6654	3
		Tubb4b																			GHYTEGAELVDSVLDVVRK		1044.0419	2
		Tubb4b																			GHYTEGAELVDSVLDVVRK		696.3637	3
		Tubb4b																			GHYTEGAELVDSVLDVVRK		522.5246	4
		Tubb4b																			INVYYNEATGGK		664.8276	2
		Tubb4b																			SGPFGQIFR		504.7667	2
Q9CY34	conjugating enzyme U	Ube2f	185	29.2	0.635426632	0.572408529	0.647868475	0.61417205	0.598309922	0.476761479	0.51454739	0.40803294	0.447516091	0.526041405	14.91231332	0.58190409	0.04	0.003	4	4				
		Ube2f																			EHSIDGTGWAPTR		713.8391	2
		Ube2f																			EHSIDGTGWAPTR		476.2285	3
		Ube2f																			EVAELEANLPCTCK	(57.0215),13C(57.02	817.3816	2
		Ube2f																			LHCFQLTVSPDEGYYQGGK	3C(57.0215)	1100.0125	2
		Ube2f																			LHCFQLTVSPDEGYYQGGK	3C(57.0215)	733.6774	3
		Ube2f																			VHFPDPNK		477.2456	2
E0CXP6	ssociated (UMA) dom	Umad1	133	15.0	0.214276595	0	0.193594248	0.21706149	0.223888809	0.250269034	0.276582576	0	0.299844942	0.281165842	7.625938297	0.176962717	0.02	0.002	2	2				
		Umad1																			TSEAEGSQALETGK		704.3335	2
		Umad1																			MFHFFR	N-term(42.0106)	463.7207	2
		Umad1																			AHSDYMLYVYDSR		810.3615	2
Q9CPX8	me b-c1 complex subu	Uqcr11	56	32.1	6.891875023	5.340312784	4.836327002	4.65100353	4.725718805	4.561205943	4.51760009	3.22658118	3.758617456	3.679207939	19.51738751	4.822913614	0.25	0.019	2	2				
		Uqcr11																			LILDWVPYINGK		715.9057	2
		Uqcr11																			YRELAR		404.2272	2
E9Q271	VD repeat domain 17 (	Wdr17	1322	2.9	2.65739354	2.666505755	2.030328552	2.72574295	2.152490437	2.230482277	1.792622472	1.76570297	2.047210796	1.989214808	2.76589677	2.361109993	0.85	0.024	3	3				
		Wdr17																			GQDDSLLPQNYCK	12C(57.0215)	769.3511	2
		Wdr17																			NGIFYIAWSHK		668.3458	2
		Wdr17																			SWDEVIGNTDNAIR		530.5899	3
Q6ZQL4	peat-containing protei	Wdr43	677	5.8	1.49496136	0	1.699689439	0	0	0	0	0	0	0	3.62783068	0.339937888	0.09	0.000	2	2				
		Wdr43																			FTTIRPNESQPSDGITGLYFLSGAVHDR		770.3874	4
		Wdr43																			YIVEWSTQTCK	10C(57.0215)	707.8372	2
Q9CX30	Protein YIF1B OS	Yif1b	311	8.4	0	0	0.751382206	0	0.79426423	0	0	0	0	0	4.18005	0.309129287	0.07	0.000	2	2				
		Yif1b																			YYFAVDTVYVGK		712.8584	2
		Yif1b																			MHATGLAAPAGTPR	N-term(42.0106)	696.8562	2
Q505B7	Protein archease OS	Zbtb8os	168	28.6	0.526807639	0.501082159	0.572808451	0.47410744	0.47614494	0.492299271	0.464514707	0.31021879	0.380659156	0.346900775	14.54910382	0.503288451	0.03	0.038	5	5				
		Zbtb8os																			FSADEYFIPR		622.8009	2
		Zbtb8os																			HPQGTEVK		448.2352	2
		Zbtb8os																			SIGWGEEFSLSK		670.33	2
		Zbtb8os																			AQEEEDVRDYNLTEEQK	N-term(42.0106)	1069.4796	2
		Zbtb8os																			MAQEEEDVR	N-term(42.0106)	574.7481	2
O88878	ype zinc finger protei	Zfand5	213	12.2	0	0	0	0.17182374	0.177540603	0	0	0	0	0	6.1033	0.069872869	0.01	0.000	2	2				
		Zfand5																			ADAGLNNCEGAAGSTSEK	8C(57.0215)	876.3786	2
		Zfand5																			HNCPYDYK	3C(57.0215)	548.7294	2
O54836	nger matrin-type prote	Zmat3	290	10.7	0.490604418	0.994407897	0.950800078	0	0	0	0	0	0	0	5.590152209	0.389041595	0.07	0.000	2	2				
		Zmat3																			KEGSEFKMVATR		461.5747	3
		Zmat3																			LCNVTLNSAQQAQAHYQGK	2C(57.0215)	711.0165	3
Q61624	nc finger protein 148 (	Znf148	794	2.1	0	0.310770223	0.367766001	0	0	0	0	0	0	0	1.07055	0.135707245	0.13	0.000	2	2				
		Znf148																			LEGLFLK		410.2524	2
		Znf148																			PFQCSQCDMR	(57.0215),7C(57.02	664.7626	2

Supplementary Table 2. Differentially acetylated proteins (DAPs) in EFV-treated *vs* control 5XFAD female mice.

Accession #	Protein name	Gene name	Peptide	Acetylation position	Modifications	Peptide Length	Number of Missed Cleavages	M/Z	Charge	Control1	Control2	Control3	Control4	Control5	Treated1	Treated2	Treated3	Treated4	Treated5	Fold change (Tx/Cntr)	P value
Q8BGQ7	Alanine--tRNA ligase, cytoplasmic OS	Aars1	ALNEALkLFK	K876	K7(42.0106)	10	1	594.853	2	0.575166597	0.44546728	0.62784504	0.59123647	0.50786955	0	0	0.49278405	0	0	0.18	0.008
Q99K67	pha-aminoadipic semialdehyde synthase, mitochondrial	Aass	GITkLGYK;HIkGITkLGYK	K52	K3(42.0106),K16(42.0106)	11	2	671.398	2	9.06004028	10.9333869	8.71637881	7.79944408	5.26281813	4.6743114	5.6358801	6.63823916	2.84975374	5.44507293	0.60	0.021
Q99K67	pha-aminoadipic semialdehyde synthase, mitochondrial	Aass	LGYkVLIQPSNR	K56	K4(42.0106)	12	1	715.4117	2	0.692846746	0.75017184	0.98571152	0.88135158	0	0	0.63189801	0	0	0	0.19	0.040
Q99KI0	Aconitate hydratase, mitochondrial OS	Aco2	YLSkTGR;kYLSkTGR	K309	K1(42.0106),K14(42.0106)	8	2	518.7929	2	9.398643075	7.55800239	8.38970144	7.4402449	6.44520906	5.64209076	6.83893467	6.52354253	3.95684681	7.06168626	0.77	0.041
Q9CQR4	Acyl-coenzyme A thioesterase 13 OS	Acot13	VLEkVTLVSAAPEK	K27	K4(42.0106)	14	1	763.4454	2	3.759716536	3.43565331	2.60712891	2.93552823	1.18755823	0.98734793	1.41856858	1.70254125	0.58386967	1.96632402	0.48	0.028
Q8VCW8	Aedium-chain acyl-CoA ligase ACSF2, mitochondrial O	Acsf2	ISHFkIPR	K581	K5(42.0106)	8	1	520.306	2	2.171836148	1.88572437	2.28776793	1.74809933	1.89635845	1.56828331	1.62267279	1.84192679	1.36503985	1.64976818	0.81	0.016
Q91WC3	Long-chain-fatty-acid--CoA ligase 6 OS	Acsl6	WLPEGTLkIIDR	K551	K8(42.0106)	12	1	741.9194	2	0	0	0	0	0.70423114	0.66194477	0.6666165	0.77424745	0.73425936	0.67972293	4.99	0.015
P68033	Actin, alpha cardiac muscle 1 OS	Actc1	/DDMEk;YPIEHGIITNWDDMEkIV	K86	M14(15.9949),K25(42.0106)	27	1	879.6673	4	3.094519947	2.58496223	1.99636141	1.88562506	2.07089245	1.31688456	1.68410844	1.82815317	1.57677167	1.56060548	0.68	0.028
P61161	Actin-related protein 2 OS	Actr2	VLEkLGVTVR	K388	K4(42.0106)	10	1	578.3583	2	0.728642925	0.58997765	0.62837115	0.64556193	0.40096038	0	0.65729606	0	0	0	0.22	0.020
P51830	Adenylate cyclase type 9 OS	Adcy9	FPFGkAIEK	K1326	K5(42.0106)	9	1	539.8001	2	0	0	0	0	0	0.55226688	0.5630872	0	0.38438274	0.52818906	inf	0.019
Q9QYB5	Gamma-adducin OS	Add3	TkITWMK	K465	K2(42.0106)	7	1	475.2624	2	0.198486987	0.28259784	0	0.33287232	0.27308997	0	0	0	0	0	0.00	0.020
E9QQ10	A kinase (PRKA) anchor protein (yotiao) 9 OS	Akap9	QTLQHVASkLQHVAQK	K3397	K9(42.0106)	16	1	620.0146	3	0.553747694	0.62434436	0.50270979	0.65627878	0.5211408	0	0	0	0.43989554	0.45474835	0.31	0.021
P24549	Aldehyde dehydrogenase 1A1 OS	Aldh1a1	IQHTkIFINNEWHNSVSGK	K22	K5(42.0106)	19	1	765.394	3	2.952189299	3.32564288	3.18358552	2.63615148	2.86380109	1.93083617	2.14578166	2.23402971	2.0539302	2.89950261	0.75	0.009
Q9DBF1	Alpha-aminoadipic semialdehyde dehydrogenase OS	Aldh7a1	QGLSSSIFTkDLGR	K462	K10(42.0106)	14	1	775.9122	2	1.214562691	0.98580578	1.38464956	1.14132253	1.59374657	1.66794616	2.02337498	1.41197421	1.98120942	1.79252656	1.40	0.010
P84091	AP-2 complex subunit mu OS	Ap2m1	MIGGLFIYNHk	K11	K11(42.0106)	11	0	667.8499	2	0.73979647	0.65725315	0.55224209	0.60725888	0.60308238	0	0	0	0	0.46814112	0.15	0.003
Q9JME5	AP-3 complex subunit beta-2 OS	Ap3b2	DSLkLEAMK	K54	K4(42.0106)	9	1	538.7864	2	1.881618754	1.84682591	1.37919311	1.33366175	0.81483497	0.7358843	0	1.10629581	0	0	0.25	0.008
P61205	ADP-ribosylation factor 3 OS	Arf3	TTILYkLK	K36	K6(42.0106)	8	1	511.3182	2	0.935117192	0.75068191	0.82757341	0.89680627	0.68450831	0.52794658	0.68771034	0.78030428	0.49773755	0.71555631	0.78	0.040
Q6P9R4	Rho guanine nucleotide exchange factor 18 OS	Arhgef18	QTAVQQQIPTkLAASTK	K1285	K11(42.0106)	17	1	928.0178	2	0.481882366	0.34079808	0.5319208	0.44339472	0.28788086	0	0.42903483	0	0	0	0.21	0.014
Q9CVB6	Actin-related protein 2/3 complex subunit 2 OS	Arpc2	DYLHYHIk;DYLHYHIkCSK	K256	K8(42.0106)	8	0	565.7851	2	2.365421925	2.57120615	2.39707398	2.71179818	2.57547329	1.82740397	1.65829746	2.34412047	2.50101158	1.61822784	0.79	0.039
Q91YI0	Argininosuccinate lyase OS	Asl	ELIGEAAGkLHTGR	K106	K9(42.0106)	14	1	747.4071	2	0.365177118	0.3985694	0.3832385	0.40829361	0	0	0	0	0	0	0.00	0.016
Q8R3P0	Aspartoacylase OS	Aspa	EAFAkTTK	K296	K5(42.0106)	8	1	469.2531	2	0	0	0	0	0	0.28411636	0.44044456	0.42956814	0.29943307	0	inf	0.022
P14231	odium/potassium-transporting ATPase subunit beta-2 O	Atp1b2	VAFkLR	K284	K4(42.0106)	6	1	388.2449	2	1.266401656	1.17472058	0.96826587	0.93448463	0.6297218	0.56418395	0.43441794	0.74717656	0	0.64371041	0.48	0.017
A2ALL9	Calcium-transporting ATPase OS	Atp2b3	VWPkLR	K758	K4(42.0106)	6	1	420.7581	2	1.535887182	1.12413363	1.31823194	1.0812745	1.13905235	1.00291552	0.67060152	1.090358	0.73330817	0	0.56	0.045
Q9CPQ8	ATP synthase subunit g, mitochondrial OS	Atp5mg	LATFWHYAk	K35	K9(42.0106)	9	0	589.8033	2	5.898965659	5.72903853	4.1928046	4.01664924	4.04370804	3.32367081	2.81595244	4.00807331	3.39288271	2.92735823	0.69	0.021
P97450	ATP synthase-coupling factor 6, mitochondrial OS	Atp5pf	ELDPVQkLFVDK	K41	K7(42.0106)	12	1	736.9034	2	21.2850984	14.2121778	10.9118513	8.95703276	9.74470115	0	9.43722779	7.42322855	3.89984455	8.40976423	0.45	0.037
Q9Z1G4	V-type proton ATPase 116 kDa subunit a 1 OS	Atp6v0a1	PPTYNKTNk;TNkFTHGFQNIVDA	K376	K3(42.0106)	21	1	815.4046	3	0	0	0	0	0.50436338	0.54666778	0.57112494	0.84901975	0.54209803	0.46946664	5.91	0.005
Q9Z1G4	V-type proton ATPase 116 kDa subunit a 1 OS	Atp6v0a1	VWFIkVR	K303	K5(42.0106)	7	1	495.3002	2	4.878973338	3.90651861	3.7452397	4.38138586	3.96969429	3.12020785	3.94417794	4.01609584	3.18972844	2.89294674	0.82	0.042
Q3U3J1	2-oxoisovalerate dehydrogenase subunit alpha OS	Bckdha	HLQTYGEHYPLDHFDk	K446	K16(42.0106)	16	0	681.3182	3	2.058652944	1.88894803	0.86788579	1.24715142	0.86984632	0.55188118	0.66170765	0.71595883	0.32275635	1.20064868	0.50	0.051
Q8R016	Bleomycin hydrolase OS	Blmh	IGPITPLQFYKEHVk	K259	K15(42.0106)	15	1	604.6733	3	2.549033866	2.50349657	0	2.62705871	2.49876707	0	0	0	0	0	0.00	0.016
Q9Z0S1	3'(2'),5'-bisphosphate nucleotidase 1 OS	Bpnt1	VGGAGNkIIQLIEGK	K224	K7(42.0106)	15	1	769.9486	2	0	0	0.5231637	0.63456036	0.55655473	0.53930016	0.90054159	0.88303963	0.6612304	0.78913562	2.20	0.041
P00920	Carbonic anhydrase 2 OS	Ca2	HWGYSk;SHHWGYSkHNGPENW	K9	N-term1(42.0106),K18(42.0106)	17	1	728.9964	3	9.173396196	8.13112925	7.56653146	8.39807039	6.71018202	7.13247522	5.89657518	7.12706087	2.40002322	5.29657539	0.70	0.047
P40124	Adenylyl cyclase-associated protein 1 OS	Cap1	GkINSITVDNCK	K365	K2(42.0106),C20(57.0215)	12	1	695.8533	2	0.476510342	0	0.45570616	0	0.4984453	0.63228134	0.55608839	0.7195572	0.45747446	0.67273338	2.12	0.049
Q8BRT1	CLIP-associating protein 2 OS	Clasp2	TFQDGATkLLHNHLR	K865	K8(42.0106)	15	1	598.3199	3	2.142154203	2.09360339	2.38669454	2.47201758	1.91061264	1.37091048	1.81147299	1.60708803	2.2783189	1.15584856	0.75	0.043
O88696	-dependent Clp protease proteolytic subunit, mitochondri	Clpp	QLYNIYAkHTK;kQLYNIYAkHTK	K207	K1(42.0106),K18(42.0106)	12	2	530.9594	3	22.40166442	18.8242919	15.4994193	14.6410594	13.5169066	13.0269023	12.9026801	14.3058061	7.94906163	13.3751369	0.73	0.049
Q06890	Clusterin OS	Clu	ELHDPHYFSPIGFPHkR	K213	K16(42.0106)	17	1	707.0218	3	1.171965563	1.23309474	1.18807652	1.18867052	0.74045589	0.7336965	0.84905283	0.98639563	0.66022919	0.78911841	0.73	0.028
P16330	2',3'-cyclic-nucleotide 3'-phosphodiesterase OS	Cnp	MVSADAYkIIPGSR	K87	K8(42.0106)	14	1	775.4057	2	5.530906897	16.4343489	14.5107136	15.617988	2.31890938	28.3455939	22.1594163	22.0723011	61.130412	25.055989	2.92	0.046
P61202	COP9 signalosome complex subunit 2 OS	Cops2	EDDPkAALSSFQK	K48	K5(42.0106)	13	1	739.362	2	0.745398565	0.70108202	0.60450211	0	0.50388352	0	0	0	0	0	0.00	0.019
Q8BMS4	quinone biosynthesis O-methyltransferase, mitochondria	Coq3	PLSGMkILDVGCGGGLLTEPLGR	K150	(15.9949),K15(42.0106),C30(57.02	23	1	1199.627	2	1.093825405	0.89640669	0.87700587	1.65432102	1.30010033	1.56089875	1.61852081	1.49699157	2.38714739	2.05461046	1.57	0.019
Q9Z140	Copine-6 OS	Cpne6	LDNkDLFSK	K166	K4(42.0106)	9	1	561.2955	2	0.876551478	0.8299429	0.67093838	0.58941443	0	0	0.60108241	0	0	0	0.20	0.046
Q3UNZ8	Quinone oxidoreductase-like protein 2 OS	Cryzl2	CkLAVQR	K203	C1(57.0215),K11(42.0106)	7	1	458.7553	2	2.546082787	1.93729821	1.93659072	1.89714533	1.12943273	0	0	1.33092834	0	1.54015447	0.30	0.017
Q9D4H8	Cullin-2 OS	Cul2	YLHPSSYTkVIHECQQR	K261	K9(42.0106),C23(57.0215)	17	1	730.0249	3	0.928361764	1.30219772	0.87633375	1.04691579	0.74317568	0.59182346	0.60487082	0.84727233	0.86470446	0.53371578	0.70	0.040
Q80U19	Disheveled-associated activator of morphogenesis 2 OS	Daam2	SFNWVkLNEER	K612	K6(42.0106)	11	1	732.3675	2	0.432689334	0.40550128	0	0.44745488	0.46321033	0	0	0	0	0	0.00	0.016
P31786	Acyl-CoA-binding protein OS	Dbi	AAEEVkR	K14	K6(42.0106)	7	1	422.7298	2	1.48033495	1.3089651	1.11404389	1.22027014	0.88219044	0.64715678	0.91823099	1.10382606	0.50808856	1.0213168	0.70	0.044
Q62167	ATP-dependent RNA helicase DDX3X OS	Ddx3x	GFYDkDSSGWSSSK	K55	K5(42.0106)	14	1	796.8468	2	0.493320123	0.39051683	0	0.29171731	0.29352863	0	0	0	0	0	0.00	0.023
E9PUQ8	Diacylglycerol kinase delta OS	Dgkd	DSLPALNTkILYPSVR	K720	K9(42.0106)	16	1	915.0119	2	0.897167	0	1.81353313	1.57940557	1.10770134	0.41977379	0	0	0.5320545	0	0.18	0.045
Q8CI75	DIS3-like exonuclease 2 OS	Dis3l2	SYSFQkVGK	K807	K6(42.0106)	9	1	543.2849	2	1.313606992	1.12492722	1.02014303	0.9702155	0	0.88119517	0	0	0	0	0.20	0.042
Q8BMF4	acetyltransferase component of pyruvate dehydrogenase	Dlat	ELNkMLEGKGk;ELNkMLEGkGK;	K461	K4(42.0106),K20(42.0106)	11	2	665.8554	2	14.62296562	11.1233111	11.711069	10.6607006	10.5304372	8.86990227	9.17962838	10.3142242	8.78345072	10.9845973	0.82	0.049
Q8BMF4	acetyltransferase component of pyruvate dehydrogenase	Dlat	kELNkMLEGK	K457	K1(42.0106),K14(42.0106)	10	2	637.3446	2	15.02681285	14.1971447	12.1882339	11.2220802	10.0837001	7.86848059	9.66737183	10.3112856	6.70425769	11.1985895	0.73	0.025
O08749	Dihydrolipoyl dehydrogenase, mitochondrial OS	Dld	NTK;FkLNTkVTGATK;QGFkFkLN	K273	K2(42.0106)	6	1	396.7343	2	32.05332255	28.1707623	30.5496569	27.1939077	29.5743071	24.0043526	28.2857684	26.6558043	18.2884272	25.3076283	0.83	0.041
B1AZP2	Disks large-associated protein 4 OS	Dlgap4	LFFTkAPSMEGTAGK	K153	K5(42.0106)	15	1	813.9134	2	1.816034294	0	1.6990232	2.75886802	2.73584657	1.73087303	0	0	0	0	0.19	0.048
Q91ZU6	Dystonin OS	Dst	WINQHLMkVR	K51	K8(42.0106)	10	1	683.8742	2	0	0.39066854	0.3650727	0.38328348	0.21418522	0	0	0	0	0	0.00	0.023
Q9DD18	D-aminoacyl-tRNA deacylase 1 OS	Dtd1	HWSkSVMDK	K65	K4(42.0106)	9	1	580.2818	2	0.745565756	0.68914076	1.21870713	1.1892838	1.01861333	1.20620686	1.44055286	1.07866096	1.76428743	1.62385424	1.46	0.028
Q9JHU4	Cytoplasmic dynein 1 heavy chain 1 OS	Dync1h1	EENLkMVWR	K445	K5(42.0106)	9	1	623.816	2	0.531662929	0.6466287	0.55068363	0	0.71074483	0	0	0	0	0	0.00	0.018
P58252	Elongation factor 2 OS	Eef2	LYMkAR	K605	K4(42.0106)	6	1	412.2283	2	0.469220531	0.38957619	0.60432617	0.52555201	0.42119008	0.33484353	0	0	0	0.45294316	0.33	0.026
P48193	Protein 4.1 OS	Epb41	VLkISYK	K440	K3(42.0106)	7	1	446.7787	2	3.588939063	3.14336015	2.69929935	2.7529784	2.59958375	2.21507003	2.20160132	2.76615909	2.12774588	2.28883467	0.78	0.022
P42567	Epidermal growth factor receptor substrate 15 OS	Eps15	ESINLQkLQAQK	K386	K7(42.0106)	12	1	721.404	2	0.330113495	0.37705901	0.32611421	0.38009549	0	0	0	0	0	0	0.00	0.017
Q99LC5	ctron transfer flavoprotein subunit alpha, mitochondrial	Etfa	VAGVAkVLVAQHDAYK	K75	K6(42.0106)	16	1	855.9804	2	2.961333339	2.55075946	2.65896093	2.38429238	2.05061094	1.900117	2.24265703	1.86669964	1.36376511	2.29319807	0.77	0.031
P47754	F-actin-capping protein subunit alpha-2 OS	Etfa	ICFkFDHLR	K97	C2(57.0215),K13(42.0106)	9	1	639.3265	2	5.418683857	4.73829672	3.92100641	3.93041663	3.35866813	2.79121758	3.70218849	3.88157048	2.77098994	2.96369753	0.75	0.046
P97807	Fumarate hydratase, mitochondrial OS	Fh	VPTDkYYGAQTVR	K63	K5(42.0106)	13	1	770.3936	2	158.3545986	132.055588	118.227776	105.18731	99.0238149	91.0512286	92.0956745	105.683602	65.9587571	106.418275	0.75	0.050
Q91Z49	UAP56-interacting factor OS	Fyttd1	ANLTFSkGGSR	K308	K7(42.0106)	11	1	590.3094	2	0.491791098	0.46499835	0.68947715	0.54464091	0.63106801	0.62023695	0.70019976	0.74984749	0.65827038	0.83993624	1.26	0.031
P62812	Gamma-aminobutyric acid receptor subunit alpha-1 OS	Gabra1	TFNSVSkIDR	K417	K7(42.0106)	10	1	604.8171	2	0.607368635	0.65166902	0.69127034	0.60051184	0.68473093	0.66537528	0.76857126	0.84261656	0.89221348	0.72267973	1.20	0.028
Q99KY4	Cyclin-G-associated kinase OS	Gak	AIIQEVCFLkK	K90	C7(57.0215),K19(42.0106)	11	1	695.8917	2	0	0	0	0.43108494	0	0	0.47081901	0.53068587	0.50749347	0.43691995	4.51	0.050
Q9D172	lotransferase-like class 1 domain-containing protein 3, m	Gatd3	ALGAkHCVkGVTEAHVDQK	K217	(42.0106),C16(57.0215),K27(42.01	19	2	711.3687	3	1.474840025	1.41711202	1.59062604	1.47544183	1.38409523	1.21586314	1.26793844	1.19828663	0.94261733	1.30120502	0.81	0.007
P03995	Glial fibrillary acidic protein OS	Gfap	HLQEYQDLLNVk	K353	K12(42.0106)	12	0	771.4016	2	4.579921101	3.9557146	3.04045431	2.8030205	2.24903401	1.2822019	2.5101473	2.81122044	2.04723628	2.01110443	0.64	0.047
D3Z7P3	Glutaminase kidney isoform, mitochondrial OS	Gls	FITALkSTGLR	K169	K6(42.0106)	11	1	624.8691	2	0	0	0.53016983	0.98253865	0	0.81573365	1.46606144	1.00253692	0.54526521	1.08346431	3.25	0.028
P26443	Glutamate dehydrogenase 1, mitochondrial OS	Glud1	.GVkINPK;AGVkINPkNYTDNELE	K187	K4(42.0106),K17(42.0106)	17	2	1009.0154	2	7.587919634	6.60238054	9.15568982	6.11719946	9.22922293	6.09344659	5.35307172	6.5189968	4.09130467	5.75006709	0.72	0.025
P26443	Glutamate dehydrogenase 1, mitochondrial OS	Glud1	GASIVEDkLVEDLkTR;LVEDLkTR	K90	K8(42.0106),K23(42.0106)	16	2	929.0018	2	7.327654892	5.70872242	3.26330367	2.79376275	3.7782557	1.31638213	1.85131274	2.39008647	1.64529482	3.00203091	0.45	0.037
Q9CQI3	Glia maturation factor beta OS	Gmfb	FIVYSYkYQHDDGR	K74	K7(42.0106)	14	1	916.9337	2	2.919033611	2.87803051	2.85793525	2.70290671	2.06736834	1.99746276	2.07702952	2.59322129	2.09524718	2.34445571	0.83	0.047
P62874	e nucleotide-binding protein G(I)/G(S)/G(T) subunit be	Gnb1	GHLAkIYAMHWGTDSR	K57	K5(42.0106)	16	1	942.9622	2	39.09155266	35.8180457	32.5403703	42.5896938	29.6781804	25.0978181	29.8429292	35.7922223	22.7755702	23.7916924	0.76	0.034
P29387	Guanine nucleotide-binding protein subunit beta-4 OS	Gnb4	GHLAkIYAMHWGYDSR	K57	K5(42.0106)	16	1	649.6491	3	0.983751135	0.99861741	0.75195727	0.85622615	0.80888874	0	0.64675702	0	0	0	0.15	0.003
P05202	Aspartate aminotransferase, mitochondrial OS	Got2	GAFTVVCKDAEEAkR;VGAFTVV	K302	K6(42.0106)	7	1	430.709	2	19.80991796	16.1068975	16.4958329	11.3227587	12.7447257	11.5082702	11.3107381	9.40075779	9.80895195	10.2935289	0.68	0.030
P06745	Glucose-6-phosphate isomerase OS	Gpi	GLHHkILLANFLAQTEALMK	K423	K5(42.0106),M28(15.9949)	20	1	769.4278	3	0	0.30416111	0.9094207	0	2.16035512	1.95630151	2.66226703	3.28607596	0.64899644	1.83735634	3.08	0.048
P43275	Histone H1.1 OS	H1-1	AITTkVSVK	K121	K5(42.0106)	9	1	494.8055	2	1.649424589	1.45995371	2.09077179	1.36809951	1.3678266	0.88284988	1.19342104	0.95362856	0.8861934	1.52741549	0.69	0.027
P43274	Histone H1.4 OS	H1-4	GTGASGSFkLNK	K106	K9(42.0106)	12	1	604.8171	2	17.33932963	18.482568	20.4770629	16.844298	15.677214	12.430835	15.4046978	14.8626943	10.3306546	17.3567355	0.79	0.041
P10854	Histone H2B type 1-M OS	H2bc14	kAVTkAQK	K17	K1(42.0106),K14(42.0106)	8	2	957.5727	1	402.9754518	380.318423	404.019447	363.922258	338.800914	297.175393	323.73854	349.302312	242.177999	368.538099	0.84	0.048
Q64478	Histone H2B type 1-H OS	H2bc9	PEPAKSAPAPk;PEPAkSAPAPkK	K12	N-term1(42.0106),K21(42.0106)	12	2	652.864	2	65.04449716	50.8566964	67.7499887	53.3975561	55.3729475	47.4149363	51.6935502	48.3948781	45.7651877	49.0636947	0.83	0.037
Q64478	Histone H2B type 1-H OS	H2bc9	PEPAkSAPAPKk;PEPAkSAPAPkK	K6	n1(42.0106),K15(42.0106),K31(42	12	2	673.8693	2	65.04449716	50.8566964	67.7499887	53.3975561	55.3729475	47.4149363	51.6935502	48.3948781	45.7651877	49.0636947	0.83	0.037
P62806	Histone H4 OS	H4c1	DAVTYTEHAk;DAVTYTEHAkR	K78	K10(42.0106)	11	1	666.8308	2	1.52259297	1.36494523	1.1699668	1.43030885	1.30227722	0.85329887	1.09630228	0.97710944	0.76148637	0.94303647	0.68	0.001
Q8BMS1	Trifunctional enzyme subunit alpha, mitochondrial OS	Hadha	MFEkLEK	K129	K4(42.0106)	7	1	483.7518	2	12.71244502	11.9352006	12.4881303	10.8965551	9.51261398	8.5429221	9.74434626	9.31874567	5.99232148	10.1945897	0.76	0.021
Q8BMS1	Trifunctional enzyme subunit alpha, mitochondrial OS	Hadha	ILQEGVDPkK	K569	K9(42.0106)	10	1	584.8322	2	1.254692239	1.06969459	0.80536487	0.72358982	0.82461208	0.50248247	0.65937054	0.42774609	0.44280439	0.66841682	0.58	0.012
Q99KB8	Hydroxyacylglutathione hydrolase, mitochondrial OS	Hagh	IGALTHkVTHLSTLQVGSLSVK	K137	K7(42.0106)	22	1	777.7846	3	0.457003863	0.49717358	1.09346289	0.75814165	0.41996317	0.47846195	0	0	0	0	0.15	0.010
Q9ESM3	Hyaluronan and proteoglycan link protein 2 OS	Hapln2	WSkVEPGELR	K73	K3(42.0106)	10	1	621.8275	2	0.442838325	0.43327076	0	0.74544858	0.69332859	0	0.37364243	0	0	0	0.16	0.041

A8DUK4	Beta-globin OS	Hbb-bs	AAVSGLWGkVNADEVGGEALGR	K18	K9(42.0106)	22	1	1099.5635	2	5.178551099	3.27511722	8.94361657	3.81798392	6.90228247	8.74539729	9.25098073	10.3425095	6.68524963	11.4125823	1.65	0.025
Q99L13	3-hydroxyisobutyrate dehydrogenase, mitochondrial OS	Hibadh	LLAkILNMSSGR	K241	K4(42.0106)	12	1	672.887	2	47.03019273	39.8260219	36.9717567	30.2322976	32.116049	31.0317073	31.2029261	27.7434456	21.4739508	31.1930246	0.77	0.044
P70349	Adenosine 5'-monophosphoramidase HINT1 OS	Hint1	AQVAQPGGDTIFGkIIR	K21	K14(42.0106)	17	1	907.0019	2	7.460047255	5.29861803	6.60576786	5.03729568	6.71718383	6.28922146	9.41549605	9.28337712	9.23183913	7.78933207	1.35	0.022
Q61666	Protein HIRA OS	Hira	ATYIGPSTVFGSSGkLANVEQWR	K114	K15(42.0106)	23	1	1255.6373	2	0	0	0	0.57763843	0.73580338	0.85891165	0.94579144	1.12955271	0.48009379	1.0792236	3.42	0.015
P20029	Endoplasmic reticulum chaperone BiP OS	Hspa5	NkITITNDQNR	K524	K2(42.0106)	11	1	679.8547	2	0.465151513	0.37652537	0.65820013	0.76278456	0.78918186	0.82363328	0.92797409	1.1786308	0.77504127	1.0669045	1.56	0.015
Q8BIJ6	Isoleucine--tRNA ligase, mitochondrial OS	Iars2	GSkVHFVPGWDCHGLPIETK	K146	K3(42.0106),C21(57.0215)	20	1	769.3843	3	9.00408884	6.65136984	7.40694094	6.55999059	5.48900753	5.95394633	6.22676369	4.52067674	3.42271951	5.86253907	0.74	0.050
P54071	Isocitrate dehydrogenase [NADP], mitochondrial OS	Idh2	FAQTLEkVCVQTVESGAMTK	K400	K7(42.0106),C18(57.0215)	20	1	1135.0637	2	8.737091987	7.83842785	6.60750258	5.01536931	5.78502808	4.81995401	4.66243123	4.44429702	2.53166969	6.38626647	0.67	0.041
P54071	Isocitrate dehydrogenase [NADP], mitochondrial OS	Idh2	HR;TDFDKNkIWYEHR;TDFDkNk	K282	K7(42.0106)	13	2	598.625	3	94.89739489	94.1694986	81.9788744	66.0260526	70.6424209	57.5406765	52.5439776	59.5621035	33.3039722	60.2410324	0.65	0.006
P54071	Isocitrate dehydrogenase [NADP], mitochondrial OS	Idh2	HYkTDFDkNK;TDFDkNkIWYEHF	K280	K5(42.0106)	13	2	598.625	3	2.667020353	2.78535875	2.30984695	1.69304565	2.11775702	1.49467047	1.33169793	1.49308643	0.97611985	1.66511386	0.60	0.006
P54071	Isocitrate dehydrogenase [NADP], mitochondrial OS	Idh2	HYkTDFDkNK	K275	K3(42.0106),K17(42.0106)	10	2	690.3331	2	1.312671297	1.38276722	1.20673189	0.83340592	1.09028198	0.69468196	0.63615891	0.83753233	0.49417104	0.82407945	0.60	0.005
P54071	Isocitrate dehydrogenase [NADP], mitochondrial OS	Idh2	EkLILPHVDVQLK;IIWQFIKEk	K69	K2(42.0106)	13	1	525.3152	3	8.144230043	0	10.0454212	9.42978177	9.34271299	0	0	0	0	0	0.00	0.017
P46660	Alpha-internexin OS	Ina	NLQSAEEWYkSK	K288	K10(42.0106)	12	1	762.8701	2	0	0	0.47133215	0	0.5139127	0.52966667	0.54073679	0.54470028	0.42766968	0.631446	2.71	0.047
Q66JY2	INO80 complex subunit D OS	Ino80d	LQSkLAQNR	K173	K4(42.0106)	9	1	550.3145	2	0.413691139	0.41234523	0.65325261	0.44275304	0.46239279	0	0	0.44252757	0	0	0.19	0.008
P11881	Inositol 1,4,5-trisphosphate receptor type 1 OS	Itpr1	PVMLkIGTSPLK	K417	K5(42.0106)	12	1	663.3967	2	0.287711045	0	0.57616288	0.49126849	0.32679476	0	0	0	0	0	0.00	0.028
Q9Z0R4	Intersectin-1 OS	Itsn1	SGPGSQLNTkLQK	K197	K10(42.0106)	13	1	700.3806	2	0.360639775	0.39240984	0	0	0	0.62082225	0.51943813	0.63082409	0.48677631	0.60357681	3.80	0.008
Q9QXZ0	icrotubule-actin cross-linking factor 1, isoforms 1/2/3/4 (	Macf1	HLMkVR	K94	K4(42.0106)	6	1	413.2418	2	0.543850045	0.49929917	0.54484631	0.42058668	0.41080672	0	0.39791675	0.43208315	0	0	0.34	0.033
P14873	Microtubule-associated protein 1B OS	Map1b	FSPEVPGQkILHHR	K98	K9(42.0106)	14	1	562.974	3	4.243670652	4.32156764	4.58620933	4.97889635	3.85792721	2.77199399	2.91150793	4.24278095	4.05586186	2.62919757	0.76	0.032
Q8C052	Microtubule-associated protein 1S OS	Map1s	SSFWkLVR	K270	K5(42.0106)	8	1	532.7979	2	0.454185258	0.40485438	0.47765416	0.46822373	0.50952939	0	0.4117294	0.43675551	0	0	0.37	0.047
P20357	Microtubule-associated protein 2 OS	Map2	IDSQkLNFR	K1762	K5(42.0106)	9	1	581.8143	2	5.406919346	5.34057338	4.63166357	5.05204999	3.9681312	3.70537279	4.10439766	4.53764516	4.49223549	3.45738879	0.83	0.044
O09110	ıal specificity mitogen-activated protein kinase kinase 3	Map2k3	ALEHLHSkLSVIHR	K183	K8(42.0106)	14	1	841.4785	2	11.6344226	12.85219	9.50510954	9.8816614	9.37388051	7.08692338	5.61758551	7.3776331	7.72425995	5.15379587	0.62	0.002
Q8R001	licrotubule-associated protein RP/EB family member 2 (	Mapre2	FQAkLEHEYIHNFK	K108	K4(42.0106)	14	1	615.9809	3	3.030970254	2.92712673	2.99396416	3.37026121	2.37909551	1.98724927	2.2362994	2.96765981	2.33738759	2.05562416	0.79	0.030
A0A498WGS3	Myelin basic protein OS	Mbp	TTHYGSLPQk	K72	K10(42.0106)	10	0	587.2985	2	2.037530408	1.31153416	1.56805106	1.8623846	1.47379947	0	1.10888096	1.26749127	1.481226	0	0.47	0.049
Q99MR8	ylcrotonoyl-CoA carboxylase subunit alpha, mitochondr	Mccc1	EHAGkIGYPVMIK	K193	K5(42.0106)	13	1	742.9001	2	29.13908499	29.4252501	24.9052736	20.6994309	18.4399053	20.4409642	18.2614005	16.5704298	9.65105651	19.3237408	0.69	0.031
Q9D1I5	Methylmalonyl-CoA epimerase, mitochondrial OS	Mcee	SLSDEAkIGAHGkPVIFLHPK	K152	K7(42.0106),K22(42.0106)	21	2	776.7617	3	0.487552307	0.31287185	0.48916837	0.34691379	0.30821252	0.33642035	0	0	0	0	0.17	0.005
Q9D1I5	Methylmalonyl-CoA epimerase, mitochondrial OS	Mcee	SLSDEAkIGAHGkPVIFLHPK	K158	K7(42.0106),K22(42.0106)	21	2	776.7617	3	0.487552307	0.31287185	0.48916837	0.34691379	0.30821252	0.33642035	0	0	0	0	0.17	0.005
Q3UGS4	Mapk-regulated corepressor-interacting protein 1 OS	Mcrip1	VVYNGkR	K14	K6(42.0106)	7	1	439.2481	2	0	0	0.66423807	0	0.36437112	0.47167671	0.63578719	0.6334746	0.47161924	0.74478022	2.88	0.043
Q9CXD6	Mitochondrial calcium uniporter regulator 1 OS	Mcur1	TDTkLNFNLEK;TDTkLNFNLEkSF	K253	K4(42.0106),K20(42.0106)	13	2	825.4282	2	14.48058949	14.0945201	17.0577834	14.6703813	11.4626462	6.71790505	12.0129899	14.0653241	7.40025079	11.3868835	0.72	0.047
Q9CXD6	Mitochondrial calcium uniporter regulator 1 OS	Mcur1	TDTkLNFNLEkSR	K260	K4(42.0106),K20(42.0106)	13	2	825.4282	2	0.850004763	0.85591936	1.19547212	1.03792007	0.899363	0.41739828	0.82165678	0.81427229	0.49400776	0.74591452	0.68	0.022
P14152	Malate dehydrogenase, cytoplasmic OS	Mdh1	ANVkIFK	K107	K4(42.0106)	7	1	431.2633	2	20.62329627	18.589828	18.1240853	16.2739232	13.9952614	14.9909551	13.1370731	16.8093036	10.2671505	12.4581616	0.77	0.036
P08249	Malate dehydrogenase, mitochondrial OS	Mdh2	VYNPNK;kHGVYNPNkIFGVTTLI	K157	K1(42.0106),K18(42.0106)	20	2	785.7655	3	2.970161192	1.44352053	2.80206276	2.71091194	2.63273084	0.89097181	1.46940688	1.08242852	1.58737351	2.13843006	0.57	0.016
Q9Z2D6	Methyl-CpG-binding protein 2 OS	Mecp2	SPGkLVVK	K219	K4(42.0106)	8	1	435.2764	2	10.94883206	10.4584486	14.2398022	6.46883613	9.28144721	6.57383421	5.72563814	6.23214818	3.71873596	5.85775818	0.55	0.017
Q925J9	ledediator of RNA polymerase II transcription subunit 1 C	Med1	PNISPSHSRPPGGSDkLASPMK	K1206	K16(42.0106)	22	2	576.2956	4	0.408494647	0.33318874	0.66122929	0.48710179	0	0	0	0	0	0	0.00	0.026
P34884	Macrophage migration inhibitory factor OS	Mif	NYSkLLCGLLSDR	K78	K4(42.0106),C16(57.0215)	13	1	790.9086	2	34.70009533	12.936707	16.1617547	18.2403429	26.6888646	12.3233201	0	13.0251305	13.1370753	0	0.35	0.025
Q8BU88	Large ribosomal subunit protein uL22m OS	Mrpl22	NkIVYPPQLPGEPR	K50	K2(42.0106)	14	1	825.4541	2	0.531112777	0.46973709	0	0.52965254	0.48385788	0	0.4434555	0	0	0	0.22	0.049
P26041	Moesin OS	Msn	LNkDQWEER	K165	K3(42.0106)	9	1	630.3043	2	1.765045343	1.9288505	1.32459834	1.37125903	1.21103569	0.90866158	0.95889076	1.2704417	1.02658659	1.15598903	0.70	0.026
A0A6I8MWZ8	Metaxin-1 OS	Mtx1	LPSGkLQAHLR	K363	K5(42.0106)	11	1	631.3724	2	0.832073227	0.83505226	0.77534141	0.80599539	0.66404396	0.54204648	0.56446711	0.78906689	0.67181934	0.50273671	0.78	0.029
Q5SYD0	Unconventional myosin-Id OS	Myo1d	QTYEkFLHR	K634	K5(42.0106)	9	1	632.3276	2	4.05392982	3.21049698	2.10140726	2.60319258	2.11054462	1.42610466	2.0655616	1.87484354	1.38642853	1.8789315	0.61	0.039
Q9DB05	Alpha-soluble NSF attachment protein OS	Napa	CLLkVAGYAAQLEQYQK	K167	C1(57.0215),K13(42.0106)	17	1	1013.0272	2	0	0.38521827	0	0	0.38495718	0.41092081	0.43795075	0.52772017	0.42091109	0.44090934	2.91	0.034
P09405	Nucleolin OS	Ncl	ATFIkVPQNPHGK	K514	K5(42.0106)	13	1	739.9093	2	1.068305627	0.97834221	1.22637043	1.26816953	1.28682281	1.42966742	1.65277776	1.92848439	1.86947019	1.5766167	1.45	0.002
Q9Z1P6	dehydrogenase [ubiquinone] 1 alpha subcomplex subur	Ndufa7	LPVGPSHkLSNNYYCTR	K48	K8(42.0106),C24(57.0215)	17	1	1024.5045	2	7.784350074	6.87458069	6.63013771	7.19465668	6.03917594	4.08400214	6.22008431	6.50601023	4.4502926	6.00313931	0.79	0.042
Q9CR61	I dehydrogenase [ubiquinone] 1 beta subcomplex subun	Ndufb7	/DYCEHLDYVk;HEQHDWDYCEH	K97	C9(57.0215),K25(42.0106)	17	1	791.3431	3	3.556256069	3.95742636	2.65277064	2.57115902	2.14375476	1.73685877	2.11795099	2.53508957	1.41073315	2.08050001	0.66	0.039
E9PZF0	Nucleoside diphosphate kinase OS	Nme1	EHYTDLkDRPFFTGLVK	K56	K7(42.0106)	17	2	703.3652	3	1.24123465	1.04992369	0.9804747	1.10075411	0.76076973	0	0	0	0.99862976	0.82514544	0.36	0.039
E9Q5C9	Nucleolar and coiled-body phosphoprotein 1 OS	Nolc1	ANQVLkFTK	K666	K6(42.0106)	9	1	545.8164	2	0.368467344	0	0.43929463	0.41995489	0.39555378	0	0	0	0	0	0.00	0.017
P58281	Dynamin-like 120 kDa protein, mitochondrial OS	Opa1	LDAFIEALHQEk	K960	K12(42.0106)	12	0	728.3775	2	2.007669554	2.21365733	0.80997702	0.85894941	0.88629617	0	0	0.80407439	0	0.81334726	0.24	0.027
Q9D0K2	yl-CoA:3-ketoacid coenzyme A transferase 1, mitochonc	Oxct1	GNAHkIMEK	K451	K5(42.0106)	9	1	535.2766	2	21.79521432	17.767469	18.7363825	19.5214268	11.7257381	7.58183544	14.4641966	14.5905698	6.35148919	11.4828024	0.61	0.019
Q8CFI5	Probable proline--tRNA ligase, mitochondrial OS	Pars2	LGLQWMkAR	K243	K7(42.0106)	9	1	572.8184	2	0.796086104	1.37870619	1.72969391	1.68760037	0	3.01842193	1.94101353	3.18860333	3.70020691	1.62350559	2.41	0.015
P27773	Protein disulfide-isomerase A3 OS	Pdia3	YGVSGYPTLkIFR	K104	K10(42.0106)	13	1	771.9193	2	1.080239196	0.81241395	0.87342508	0.88921805	1.1807188	1.03639576	1.38426818	1.29726553	1.24160443	1.16706753	1.27	0.023
Q7TN75	Retrotransposon-derived protein PEG10 OS	Peg10	WATAkLQR	K191	K5(42.0106)	8	1	508.2878	2	0.709172712	0.73642742	0.83709808	0.85832886	0.75135278	0	0	0	0	0.64457848	0.17	0.006
Q9D0F9	Phosphoglucomutase-1 OS	Pgm1	DHWQkFGR	K419	K5(42.0106)	8	1	558.2727	2	11.88525889	12.037786	10.3091296	10.2651819	16.1697092	7.29984633	7.8489941	10.4767572	7.34021143	7.66109661	0.67	0.016
O70172	hosphatidylinositol 5-phosphate 4-kinase type-2 alpha C	Pip4k2a	FHTSYDkR	K140	K7(42.0106)	8	1	548.2645	2	0.95504564	0.94245555	0.88755834	1.07830766	0.90502271	0.63362687	0.70435092	0.69534461	0.56637703	0.91711122	0.74	0.009
P52480	Pyruvate kinase PKM OS	Pkm	LNFSHGTHEYHAETIk	K89	K16(42.0106)	16	0	642.6428	3	4.077599857	4.95366737	3.78805124	4.18847659	3.40217622	2.12486274	2.75201883	3.83051452	3.610217	2.83525167	0.74	0.032
Q76MZ3	e-protein phosphatase 2A 65 kDa regulatory subunit A a	Ppp2r1a	LVEkFGK	K472	K4(42.0106)	7	1	431.7553	2	0.387123332	0.40929426	0.31674347	0.43399853	0	0	0	0	0	0	0.00	0.018
Q6GT24	Peroxiredoxin-6 OS	Prdx6	AAkLAPEFAK	K56	K3(42.0106)	10	1	544.3109	2	7.841619892	7.4196915	6.83218196	6.66825849	4.30137727	3.00611473	4.80732416	5.95935324	3.17954097	5.75599147	0.69	0.046
P68404	Protein kinase C beta type OS	Prkcb	NLVPMDPNGLSDPYVkLK	K197	K16(42.0106)	18	1	1021.5349	2	1.412559336	0.99719935	1.3266329	1.21024825	1.3245396	1.51706518	1.89016474	1.51474179	2.15155465	1.45536694	1.36	0.025
Q99JF8	PC4 and SFRS1-interacting protein OS	Psip1	PGDLIFAkMK	K14	K8(42.0106)	10	1	581.3204	2	1.234819601	1.13567894	0.99273546	1.24862258	1.14415066	0.48179023	1.09279168	1.09371347	0.55073254	0.66361134	0.67	0.045
P26516	26S proteasome non-ATPase regulatory subunit 7 OS	Psmd7	SVVALHNLINNkIANR	K279	K12(42.0106)	16	1	909.5208	2	1.264900834	1.15093558	1.33316833	1.08878121	1.00672573	0.70270805	0.97009916	1.12504223	0.90377095	0.89580008	0.79	0.025
P26350	Prothymosin alpha OS	Ptma	VAEDDEDDDVDTKk	K104	K14(42.0106)	15	2	896.3975	2	0	0	0	0.89599887	1.09290271	0.83876365	1.36846154	1.37134382	0.87810575	1.44403701	2.97	0.030
B9EKR1	Receptor-type tyrosine-protein phosphatase zeta OS	Ptprz1	FQGWEkASLENTFIHNTGK	K87	K6(42.0106)	19	1	1125.0529	2	0.967012496	0.87884011	0.95734692	1.02366327	0.68028968	1.18021125	1.21757778	1.35529451	1.12784539	1.18492775	1.35	0.003
B9EKR1	Receptor-type tyrosine-protein phosphatase zeta OS	Ptprz1	ASkITFHWGK	K125	K3(42.0106)	10	1	608.8272	2	1.360772288	1.54729063	1.44838302	1.50424026	1.25260347	0.96342467	1.05371356	1.46281858	1.12165769	1.13630382	0.81	0.029
Q8CI94	Glycogen phosphorylase, brain form OS	Pygb	INPASMFDVHVkR	K569	K12(42.0106)	13	1	778.4061	2	1.812714634	2.01492174	2.22093415	2.10464259	1.64887472	1.63040722	1.67167871	1.68786433	0.72164593	1.43387836	0.73	0.042
Q91V41	Ras-related protein Rab-14 OS	Rab14	SCLLHQFTEkK	K34	C2(57.0215),K19(42.0106)	11	1	716.8662	2	0.568820029	0.49227447	0.66304649	0.61358004	0.67761931	0.77366075	0.8658982	0.89188944	0.78792844	0.69719709	1.33	0.003
Q8BHC1	Ras-related protein Rab-39B OS	Rab39b	LAAAYGMkYIETSAR	K148	K8(42.0106)	15	1	843.9295	2	0.518344288	0.40945717	0.49947426	0.50059209	0.42561548	0	0.46134382	0	0	0	0.20	0.013
Q80UJ7	Rab3 GTPase-activating protein catalytic subunit OS	Rab3gap1	IIHkLFVNAQR	K896	K4(42.0106)	11	1	690.9091	2	0.339892455	0.45408015	0.4416458	0.51655015	0.30232403	0	0	0.50141909	0	0	0.24	0.033
Q9JM13	Rab5 GDP/GTP exchange factor OS	Rabgef1	EFIDFLkTFHK	K147	K7(42.0106)	11	1	733.8875	2	0.382460189	0.32056422	0.4294666	0.46104769	0.44847859	0	0	0	0.43643541	0	0.21	0.019
P68040	Small ribosomal subunit protein RACK1 OS	Rack1	FSPNSSNPIIVSCGWDkLVK	K172	C13(57.0215),K26(42.0106)	20	1	1145.5804	2	1.005974649	1.06920229	0.9421736	1.14157461	0.94868863	0	0	0	0	0	0.00	0.000
Q99JI6	Ras-related protein Rap-1b OS	Rap1b	EYkLVVLGSGGVGK	K5	K3(42.0106)	14	1	724.4113	2	3.366938835	3.91408287	4.63751642	4.80658238	4.80934994	2.41177171	2.77916473	3.36988164	2.29706282	3.93083882	0.69	0.012
Q3U186	Probable arginine--tRNA ligase, mitochondrial OS	Rars2	SVLANGMkLLGITPVCR	K568	K8(42.0106),C25(57.0215)	17	1	936.0156	2	0.819302951	0.83572262	1.00462261	1.13345139	0.87959886	1.16630318	1.32443234	0.98957787	1.68450943	1.26040416	1.38	0.035
Q5SFM8	RNA-binding protein 27 OS	Rbm27	APSkLCSGSK	K800	K4(42.0106),C15(57.0215)	10	1	538.7738	2	2.283873924	1.79369657	2.87297528	0	1.64171513	0	0	0	0.95029229	0	0.11	0.030
Q148V7	RAB11-binding protein RELCH OS	Relch	TFGkIFTNTK	K871	K4(42.0106)	10	1	599.8269	2	0	0	0	0	0	0.31315895	0.50600403	0.44190275	0.48945394	0	inf	0.020
Q80XA6	RalBP1-associated Eps domain-containing protein 2 OS	Reps2	VSLSHGYSkLR	K200	K9(42.0106)	11	1	644.854	2	0.357138266	0	0.45890215	0.46433073	0.25328149	0	0	0	0	0	0.00	0.023
P28661	Septin-4 OS	Septin4	MQEMLHkIQR	K469	K7(42.0106)	10	1	678.3497	2	0.362120776	0.45343021	0.49747128	0.41940757	0.39449252	0	0	0	0	0	0.00	0.000
Q80UG5	Septin-9 OS	Septin9	LVNEkFR	K488	K5(42.0106)	7	1	474.2691	2	0.618199755	0.52993914	0.59789374	0.66927882	0.4272683	0	0	0.45848122	0.40557613	0	0.30	0.017
Q5DTL9	Sodium-driven chloride bicarbonate exchanger OS	Slc4a10	IkLFWMPAK	K960	K2(42.0106)	9	1	588.3359	2	0.331730783	0.34574284	0.53385814	0.60426934	0	0	0	0	0	0	0.00	0.026
O55042	Alpha-synuclein OS	Snca	TVEGAGNIAAATGFVkK	K96	K16(42.0106)	17	1	838.4543	2	0	0.45117387	0	1.26354105	0	0.91055874	1.02807828	1.28587813	0.73850844	1.18507023	3.00	0.047
Q924S8	Sprouty-related, EVH1 domain-containing protein 1 OS	Spred1	PTFHHWkIDDK;VTPTFHHWkIDD	K93	K9(42.0106)	14	2	598.6493	3	0.31709772	0.30395806	0.39268492	0.41932573	0	0	0	0	0	0	0.00	0.019
Q62261	Spectrin beta chain, non-erythrocytic 1 OS	Sptbn1	.AASYELHk;TQILAASYELHkFYH	K1809	K18(42.0106)	18	1	726.3723	3	9.150868966	9.44456843	11.0176077	9.79702036	8.69821035	7.78967907	7.95214081	8.81703933	7.69345198	6.29742478	0.80	0.010
Q7TNE1	Succinate--hydroxymethylglutarate CoA-transferase OS	Sugct	ELVkILSAR	K322	K4(42.0106)	9	1	535.832	2	1.456190472	1.26759818	1.54443341	1.19323186	1.12945113	0.9824098	1.20870596	1.16039704	0.76114459	1.100277	0.79	0.040
Q62277	Synaptophysin OS	Syp	LHQVYFDAPSCVk	K89	C11(57.0215)	13	0	782.3848	2	2.188152461	1.6677005	1.32079532	1.59243022	1.43608957	1.24045941	1.10721596	1.3190928	1.00194029	1.14034562	0.71	0.029
O08784	Treacle protein OS	Tcof1	TAPGPTkLGNVAPTPAK	K241	K7(42.0106)	17	1	831.4647	2	1.376466631	1.26241328	1.2630555	0.91811367	1.2259975	0.93781775	0.88865362	1.06931672	0.70919172	1.03076026	0.77	0.023
P40142	Transketolase OS	Tkt	AYGLALAkLGHASDR	K327	K8(42.0106)	15	1	792.9282	2	8.746658274	7.69767739	6.0849825	6.43913657	5.08594199	3.6923257	4.53821602	5.26991344	5.97745455	3.71099944	0.68	0.026
Q91ZE0	Trimethyllysine dioxygenase, mitochondrial OS	Tmlhe	RPENELWVkLK	K373	K9(42.0106)	11	2	727.4117	2	3.595334132	2.17191032	3.72682644	3.00939966	2.53303541	0.94735154	2.80167461	1.47112117	1.51167851	2.58425867	0.62	0.040
Q7TQD2	Tubulin polymerization-promoting protein OS	Tppp	QGGk;VDLVDESGYVPGYkHAGT	K218	K22(42.0106),K36(42.0106)	27	2	998.8208	3	0	0	0.58799488	0.58953794	0	0.92463582	1.21716958	0.89490717	0.75291728	0.82541764	3.92	0.005
Q7TQD2	Tubulin polymerization-promoting protein OS	Tppp	TVSRLTDTSkFTGSHK;LTDTSkF	K170	K6(42.0106)	12	1	682.3461	2	10.23557464	8.83072562	9.53151193	8.65810135	6.29065263	7.04733904	7.761515	6.88366397	5.56961429	6.90145792	0.78	0.047
Q7TQD2	Tubulin polymerization-promoting protein OS	Tppp	NWSkLCK	K77	K4(42.0106),C15(57.0215)	7	1	489.2473	2	4.75578436	4.50447046	4.25372409	5.00750903	5.22459454	3.3847683	3.16562123	4.57419795	3.896006	2.93782728	0.76	0.013
Q9CRB6	ıulin polymerization-promoting protein family member 3	Tppp3	NWAkLCK	K37	K4(42.0106),C15(57.0215)	7	1	481.2498	2	1.263584481	1.08266329	0.89993003	1.12767779	1.00504259	0.74294355	0.80936443	0.81255983	0.90580008	0.95290343	0.79	0.015

Q8BXJ2	Transcriptional-regulating factor 1 OS	Trerf1	kHPPIAAkVEEPLK	K639	K1(42.0106),K17(42.0106)	14	2	547.6505	3	1.180327951	1.0405716	0.8669603	0.75737232	0.80873368	0.51002731	0.61404763	0.70053982	0	0.70890542	0.54	0.030
Q8BXJ2	Transcriptional-regulating factor 1 OS	Trerf1	kHPPIAAkVEEPLK	K646	K1(42.0106),K17(42.0106)	14	2	547.6505	3	1.180327951	1.0405716	0.8669603	0.75737232	0.80873368	0.51002731	0.61404763	0.70053982	0	0.70890542	0.54	0.030
Q9JLT4	Thioredoxin reductase 2, mitochondrial OS	Txnrd2	kVkYFNIK	K151	K1(42.0106),K12(42.0106)	8	2	562.3291	2	0.358182919	0	0	0	0	0.51682032	0.64162773	0	0.52108696	0.61866369	6.42	0.027
Q91ZJ5	UTP--glucose-1-phosphate uridylyltransferase OS	Ugp2	GPSVDWGkIQRPPEDSIQPYEK	K77	K8(42.0106)	22	2	856.7624	3	0.505520824	0	0	0	0	0.56139648	0.85738117	0.84876253	0.60341614	0.78717676	7.24	0.001
P61982	14-3-3 protein gamma OS	Ywhag	AYSEAHEISk	K162	K10(42.0106)	10	0	588.7801	2	1.077998469	0.88310362	0.34942666	0.41941187	0	0	0	0	0	0	0.00	0.048
P63101	14-3-3 protein zeta/delta OS	Ywhaz	VFYLkMK	K120	K5(42.0106)	7	1	485.7751	2	78.3019421	68.9309354	66.1289697	70.2008381	58.203829	48.137405	51.9195048	64.3736352	52.2235251	51.7410558	0.79	0.009

Supplementary Table 3. Differentially abundant metabolites (DAMs) in EFV-treated vs control 5XFAD female mice as determined by metabolomics (sheet 1) and targeted quantification (sheet 2).

Compound Name	Compound class	HMDB	PubChem	KEGG	Formula	m/z	Molecular Weight	RT [min]	mzCloud Best Match	mzVault Best Match	QC_1	QC_2	QC_3	Control_1	Control_2	Control_3	Control_4	Control_5	AVE(Control)	Treated_6	Treated_7	Treated_8	Treated_9	Treated_10	AVE(Treated)	Fold change (Treated/Control)	P value
DG 36:4	Acylglycerols	-	-	-	C39 H68 O5	309.25764	308.25254	14.432	NA	42.3	5.575840573	2.146791327	3.14177232	3.319879588	1.989201211	4.776304246	2.553250636	5.518801491	3.631487434	0.526689356	0.590430516	0.546628399	0.761039466	1.04355619	0.693668785	0.19	0.002
MG 18:2	Acylglycerols	HMDB0245187	549043	-	C21 H38 O4	337.27391	336.26663	12.401	83.5	83.4	62.02620715	15.14619864	34.26919825	604.1094444	488.4070144	211.7403344	151.1131406	83.65024115	307.804035	11.5066564	18.68760524	24.68278404	31.00146837	35.93414449	24.36253171	0.08	0.023
MG 18:2	Acylglycerols	HMDB0242115	5283469	-	C21 H38 O4	355.28281	354.27553	9.671	NA	52	268.319908	17.00789901	173.8179853	274.5650475	275.7441136	276.3133546	222.0764987	235.6373345	256.8672698	144.0389805	125.6983178	186.0676184	146.2033174	106.8088673	141.7634203	0.55	0.000
MGDG 18:1_18:5	Acylglycerols	-	-	-	C45 H74 O1	820.52554	819.51826	14.255	NA	40.2	6.134342292	12.96919886	7.867061241	13.55003204	12.77030858	6.014221574	15.77666426	9.670938354	11.55643296	19.07527202	17.58287672	16.80331545	16.02922403	21.83679355	18.26549635	1.58	0.010
4-Oxoproline	Amino acids	HMDB0304793	-	-	C5 H7 N O3	129.04136	128.03408	0.851	91.6	86	1776.401971	1875.935011	1834.455421	1573.215423	2150.892833	1966.031934	1542.555913	1501.181746	1746.77557	2143.451887	2356.019862	1852.990196	2306.212813	1997.171261	2131.169204	1.22	0.044
Arginine	Amino acids	HMDB0000517	6322	C00062	C6 H14 N4 O	174.11075	173.10348	0.701	52.6	95	110.2118424	186.6005467	150.0361171	164.7342685	188.1014523	160.7482876	167.4877766	167.0665669	169.6276704	281.7679795	159.0379975	201.0927786	259.9856724	237.866097	227.950105	1.34	0.031
Glutamylleucine	Amino acids	HMDB0028823	9856500	-	11 H20 N2 O	260.13724	259.12997	3.361	NA	68.4	46.40500978	48.20398793	47.54696835	35.86136884	52.68501208	51.21121565	38.05345537	44.23621112	44.40945261	68.15097566	50.61950292	46.38602351	67.53972909	58.36524816	58.21229587	1.31	0.037
Leu	Amino acids	HMDB0000687	6106	C00123	C6 H13 N O2	131.09343	130.08615	1.441	91.5	88.8	1248.125492	1373.361515	1326.431351	1185.514576	1164.531213	1131.474358	1316.997462	1256.343941	1210.97231	1743.224293	1184.683027	1354.498598	1542.632691	1563.724653	1477.752652	1.22	0.030
Ornithine	Amino acids	HMDB0000214	6262	C00077	C5 H12 N2 O	132.08879	131.08151	0.705	NA	38.6	46.00145886	23.93822066	38.2196441	38.0627317	40.57847807	25.22751356	28.67800426	26.05474391	31.7202943	58.81087749	25.40638809	51.98070572	73.60843066	52.38033088	52.43734657	1.65	0.040
Phe	Amino acids	HMDB0000159			C9 H11 N O2	165.07803	164.07075	2.417	84.4	91.7	2942.196482	2888.548275	2964.94421	2726.52027	2844.73921	2387.088729	2647.885985	2502.350521	2621.716943	3821.127864	2726.279624	3223.175063	3750.286824	3409.947489	3386.163373	1.29	0.007
Pro	Amino acids	HMDB0251528			C5H9NO2	116.07094	115.06366	0.738	92	84.8	177.02097	183.9255406	172.7550239	109.1783813	48.90396006	130.5386815	131.0016204	132.3298385	110.3904964	211.1539153	146.8932029	163.4228747	257.7938283	228.1265792	201.4780801	1.83	0.008
Sarcosine	Amino acids	HMDB0000271	1088	C00213	C3 H7 N O2	90.05563	89.04835	2.882	NA	56.4	118.0761036	123.5338097	126.9209832	128.7460297	148.0198279	95.72134289	97.45003895	98.28829207	113.6451063	131.1043446	128.2917935	170.3684862	167.0833626	150.1856638	149.4067301	1.31	0.031
Taurine	Amino acids	HMDB0000251	1123	C00245	C2 H7 N O3	126.02208	125.0147	0.756	62	52.3	649.1224421	617.835636	653.4936007	511.4300679	685.9657782	797.4138559	780.7392365	901.965339	735.5028555	608.8193092	532.729345	497.4483308	574.4684578	588.0237004	560.2978286	0.76	0.034
Trp	Amino acids	HMDB0000929	6305	C00078	11 H12 N2 O	188.07067	204.08994	3.267	95	89.5	217.66169	223.916257	216.0094172	201.8515242	195.963131	168.0646557	212.9367727	177.6324131	191.2896993	277.3086876	207.6875144	217.9574001	282.2399124	270.841248	251.2069525	1.31	0.010
Tyr	Amino acids	HMDB0000158			$C_9H_{11}NO_3$	182.08139	181.07409	1.312	98.7	96.9	72.22433534	74.54661752	77.11874365	69.02459956	68.20582342	68.33467661	78.10015479	77.52814629	72.23868013	102.3051613	80.89222542	87.26910541	98.71998701	88.71541678	91.58037918	1.27	0.003
NAE 18:0	NAE 18:0	HMDB0013078	27902	-	C20 H41 N O	310.31047	309.30319	10.782	94.2	88.2	100.3919482	95.25659743	100.3081868	109.7668328	102.84009	70.1520215	64.11084327	73.0834997	83.99065745	124.1487339	72.86596137	122.4278746	145.5789504	136.9282088	120.3899458	1.43	0.049
NAE 20:0	NAE 20:0	-	-	-	C22 H45 N O	356.35255	355.34527	9.04	NA	94.4	142.7047269	145.6207122	125.6748199	151.4804558	131.0205475	154.2712757	159.3452654	143.5726475	147.9380384	129.1270314	144.6908936	78.09541187	100.6138367	136.081764	117.7217875	0.80	0.053
CAR 18:0	Carnitines	HMDB0000848	52922056	-	C25 H49 N O	428.37366	427.36638	10.27	NA	90	251.5455332	296.7115252	367.3693554	82.867948	122.7992473	178.9458575	140.0993569	174.1935193	139.7811858	427.6833027	388.3964852	580.7222074	537.8992818	549.8616731	496.91259	3.55	0.000
CAR 20:1	Carnitines	-	-	-	C27 H52 N O	454.3891	453.38182	10.244	NA	91.7	200.1662694	200.7298518	211.4514587	193.9365079	196.0394037	240.7037091	116.9284595	120.5708103	173.6357781	242.767896	174.9736404	309.4716979	323.084012	329.6525233	275.9899539	1.59	0.028
FA 8:0	Fatty acids	HMDB0000482	379	C06423	C8 H16 O2	144.11388	143.10661	14.457	83.1	86.2	170.9250203	131.7035457	168.3413588	139.6011078	134.0942965	155.6848608	148.4473256	137.2796511	143.0214484	154.5401483	188.3009399	181.288285	212.5279908	198.1253716	186.9565471	1.31	0.003
FA 9:0	Fatty acids	HMDB0000847	8158	C01601	C9 H18 O2	158.12964	157.12236	14.457	NA	38.9	102.5663837	87.35329457	95.43518212	79.27822501	79.18282768	81.83224124	82.4133835	74.87090035	79.51551556	90.67074551	103.1298068	88.46365032	100.0437319	99.5167598	96.36493887	1.21	0.001
FA 10:0	Fatty acids	HMDB0000511	2969	C01571	C10 H20 O2	172.1454	171.13812	14.458	66.7	76	259.5822945	242.9784175	253.8082669	228.5032475	199.8291719	240.8218108	220.217634	186.7600996	215.2263928	249.8357017	255.5639212	255.6356357	303.626206	298.2050424	272.5733014	1.27	0.005
FA 17:0	Fatty acids	HMDB0002259	10465	-	C17 H34 O2	270.25591	269.24864	14.318	NA	96.7	50.60565597	46.1610469	49.0062267	40.4681632	52.60953399	51.69457813	30.3611465	36.52823276	42.33233092	60.24984365	50.61618564	51.15286492	68.88697979	49.88522945	56.15822069	1.33	0.041
FA 20:0	Fatty acids	HMDB0002212	10467	C06425	C20 H40 O2	330.33684	329.32957	8.726	NA	78.9	541.9233017	549.3202978	554.5308617	604.9565437	517.1113736	605.6224496	566.3924803	538.4161262	566.4997947	359.865469	540.4331459	375.0811566	506.5101165	371.0281417	430.5836059	0.76	0.012
FA 18:1	Fatty acids	HMDB0000207	445639	C00712	C18 H34 O2	282.25571	281.24843	13.802	98.4	99.7	4578.578398	3977.699832	5144.313558	3712.06335	4370.631636	4025.719426	3921.876753	3790.179158	3964.094065	5096.092997	5723.440462	4202.248533	5990.342069	4764.868488	5155.39851	1.30	0.008
FA 18:2	Fatty acids	-	-	-	C18 H32 O2	263.23705	262.22978	12.414	60.2	61.4	21.22115412	12.59288462	12.67103374	82.33835205	62.13566146	49.85441639	19.11056374	12.222333	45.13226533	9.56077923	3.983981062	12.32767474	14.73298808	12.69744759	10.66057414	0.24	0.032
FA 22:6	Fatty acids	HMDB0002183	445580	C06429	C22 H32 O2	329.24757	328.24029	12.67	79.8	76.9	39.46027731	32.94604503	30.09255695	45.46163325	38.26268089	52.59254322	35.62315679	39.61715192	42.31143321	40.05300548	29.66645714	33.07877372	35.93802194	28.17163282	33.38157822	0.79	0.043
FA 24:6	Fatty acids	-	-	-	C24 H36 O2	356.27157	355.26429	13.267	NA	88.1	87.40892346	73.36603699	85.43980556	64.87981256	81.27901058	71.70524694	50.29169022	91.26589451	71.88433096	126.2671793	101.5417321	86.07073138	113.3528372	83.42896977	102.13229	1.42	0.022
3-Hydroxybutyric acid	Ketone bodies	HMDB0000011	441	C01089	C4 H8 O3	104.04605	103.03878	1.509	NA	85.4	165.8014167	165.5799924	166.5037898	53.62150205	170.6332536	177.5104281	186.3691526	133.369276	144.3007225	195.2669601	166.6744974	228.3123111	237.893196	204.019641	206.4333211	1.43	0.054
Acetoacetic acid	Ketone bodies	HMDB0000060	96	C00164	C4 H6 O3	103.03943	102.03217	0.754	NA	34	564.9309505	613.8481283	607.4605547	406.1004468	494.7984307	539.9665648	577.2065455	565.6670834	516.7478142	553.0157856	634.3236409	730.0509325	850.3178434	738.1822105	701.1780826	1.36	0.014
Octadecanamide	Glycerophospholipids	HMDB0034146	31292	C13846	C18 H37 N O	284.29509	283.28782	7.935	NA	71.2	148.6122412	238.2980207	146.7496897	236.5254085	227.6667559	262.475326	252.5021126	245.814169	244.9967544	123.6005883	241.2447561	135.2985655	140.1882312	133.9238196	154.8511921	0.63	0.004
LPC 18:0	Glycerophospholipids	-	-	-	26 H54 N O7	546.35292	545.34564	11.466	NA	92.5	342.1137669	320.8960252	273.8048931	607.9131323	636.1956854	752.8928479	593.1590347	390.8395542	596.2000509	269.2650429	226.2554001	223.2421526	211.0421985	223.6265551	230.6862698	0.39	0.000
LPC 18:1	Glycerophospholipids	-	-	-	26 H52 N O7	522.35543	521.34815	10.39	NA	88.5	4250.655279	3947.508107	4487.127043	3767.264941	3251.388642	4759.036666	3659.29441	4378.377282	3963.072388	5164.687722	4825.390216	4870.900464	5103.736528	5008.312531	4994.605492	1.26	0.006
LPC 22:6	Glycerophospholipids	-	-	-	30 H50 N O7	568.33953	567.33228	9.539	NA	93.2	1560.269969	1577.24288	1723.835598	1895.627778	1290.58755	1292.852465	1009.223171	1368.339314	1371.326056	1503.126457	1570.510246	3086.209867	2428.041219	2287.786244	2175.134807	1.59	0.040
LPC O-16:1	Glycerophospholipids	-	-	-	24 H50 N O6	480.34515	479.33787	10.55	NA	94.2	109.9173007	105.4108863	116.6709682	109.8016316	118.1706094	74.44509641	65.74408228	27.27779229	79.0878424	127.1052431	68.93858493	144.4125562	198.3061188	175.1371211	142.7799248	1.81	0.050
LPC O-16:2	Glycerophospholipids	-	-	-	24 H48 N O6	478.32935	477.32207	10.093	NA	70.1	1140.945035	1270.335423	1561.900626	504.2618672	493.7248392	935.4126307	747.5592935	1586.258551	853.4434363	2170.288093	1907.554299	2332.666296	2532.138121	2073.209427	2203.171247	2.58	0.000
LPC O-18:2	Glycerophospholipids	-	-	-	26 H52 N O6	506.36088	505.3536	10.854	NA	81.6	29.72826844	53.86644104	56.6807726	55.12850869	65.29506552	27.84992811	25.97672657	33.32987193	41.51602016	122.4054844	50.27940023	95.13450667	112.9743382	98.62348047	95.88344199	2.31	0.006
LPC O-18:3	Glycerophospholipids	-	-	-	26 H50 N O6	504.34491	503.33763	10.444	NA	64.7	324.7791269	361.3492906	450.0420945	176.9669519	164.9845371	231.9342074	185.1183252	304.9622347	212.7932513	456.8456132	473.7064594	658.307542	492.5781541	576.743063	531.6361663	2.50	0.000
LPE 18:1	Glycerophospholipids	-	-	-	23 H46 N O7	479.30105	478.29378	10.321	NA	88.7	3231.210231	2893.13414	3268.661685	3304.421841	2942.830394	2386.506713	2254.513642	2592.400501	2696.134618	3244.992394	2703.625526	3719.762037	4003.810674	3457.912385	3426.020603	1.27	0.037
LPI 18:0	Glycerophospholipids	-	-	-	27 H53 O12	600.32754	599.32027	14.441	NA	92.7	629.3677218	837.7058517	701.9442845	223.6871394	202.736721	310.5226886	488.5288321	432.0715664	331.5093895	526.4281827	1092.398946	1092.135726	666.1838096	651.2795997	805.6852528	2.43	0.007
LPS 18:0	Glycerophospholipids	-	-	-	24 H48 N O9	525.30668	524.2994	9.936	NA	92.8	732.6863121	503.5348092	546.0654086	530.0635092	574.2477926	602.8034831	664.9147562	676.0745522	609.6208187	453.736875	421.4750303	443.7298195	482.5572109	296.218804	419.5435479	0.69	0.002
PG 34:1	Glycerophospholipids	-	-	-	40 H77 O10	748.52705	747.51978	11.714	NA	65	3.308362735	5.475586494	27.88082516	10.63707058	6.546842937	1.238542379	2.93107165	8.645520427	5.999809595	15.07133267	21.61167733	22.51716258	27.51995068	37.62955365	24.86993538	4.15	0.002
PG 36:2	Glycerophospholipids	-	-	-	42 H79 O10	774.54173	773.53445	14.309	NA	83.5	13.72433997	16.49383565	15.04372413	20.24412612	17.10772505	10.24129715	12.59890269	8.813793098	13.80116882	18.76954137	27.26768605	17.39413648	16.35306261	32.19916421	22.39671814	1.62	0.053
PG 38:4	Glycerophospholipids	-	-	-	44 H79 O10	798.54144	797.53416	14.104	NA	60.1	0.290750553	7.826565867	6.32956976	0.271270604	0.274276931	0.292410351	0.288865221	0.304420545	0.28624873	16.47118419	19.03507162	22.7492443	7.140247713	13.2766965	15.73448886	54.97	0.000
PG 44:12	Glycerophospholipids	-	-	-	50 H75 O10	866.50995	865.50267	13.803	NA	51.3	90.86968018	83.21927841	81.00113903	7.922825625	2.952521782	75.4683211	60.37932201	79.82786918	45.31017194	185.6085089	138.9081298	110.7583509	86.19464778	57.5672428	115.807376	2.56	0.034
PG 16:0_16:0	Glycerophospholipids	HMDB0010570	446440	-	38 H75 O10	722.51018	721.5029	14.439	NA	53.2	0.762035618	2.574975503	3.597776117	3.347839397	3.77985733	0.770876367	1.110200564	0.971193981	1.995993528	5.327261679	19.05603574	9.075570989	5.939895524	6.111101763	9.101973139	4.56	0.028
PG 16:0_16:0	Glycerophospholipids	-	-	-	38 H75 O10	722.51044	721.50316	13.805	NA	83.7	0.729375009	1.656728596	4.345735312	3.74905975	2.647281159	0.647809905	0.523258774	0.836489237	1.680779765	5.602688855	10.12750859	7.143121521	8.315610752	6.865215724	7.610829088	4.53	0.000
PG 16:0_18:1	Glycerophospholipids	-	-	-	40 H77 O10	748.52614	747.51887	14.441	NA	82.3	5.941025556	9.729464286	23.61211122	21.42868333	1.847175902	2.854388926	6.526098428	5.336153354	7.598499988	17.25272645	101.197224	63.68032229	47.80332436	50.2748704	56.0416935	7.38	0.009
PG 16:0_20:4	Glycerophospholipids	-	-	-	42 H75 O10	770.5099	769.50262	13.615	NA	77.7	10.92948549	6.193738559	6.590684925	12.26588174	11.91835556	11.42673027	9.191878665	12.96632372	11.55383399	7.328025945	6.876263009	7.653602099	12.64522248	3.803272829	7.661277272	0.66	0.037
PG 18:0_20:4	Glycerophospholipids	-	-	-	44 H79 O10	798.54091	797.53364	13.621	NA	83	0.958556138	2.269303064	4.396196496	0.220206134	0.20241944	0.256029594	0.245914848	0.196361786	0.22418636	7.09877162	7.453639587	6.948866786	7.086896984	7.897618686	7.297158733	32.55	0.000
PC 15:0_15:0	Glycerophospholipids	-	-	-	38 H76 N O8	706.53711	705.52984	13.662	NA	31.4	5.182700036	35.17051312	31.97271171	0.50468551	0.528506634	0.588076862	3.128141313	29.4915429	6.848190644	23.57400025	29.03757744	21.4976039	18.47759526	15.50011993	21.61737936	3.16	0.043
PC 16:0_16:0	Glycerophospholipids	HMDB0245715	6138	-	40 H80 N O8	734.56786	733.56056	14.426	95.7	77.6	0.356123169	344.3887384	193.9675673	0.124916631	0.192268981	0.1360516	0.226160824	0.150422052	0.165964018	157.9614412	274.8403872	258.4755229	323.2750303	688.1567654	340.5418294	2051.90	0.006
PC 16:0_16:1	Glycerophospholipids	-	-	-	40 H78 N O8	732.55255	731.5452	12.389	NA	73.3	166.1875654	166.127508	140.9983041	0.800673625	0.79682199	0.882260643	0.8284573	111.8705261	23.03574793	138.200614	141.4845779	133.9333817	109.8529039	124.3488485	129.5640652	5.62	0.002
PC 16:0_18:1	Glycerophospholipids	-	-	-	42 H82 N O8	760.5831	759.57583	12.416	NA	82.5	0.717232177	0.718440424	753.6780292	1.966523776	2.911267372	1.848235509	3.017294349	2.360035269	2.420671255	1014.768922	1335.698017	1325.363344	1375.818144	1807.69816	1371.869317	566.73	0.000
PC 17:1_17:1	Glycerophospholipids	-	-	-	42 H80 N O8	758.5678	757.56059	13.678	NA	51.4	187.6379624	37.72092075	120.6460837	0.820398537	0.845749052	0.793487313	1.071231715	41.3560956	8.977392443	65.58970867	85.42525444	68.66578486	56.45644796	54.96281864	66.22000291	7.38	0.000
PC 18:0_20:4	Glycerophospholipids	-	-	-	46 H84 N O8	810.59875	809.5926	13.465	NA	59	1.870056628	44.28260577	65.83031883	1.774154727	1.857894718	2.067305924	1.941020014	1.945100654	1.917095207	114.7948278	128.9672766	115.7079597	139.1186676	234.2504294	146.5678322	76.45	0.000
PC 18:1_18:1	Glycerophospholipids	-	-	-	44 H84 N O8	786.5989	785.59162	13.642	NA	48.4	1.688353845	1.61235802	55.51713205	1.601770188	1.677373639	1.866437548	1.752422123	1.75610627	1.730821954	34.26098292	94.64055011	99.1258488	23.2763869	146.0523092	79.47121559	45.92	0.009
PC 18:2_18:2	Glycerophospholipids	-	-	-	44 H80 N O8	782.56753	781.56031	14.369	NA	71.3	216.5243199	662.6417728	460.7280144	3.003150576	3.216192211	3.174695524	135.4930534	173.2453608	63.6264905	389.6599323	608.3017338	186.9926148	413.4412759	739.8143806	467.6419875	7.35	0.004
PC 19:2_19:2	Glycerophospholipids	-	-	-	46 H84 N O8	810.59821	809.59093	14.425	NA	81.4	0.450215807	79.14729322	67.13898747	0.157921322	0.24306909	0.282371404	0.285915624	0.299151506	0.253685789	54.83549556	109.9449517	75.08487823	7.751945845	217.2368612	92.97082651	366.48	0.030
PC 20:3_20:3	Glycerophospholipids	-	-	-	48 H84 N O8	834.59821	833.59093	13.662	NA	64.2	2.038622417	268.98772	67.10890887	1.934075976	2.02536424	2.253651647	2.115982401	2.120430867	2.089901026	181.8429056	188.974627	146.926199	117.8336377	107.7309574	148.6616653	71.13	0.000
PC 20:3_20:4	Glycerophospholipids	-	-	-	48 H82 N O8	832.58276	831.57549	12.663	NA	69.9	0.878548301	82.34633754	43.30704301	0.834747787	0.798789034	0.949515015	0.903308248	0.917136105	0.880699238	79.60709982	87.5771907	78.23394441	53.40203742	66.76994252	73.11804297	83.02	0.000
PC 32:0	Glycerophospholipids	-	-	-	40 H80 N O8	756.54963	755.54236	12.445	NA	54.4	2.11859542	2.376555086	12.60038508	0.571526049	0.562309284	1.131435903	13.85745853	8.358000419	4.896146037	45.20770376	25.77820778	29.75314953	30.01494397	34.58588933	33.06797887	6.75	0.000
PC 40:6	Glycerophospholipids	-	-	-	48 H84 N O8	834.59857	833.5913	13.314	NA	74.8	0.645618856	279.6214732	60.27988042	0.62067988	0.617615993	0.71210064	0.663382061	0.65702144	0.654160003	211.3991186	190.6270622	135.2213579	111.3219567	105.2304521	150.7599895	230.46	0.000
PE 16:0_22:6	Glycerophospholipids	-	-	-	43 H74 N O8	763.51569	762.50841	12.017	NA	89.8	317.3980682	232.9576832	354.329999	85.209963	7.520183761	83.44882958	91.40700422	279.3168011	109.3805563	368.287609	200.4874591	373.9331681	221.9496493	305.8334833	294.0982738	2.69	0.013
PE 38:6	Glycerophospholipids	-	-	-	43 H74 N O8	763.51539	762.50811	12.313	NA	80.9	147.5313517	131.7934001	167.6168194	16.57830598	8.125098896	115.4204826	133.0661884	99.76613326	74.59124183	193.6740657	198.302278	168.4122945	68.62241169	156.0986742	157.0219448	2.11	0.046
PE 16:1_22:6; O	Glycerophospholipids	-	-	-	43 H74 N O7	747.52114	746.51387	12.291	NA	83	205.9707161	56.75931616	167.0848473	170.6046044	123.8857362	33.82879717	43.85086731	80.58476962	90.55095494	218.9757224	228.1629633	200.9437875	236.4304952	216.5288232	220.2083583	2.43	0.001
PI 18:0_20:4	Glycerophospholipids	-	-	-	C47 H83 O13	886.55763	885.55035	14.239	NA	78.8	210.4560132	113.8237462	238.3041614	544.5844742	431.0261055	220.7150968	221.7018847	236.7674437	330.959001	206.7509298	164.3729091	54.94305095	47.24173934	154.5637719	125.5744802	0.38	0.024
PI 38:4	Glycerophospholipids	-	-	-	C47 H83 O13	886.5581	885.55082	5.92	NA	88.9	120.3854136	114.7319752	94.45409169	212.2245471	164.4730644	152.4790709	134.1595291	128.8530677	158.4378558	121.5149672	113.3793465	100.9916966	67.19869485	31.97563432	87.01206789	0.55	0.013
PS 40:6	Glycerophospholipids	-	-	-	46 H78 N O1	835.5365	834.52922	14.442	NA	88.8	1311.365538	1031.703771	937.5894059	1937.817609	1402.576239	1102.081761	1219.914544	1288.389193	1390.155869	807.5985894	1109.899285	1004.396828	796.969797	731.7002508	890.11295	0.64	0.015
Phosphorylcholine	Phosphorylcholine	HMDB0001565	1014	C00588	5 H14 N O4	184.07347	183.06619	9.53	NA	95.9	5513.192222	3026.176237	6574.617626	4718.991531	2494.635477	1348.765062	1157.039311	3124.710256	2568.828327	6570.815893	6019.421924	5617.017119	7173.596182	7539.552906	6584.080805	2.56	0.001
13,14-dihydro-15-keto PGA	Prostaglandins	-	-	-	C20 H30 O4	357.20894	356.20159	12.433	37.1	53	14.10409439	12.85270459	9.015398996	15.79214095	16.31814172	20.56690763	17.7781043	14.65228192	17.0215153	10.82568809	11.01104699	8.790956326	7.824917957	7.17823057	9.126167987	0.54	0.000
8-*iso* $PGF_{2\alpha}$ Ethanolamide	Prostaglandins	-	-	-	C22 H39 N O	415.3042	414.29692	13.748	NA	31.7	1.832878417	1.750377283	1.807888787	19.26667127	19.43831764	12.64421187	2.864489925	1.906430509	11.22402424	1.811503797	1.778087012	1.762549221	1.783483264	1.860225853	1.799169829	0.16	0.039
PGF3 alpha	Prostaglandins	HMDB0002122	5280940	C06476	C20 H32 O5	398.23364	397.22636	13.838	NA	49.7	35.76524536	34.9360585	38.41858745	10.53979565	32.93743632	9.683371336	18.30729657	23.8357059	19.06072116	51.40674927	20.45687526	35.21899092	49.73796357	39.58015776	39.28014736	2.06	0.021
SM d18:1_18:0	Spingolipids				C41 H83 N2 O	731.605	730.59765	14.427	NA	84.2	130.2626996	147.8987969	111.1315316	0.141438469	0.217699038	0.252899219	0.256073515	72.27174829	14.62797171	66.09149538	81.2938954	65.35883642	84.60165347	123.6620664	84.20158941	5.76	0.005
CerP 14:1_10:0; O2	Spingolipids	-	-	-	24 H48 N O6	478.32946	477.32218	11.816	NA	43.8	107.063378	206.8528951	129.1969501	2.259492954	2.455404875	3.175414248	34.52121416	145.9043555	37.66317635	281.526866	302.4126006	217.1875478	196.8561304	172.8179655	234.1602221	6.22	0.001
Cholest-4-en-3-one	Sterols	-	-	-	C27 H44 O	385.3467	384.33943	12.151	82.3	16.64648181	4.718316223	9.744871975	3851.48506	3166.790917	1596.356164	245.2861733	72.14718176	1786.413099	2.630478029	2.562519252	2.596652858	3.011254995	2.928696171	2.745920261	0.001537114	0.05	0.047
Cholic acid	Sterols	HMDB0000619	221493	C00695	C24 H40 O5	355.26361	354.25634	11.797	32.9	85.83740428	98.53036926	48.73729499	192.3214417	138.6067405	107.4013525	76.62578565	82.59075303	119.5092147	58.2918756	46.0528326	19.31049596	9.935757	9.947691093	28.70773045	0.240213531	0.00	0.005
Deoxycholic acid	Sterols	HMDB0000626	222528	C04483	C24 H40 O4	392.29281	391.28554	9.576	98.3	95.3	116.3713269	120.4946477	104.5461783	141.2737899	144.8159567	130.9943867	123.4325992	128.0288156	133.7091096	106.2514592	103.2124359	20.84289495	104.043096	99.76208277	86.82239376	0.65	0.025
Lithocholic acid	Sterols	HMDB0000761	9903	C03990	C24 H40 O3	422.30333	421.29605	11.322	95.9	84.4	34.73073488	41.79923894	36.06291169	45.73856561	46.62365573	39.34163854	50.59369302	40.22119622	44.50374982	29.07128115	28.77267309	25.01100254	34.88442244	26.46468841	28.84081353	0.65	0.000
Solanidine	Sterols	HMDB0003236	65727	C06543	C27 H43 N	398.34213	397.33485	6.47	96	83.7	13.33483266	11.98999885	11.29203863	35.35096327	24.50952372	22.80806581	17.88733162	15.84531631	23.28024015	11.15640213	10.42158625	9.765653373	9.599844745	6.785032104	9.54570372	0.41	0.004
minocyclopropanecarboxylic	nspecified Organic compoun	HMDB0036458	535	C01234	C4 H7 N O2	102.05542	101.04825	0.831	NA	65.6	539.8622179	586.336233	586.9812639	427.9109057	475.1660945	584.4928431	515.6694356	517.0436613	504.056588	631.1589198	545.8530283	620.5477026	808.7985704	747.5134272	670.7743297	1.33	0.015
1,2-Cyclohexanediol	nspecified Organic compoun	HMDB0244061	13601	-	C6 H12 O2	116.08249	115.07521	14.463	NA	61.2	46.84404707	38.3266414	46.59566415	41.24938693	38.8157981	41.8546023	38.6617778	40.86682981	40.28967899	46.60462611	59.68363572	55.33822648	66.31726208	53.53349918	56.29544991	1.40	0.001
1,5-Naphthalenediamine	nspecified Organic compoun	HMDB0244231	16720	C19463	C10 H10 N2	159.09169	158.08441	3.264	NA	75.8	124.4834916	114.3733398	119.7450988	115.8177221	119.6446136	104.1929948	115.2588835	100.7725052	111.1373438	143.414382	118.6036647	115.1724896	142.2691646	168.7839923	137.6487386	1.24	0.034
,2'-(Tetradecylimino)diethan	nspecified Organic compoun	-	-	-	C18 H39 N O	302.30554	301.29827	7.904	NA	93.9	581.5288663	577.0514642	568.7302282	667.691325	550.8304168	634.8651232	631.8342951	576.1992045	612.2840729	500.1428202	583.7871636	542.1791326	548.9852615	376.8129009	510.3814558	0.83	0.041
2,6-Di-tert-butylbenzoquinon	nspecified Organic compoun	HMDB0013817	12867	-	C14 H20 O2	221.15385	220.14658	14.419	NA	31.9	1.659063548	0.52795438	1.131291929	3.325294987	4.459388802	3.65978651	1.732855052	3.550187937	3.345502658	0.367804334	1.202657725	0.267262541	0.53145865	0.728749282	0.619586506	0.19	0.000
3-Pyridinol	nspecified Organic compoun	-	-	-	C5 H5 N O	97.02895	113.04816	1.251	NA	45.8	30.24611093	31.07289781	34.81141311	37.97848664	34.54851125	39.03432031	44.12834879	41.78719966	39.49537333	32.35613454	31.20364192	28.90650545	29.97546202	35.16749035	31.52184686	0.80	0.004
4-Dihydroxyhydrocinnamic a	nspecified Organic compoun	HMDB0000423	348154	C10447	C9 H10 O4	182.08133	164.04747	1.314	81	83.6	72.22433534	74.54661752	77.11874365	69.02459956	68.20582342	68.33467661	78.10015479	77.52814629	72.23868013	102.3051613	80.89222542	87.26910541	98.71998701	88.71541678	91.58037918	1.27	0.003
4-Hydroxybenzaldehyde	nspecified Organic compoun	HMDB0011718	126	C00633	C7 H6 O2	123.04435	122.03707	1.306	90.4	85.3	640.4100091	630.9174049	663.4216347	535.5356702	532.8259662	592.2611692	692.3247717	634.2431759	597.4381506	879.5232224	687.263769	676.3710895	793.0316572	747.0061848	756.6391846	1.27	0.011
4-Hydroxyquinoline	nspecified Organic compoun	HMDB0246466	-	C06343	C9 H7 N O	146.06007	145.05279	3.265	48.2	91.3	1283.332699	1278.197289	1289.00527	1282.355537	1208.936454	1095.433241	1218.842037	1147.110803	1190.535614	1690.725639	1257.14906	1278.926458	1632.447976	1685.039651	1508.857757	1.27	0.016
4-Methoxycinnamic acid	nspecified Organic compoun	HMDB0002040	699414	-	C10 H10 O3	161.05971	160.05247	4.132	59.2	68.3	140.7026163	11.7087343	75.71435634	17.14283472	19.13126244	13.84365498	12.88613473	12.20844234	15.04246584	11.25927842	10.70264307	11.30470001	9.92420976	11.28382599	10.89493145	0.72	0.016
4-Phenylbutyric acid	nspecified Organic compoun	HMDB0000543	4775	-	C10 H12 O2	165.09122	164.08394	11.863	96.5	87.7	105.2314238	71.04553427	72.27618739	280.1686116	116.3998021	202.9368408	277.8343531	302.9012706	236.0481756	34.09072865	31.01533593	39.96022076	40.37202424	32.88648383	35.66495868	0.15	0.000
4-Piperidinecarboxamide	nspecified Organic compoun	-	-	-	C6 H12 N2	129.10251	128.09523	4.314	NA	33.5	408.9765822	374.5216822	371.3211558	491.1661999	423.1386315	537.0552643	490.4062249	456.164042	479.5860725	439.9977942	356.3365378	337.303816	374.5671486	386.0266461	378.8463885	0.79	0.005
4'-Methoxyacetophenone	nspecified Organic compoun	HMDB0032570	7476	-	C9 H10 O2	151.07542	150.06815	13.377	78.1	85.3	6.199737505	4.925203742	3.883468829	108.3515998	106.5840787	35.10068767	13.82835571	8.6233129	54.49760696	3.053461707	2.546682308	2.584525148	1.883047274	1.880331098	2.389609507	0.04	0.046
Anagrelide	nspecified Organic compoun	HMDB0014406	2182	-	10 H7 Cl2 N3	256.00326	254.996	10.16	NA	55.2	162.5962213	143.4473694	106.424986	121.7683288	133.576016	258.1129885	222.0794246	208.8274441	188.8728404	139.6209168	107.0660012	91.20561602	70.16143282	33.35549813	88.28189299	0.47	0.013
Anserine	nspecified Organic compoun	HMDB0000194	112072	C01262	10 H16 N4	240.12202	239.11474	0.65	32.4	86.2	241.1716629	257.0955583	258.7593077	252.5949354	204.004514	194.075335	223.4535973	251.8474849	225.1951733	369.3891618	200.2649519	321.508073	417.7092039	269.3205128	315.6383807	1.40	0.053
Apocynin	nspecified Organic compoun	HMDB0247918	2214	C11380	C9 H10 O3	167.07042	166.06315	13.713	42.4	46.8	1.122570683	0.933774612	0.862646611	20.95394258	32.8059195	14.77978554	5.541066261	1.488393577	15.11382149	0.638783116	0.626999493	0.621520465	0.613605987	0.648816489	0.62994511	0.04	0.032
Benzalacetone	nspecified Organic compoun	HMDB0031617	637759	-	C10 H10 O	165.09123	164.08394	11.14	NA	47.5	80.07977715	59.78709564	60.07740302	112.8306133	94.34727418	304.7024671	482.3297003	273.7429736	253.5906057	46.83313574	44.09595638	42.46905221	40.43885809	41.15535273	42.99847103	0.17	0.018
Choline	nspecified Organic compoun	HMDB0000097	305	C00114	C5 H13 N O	104.10745	103.10019	0.735	98.5	6948.916491	7376.589514	7217.39401	5433.02293	5665.719723	7469.372127	7215.383893	6991.528689	6555.005472	7867.23334	7746.423481	7748.192461	7763.848595	7907.768881	7806.693352	1.190951462	0.02	0.018
Cuminaldehyde	nspecified Organic compoun	HMDB0002214	326	C06577	C10 H12 O	149.0962	148.08895	11.693	NA	79.1	14.14898035	16.75874289	13.88995027	27.96071803	24.05806461	24.44546721	21.05780912	22.15158257	23.93472831	13.62695152	14.15674174	13.77431639	14.88343834	14.72730984	14.23375157	0.59	0.000
Cyclopentanone	nspecified Organic compoun	HMDB0031407	8452	C00557	C5 H8 O	84.08147	101.08475	0.655	NA	32.1	996.0183258	948.553566	1023.241101	699.0043857	798.4298006	957.6324772	987.2585602	976.932794	883.8516035	1300.165892	980.0939154	1016.736479	1179.153394	1288.978765	1153.025689	1.30	0.016
Dihydrothymine	nspecified Organic compoun	HMDB0000079	676414	C00906	C5 H8 N2 O2	129.06616	128.05889	3.576	NA	63.9	123.3698089	119.7142624	73.66155633	86.23080795	99.40921007	116.4130755	106.7227384	120.301751	105.8155166	134.7518775	120.0740374	135.6500807	125.245966	120.1754226	127.1794768	1.20	0.016
Glutamyltyrosine	nspecified Organic compoun	HMDB0028831	515717	-	14 H18 N2	310.11683	309.10956	2.713	NA	68	10.31521568	10.59914074	10.07454873	6.220123628	9.538685416	8.778214208	6.729246594	8.86447821	8.026149611	15.78869992	10.36470946	9.417264853	16.51009499	14.11342416	13.23883868	1.65	0.011
Hypoxanthine	nspecified Organic compoun	HMDB0000157	790	C00262	C5 H4 N4 O	136.0373	135.03002	1.245	98.8	99.2	2387.734119	2534.087808	2584.555622	1959.269359	2585.156462	2511.282322	2076.93243	2271.008484	2280.729811	2683.306641	2372.997332	2456.603075	3411.639156	3146.607351	2814.230711	1.23	0.052
Indoleacrylic acid	nspecified Organic compoun	HMDB0000734	5375048	-	C11 H9 N O2	188.07063	187.06333	3.263	95.1	92.6	217.66169	223.916257	216.0094172	201.8515242	195.963131	168.0646557	212.9367727	177.6324131	191.2896993	277.3086876	207.6875144	217.9574001	282.2399124	270.841248	251.2069525	1.31	0.010
Indolelactic acid	nspecified Organic compoun	HMDB0000671	676157	C02043	C11 H11 N O	205.07338	204.0661	5.072	66.3	96.4	16.88942663	17.92985345	17.68617481	14.76262367	14.75046142	15.26488411	18.70348809	13.7971931	15.45573008	29.93943451	21.98552602	17.18940754	20.41695225	18.08600096	21.52346426	1.39	0.037
Indoxyl sulfate	nspecified Organic compoun	HMDB0000682	10258	-	C8 H7 N O4	213.00914	212.00186	4.234	64.6	91.3	29.53272342	29.7042473	29.48774386	11.35605296	19.63427339	8.846177694	11.54145206	7.648293174	11.80524986	92.21908721	22.83401041	48.68404822	63.41845863	22.82720931	49.99656276	4.24	0.021
Leupeptin	nspecified Organic compoun	HMDB0254042	3910	-	20 H38 N6	427.30331	426.29603	12.421	NA	64	5.435074055	4.077439567	3.712067138	13.12504916	13.00393685	14.05127618	12.33035299	5.687183843	11.6395598	1.578852636	2.056388893	1.653487223	1.464969926	2.055846018	1.761908939	0.15	0.000
N-Benzylformamide	nspecified Organic compoun	HMDB0304423	-	C15561	C8 H9 N O	119.04944	135.06863	1.306	54.4	65.7	623.4290568	619.881267	632.0513231	532.7851179	518.0967359	596.3894536	694.7940692	655.2151096	599.4561152	882.2325879	689.3460602	763.5949102	813.3632861	776.1567995	784.9387288	1.31	0.004
N-Cyclohexylformamide	nspecified Organic compoun	HMDB0255105	13017	C11519	C7 H13 N O	128.10725	127.09997	13.46	NA	45	39.45344001	22.14556956	28.03451797	22.88826141	26.78608316	21.11778396	12.93349137	18.12819868	20.37076372	17.23296248	16.09317919	10.00037361	9.901992213	12.76375476	13.19845245	0.65	0.033
Phenol sulfate	nspecified Organic compoun	HMDB0060015	74426	C02180	C6 H6 O4 S	173.99783	172.99055	3.878	NA	81.8	6.810869462	5.957689489	6.468484276	2.863913031	5.568166557	0.855315207	3.53333536	0.26708956	2.617563943	24.51695421	4.501032247	4.489848211	13.99343161	12.03849877	11.90795301	4.55	0.041
Phthalic anhydride	nspecified Organic compoun	HMDB0256501	-	-	C8 H4 O3	149.02342	148.01614	11.35	NA	95.3	12058.6418	11913.3509	11509.9022	12072.33203	12505.67662	14516.53971	13536.66684	14260.77663	13378.39837	9972.838789	10815.13474	10905.57469	11054.70101	10820.2867	10713.70719	0.80	0.001
Pipecolic acid	nspecified Organic compoun	HMDB0000070	849	C00408	C6 H11 N O2	129.10236	146.10563	0.834	NA	76.1	93.28182416	93.35387248	93.42579309	79.34724061	82.11035619	95.38843405	97.13392382	88.98432537	88.59285601	108.4844826	88.14897425	123.4293382	106.9047278	111.6494469	107.723394	1.22	0.021
Pipecolic acid ethyl ester	nspecified Organic compoun	-	-	-	C8 H15 N O2	158.11777	157.11049	4.596	NA	34.3	13.75689607	12.98213805	11.5552147	31.1684324	22.13615761	21.20459934	26.2476764	15.19329301	23.19003175	10.13697025	10.45556494	10.1750162	8.730863577	7.904963409	9.480675675	0.41	0.001
Piperidine	nspecified Organic compoun	HMDB0034301	8082	C01746	C5 H11 N	86.09713	85.08986	3.405	NA	80.6	612.7426363	593.0930345	617.5720626	522.6414159	275.4941541	534.8215158	30.16412572	36.10803348	279.845849	509.8355415	490.8618764	605.118305	626.5821592	733.6006748	593.1997114	2.12	0.030
Stercobilin	nspecified Organic compoun	HMDB0240259	-	-	33 H46 N4	595.34931	594.34204	5.535	NA	92	22.70680165	22.81199177	20.89293322	31.59277076	28.33322983	30.33182544	27.06620837	24.52740977	28.37028883	20.71866508	20.60016843	20.14322189	19.00978286	19.49086297	19.99254025	0.70	0.000
Tonalide	nspecified Organic compoun	-	-	-	C18 H26 O	259.20582	258.19854	12.298	NA	49.9	6.408935689	5.513142326	5.10000773	20.59572683	18.34573877	37.57472604	14.41121062	12.47482119	20.68044469	4.302523113	1.667653911	8.153425417	8.402998074	4.209759902	5.347272083	0.26	0.011
Urocanic acid	nspecified Organic compoun	HMDB0000301	736715	C00785	C6 H6 N2 O2	156.07693	138.04309	2.901	NA	39.8	21.3915535	19.72795719	19.77528364	14.52672276	21.25436711	15.42632034	9.375490598	11.05413824	14.32740781	46.66743434	13.12326114	21.83661254	35.3551562	34.12853507	30.22219986	2.11	0.033
Valerophenone	nspecified Organic compoun	HMDB0031208	66093	-	C11 H14 O	145.10133	162.10462	9.547	NA	55.6	288.1361678	272.3944122	279.7966667	328.1689893	297.4579328	340.3221041	302.3758227	328.7568682	319.4163434	277.5016568	284.2337244	174.8690899	275.531256	286.7187385	259.7708931	0.81	0.031

Compound Name	Units	Sex	Control-1	Control-2	Control-3	Control-4	Control-5	Treated-1	Treated-2	Treated-3	Treated-4	Treated-5	FC(Tx/Cntr)	Log10 (Tx/Cntr ratio)	P value
**Cholesterol**	nmol/mg protein	F	300	312	319	305	310	312	311	305	310	311	1.00	0.0008	0.99
		M	305	310	330	312	326	306	316	300	316	326	0.99	-0.0052	0.88
**24HC**	pmol/mg protein	F	1100	1056	1098	1049	1064	1315	1325	1310	1289	1305	1.22	0.0861	<0.0001
		M	1134	1146	1129	1151	1120	1378	1369	1347	1352	1365	1.20	0.0789	<0.0001
**Lathosterol**	pmol/mg protein	F	287	321	312	324	325	375	381	400	389	378	1.23	0.0884	0.042
		M	326	331	342	312	384	396	399	425	384	431	1.20	0.0794	0.017
**Desmosterol**	pmol/mg protein	F	300	312	287	310	295	321	333	318	326	324	1.08	0.0328	0.08
		M	310	345	324	340	329	315	354	367	339	371	1.06	0.0251	0.18
**Acetyl-CoA, mitochondria**	pmol/mg protein	F	82.03	88.12	78.65	75.33	71.25	287.55	289.25	291.01	285.32	280.25	3.63	0.5593	<0.0001
		M	82.09	89.94	78.72	99.82	87.55	361.77	388.04	403.73	322.75	299.60	4.05	0.6078	<0.0001
**Acetyl-CoA, whole brain**	pmol/mg protein	F	135.21	148.15	145.33	151.02	149.11	335.41	342.24	354.25	351.24	359.48	2.39	0.3786	<0.0001
		M	158.18	136.74	150.14	146.65	136.38	357.40	321.36	360.13	352.15	361.24	2.41	0.3814	<0.0001
**Acetylcholine, whole brain**	pmol/µg protein	F	0.61	0.47	0.37	0.41	0.46	0.63	0.65	1.05	0.54	0.69	1.54	0.1872	<0.0001
		M	0.55	0.36	0.54	0.45	0.55	0.98	1.28	1.35	1.31	1.24	2.51	0.4003	<0.0001
**Glucose, plasma**	mg/ml	F	3.41	3.46	4.01	3.45	3.48	3.51	3.60	3.64	4.03	3.84	1.05	0.0192	0.72
		M	3.10	3.09	3.71	3.00	3.21	3.04	3.39	3.23	3.63	3.39	1.04	0.0154	0.87
**Brain glucose uptake**	%	F	69.20	68.10	71.40	64.60	64.20	65.00	70.30	70.60	73.90	72.00	1.04	0.0180	0.64
		M	68.10	67.00	74.30	62.60	65.00	64.60	72.80	75.60	74.30	73.00	1.07	0.0290	0.25
**Glucose, brain**	mg/g brain	F	2.12	2.18	2.37	2.42	2.52	2.52	2.21	2.24	2.2	2.2	0.98	-0.0091	0.95
		M	2.02	2.03	2.12	2.27	2.36	2.32	2.04	1.88	2.05	2.01	0.95	-0.0206	0.73
**Ala**	mg/g brain/30 min (ASR)	F	47.17	38.99	53.01	49.46	40.04	64.92	53.6	45.37	59.04	57.98	1.23	0.0894	0.044
		M	53.42	44.23	60.16	56.19	45.24	76.63	63.62	63.43	69.52	68.43	1.32	0.1199	0.003
**Ala**	mg/g brain	F	89.12	82.42	99.53	102.47	83.39	124.93	114.24	93.22	109.87	120.6	1.23	0.0906	0.019
		M	99.81	92.31	111.47	114.77	93.39	144.92	132.52	128.13	127.45	139.9	1.31	0.1189	0.0006
**Asp**	mg/g brain/30 min (ASR)	F	4.9	4.84	3.44	5.04	5.09	3.5	5	3.64	2.83	4.72	0.84	-0.0733	0.74
		M	10.66	10.85	9.62	11.8	11.5	17.05	16.61	14.82	13.47	17.82	1.47	0.1660	<0.0001
**Asp**	mg/g brain	F	122.48	130.24	142.05	149.71	143.02	151.85	125.34	121.14	124.07	133.92	0.95	-0.0202	0.85
		M	131.62	140.68	153.25	162.09	154.6	172.7	143.98	139.95	142.84	156.04	1.02	0.0077	0.98
**Fumarate**	mg/g brain/30 min (ASR)	F	1.53	2.55	2.34	1.4	2.2	4.06	3.77	3.32	3.54	3.28	1.79	0.2537	0.009
		M	2.52	4.21	4.64	2.3	3.64	8.11	7.54	6.63	7.08	6.56	2.08	0.3170	<0.0001
**Fumarate**	mg/g brain	F	14.73	18.67	19.23	14.87	17.71	26.92	25.02	22.93	26.89	26.05	1.50	0.1761	0.0009
		M	24.31	30.81	31.73	24.53	29.22	53.84	50.03	45.87	53.77	52.09	1.82	0.2596	<0.0001
**Glycerol**	mg/g brain/30 min (ASR)	F	4.87	3.7	4.64	3.4	4.64	2.15	2.64	3.56	2.88	3.6	0.70	-0.1562	0.018
		M	6.69	5.2	6.38	5.83	6.41	4.56	3.85	4.97	4.15	5.13	0.74	-0.1292	0.004
**Glycerol**	mg/g brain	F	62.8	59.8	61.1	60.8	63.9	82.7	81.2	77.4	82	93.2	1.35	0.1305	<0.0001
		M	75.4	71.8	73.3	73	76.7	99.2	97.5	92.9	98.4	111.9	1.35	0.1304	<0.0001
**Glycerol 3-P**	mg/g brain/30 min (ASR)	F	0.131	0.096	0.058	0.065	0.05	0.067	0.119	0.119	0.156	0.019	1.20	0.0792	0.95
		M	0.282	0.293	0.214	0.231	0.177	0.278	0.277	0.284	0.366	0.2149	1.19	0.0742	0.47
**Glycerol 3-P**	mg/g brain	F	4.01	5.88	4.77	5.22	3.86	3.37	4.42	4.39	5.53	3.66	0.90	-0.0457	0.78
		M	4.81	5.72	5.73	6.27	4.64	4.85	5.3	5.27	6.63	4.39	0.97	-0.0118	0.99
**Gly**	mg/g brain/30 min (ASR)	F	0.97	0.56	0.81	0.5	0.53	0.61	0.91	0.79	0.72	0.68	1.10	0.0417	0.94
		M	1.154	0.8	1.01	0.72	0.72	1.03	1.67	1.42	1.51	1.28	1.57	0.1956	0.0045
**Gly**	mg/g brain	F	2.24	2.31	2.32	2.36	2.36	2.43	2.98	2.53	2.87	2.8	1.17	0.0698	0.043
		M	2.36	2.77	2.79	2.54	1.93	3.4	4.17	3.54	4.54	3.91	1.58	0.1983	<0.0001
**Glu/Gln**	mg/g brain/30 min (ASR)	F	5.47	5.83	7.36	6.59	6.22	6.45	8.3	5.89	5.9	5.96	1.03	0.0140	0.97
		M	5.7	6.07	7.67	6.86	6.48	5.86	6.54	5.36	5.36	5.42	0.87	-0.0602	0.34
**Glu/Gln**	mg/g brain	F	202.79	201.12	224.04	236.13	233.3	225.49	209.7	203.4	204.94	206.8	0.96	-0.0190	0.69
		M	211.25	209.5	233.38	245.97	243.03	204.99	190.63	184.91	186.32	188	0.84	-0.0782	0.002
**Lactate**	mg/g brain/30 min (ASR)	F	0.99	0.89	1.48	1.46	1.02	2.8	2.12	1.85	3.27	2.45	2.14	0.3301	0.001
		M	1.35	1.23	2	1.97	1.37	3.68	2.84	2.41	3.26	3.22	1.95	0.2891	0.0003
**Lactate**	mg/g brain	F	2.95	3.36	4.21	4.75	2.9	6.97	5.69	3.84	7.03	6.1	1.63	0.2124	0.0045
		M	3.61	4.11	4.35	4.67	3.56	8.54	6.97	7.2	8.63	7.47	1.91	0.2814	<0.0001
**Malate**	mg/g brain/30 min (ASR)	F	0.73	0.68	0.96	0.44	0.58	0.41	0.81	0.7	0.79	0.67	1.00	-0.0013	>1.0
		M	0.73	0.68	0.96	0.44	0.58	0.41	0.81	0.7	0.79	0.67	1.00	-0.0013	>1.0
**Malate**	mg/g brain	F	4.62	4.58	4.11	2.82	3.38	2.39	4.3	3.98	4.1	4.09	0.97	-0.0147	0.98
		M	4.62	4.58	4.11	3.91	3.38	3.74	4.3	3.98	4.1	4.09	0.98	-0.0083	0.99
**Pro**	mg/g brain/30 min (ASR)	F	0.39	0.24	0.33	0.53	0.36	1.23	0.79	0.48	0.61	0.73	2.08	0.3172	0.04
		M	0.47	0.31	0.41	0.65	0.43	1.51	1.72	1.05	1.39	1.69	3.24	0.5109	<0.0001
**Pro**	mg/g brain	F	4.41	4.29	5.3	6.19	3.99	9.87	10.34	8.28	9.46	10.67	2.01	0.3034	<0.0001
		M	5.07	4.93	6.09	7.11	4.59	17.77	18.61	14.9	17.03	19.21	3.15	0.4982	<0.0001
**Ser**	mg/g brain/30 min (ASR)	F	0.33	0.32	0.4	0.32	0.39	0.31	0.3	0.28	0.31	0.32	0.86	-0.0637	0.09
		M	0.25	0.25	0.31	0.32	0.32	0.33256	0.3	0.28	0.31	0.32	1.06	0.0269	0.76
**Ser**	mg/g brain	F	18.71	19.11	23.68	25.65	21.19	23.43	23.02	21.31	22.63	24.75	1.06	0.0264	0.72
		M	17.77	18.16	22.49	22.1	20.13	23.43	23.02	21.31	22.63	24.75	1.14	0.0584	0.15
**Succinate**	mg/g brain/30 min (ASR)	F	1.89	2.26	1.96	1.58	2.07	3.08	2.87	3.02	2.92	2.65	1.49	0.1731	0.0002
		M	1.83	2.19	1.9	1.53	2	3.17	2.37	2.45	3.01	3.07	1.49	0.1729	0.0003
**Succinate**	mg/g brain	F	11.34	10.43	11.45	11.98	13.49	11.62	11.31	10.89	10.27	11.28	0.94	-0.0253	0.67
		M	11.94	12.97	12.25	10	10.21	10.5478	11.2125	11.22	10.58	11.59	0.96	-0.0171	0.87
**b-Ala**	mg/g brain	F	1.52	1.92	1.97	2.18	2.77	7.15	7.51	7.29	5.04	6.46	3.23	0.5090	<0.0001
		M	2.06	2.59	2.66	2.94	3.73	12.16	12.77	12.4	8.56	10.98	4.07	0.6094	<0.0001
**L-DOPA**	mg/g brain	F	0.06	0.06	0.06	0.06	0.07	0.07	0.06	0.06	0.06	0.06	1.00	0.0000	>1.0
		M	0.07	0.081	0.084	0.08	0.08	0.1	0.101	0.096	0.09	0.09	1.21	0.0819	0.0035
**Ile**	mg/g brain	F	2.01	2.06	2.13	2.36	2.39	2.79	3.25	3.31	3.29	3.29	1.45	0.1628	0.0001
		M	2.61	2.68	2.77	3.07	3.11	4.05	4.11	4.8	4.77	4.77	1.58	0.1987	<0.0001
**Met**	mg/g brain	F	0.16	0.17	0.2	0.22	0.15	0.17	0.17	0.19	0.17	0.17	0.97	-0.0147	0.97
		M	0.16	0.17	0.2	0.22	0.15	0.17	0.17	0.19	0.17	0.17	0.97	-0.0147	0.98
**Pantothenic acid**	mg/g brain	F	0.61	0.63	0.89	0.91	0.72	2.03	2.53	1.81	1.31	1.73	2.50	0.3984	0.015
		M	0.95	0.98	1.38	1.41	1.12	4.07	5.06	3.62	2.63	3.45	3.22	0.5084	<0.0001
**Phe**	mg/g brain	F	3.25	3.17	3.7	3.78	3.56	2.79	2.56	2.5	2.7	2.63	0.75	-0.1221	<0.0001
		M	3.9	3.8	4.44	4.54	4.27	2.33	2.13	2.09	2.25	2.19	0.52	-0.2802	<0.0001
**Tyramine**	mg/g brain	F	0.17	0.17	0.19	0.2	0.19	0.18	0.17	0.16	0.16	0.17	0.91	-0.0395	0.21

[illegible]	[illegible]	M	0.18	0.17	0.2	0.2	0.18	0.2	0.18	0.17	0.17	0.17	0.96	-0.0191	0.73
**Tyr**	mg/g brain	F	3.26	3.25	3.6	3.69	3.72	5.22	4.91	4.63	4.55	4.56	1.36	0.1343	<0.0001
		M	4.39	4.38	4.87	4.98	5.02	7.83	7.36	6.95	6.82	6.85	1.51	0.1804	<0.0001
**Thr**	mg/g brain	F	54.07	55.61	58.5	48.12	51.72	46.5	44.81	40.7	46.49	42.65	0.83	-0.0835	0.092
		M	64.89	66.74	70.2	47.8	50.53	69.75	67.21	61.05	69.74	63.98	1.11	0.0434	0.352
**Val**	mg/g brain	F	1.59	1.73	1.73	2.57	1.68	5.91	6.25	4.89	7.06	7.06	3.35	0.5253	<0.0001
		M	2.3	2.51	2.51	3.72	2.43	8.58	9.07	7.09	10.23	10.24	3.36	0.5259	<0.0001
**Myristic acid (14:0), free**	mg/g brain	F	0.075129	0.086191	0.070362	0.072791	0.085046	0.187381	0.184781	0.15293	0.121789	0.121867	1.97	0.2953	0.033
		M	0.090155	0.10343	0.084435	0.08735	0.102055	0.224858	0.221737	0.183516	0.146147	0.14624	1.97	0.2953	0.019
**Myristic acid (14:0), esterified**	mg/g brain	F	0.28006	0.310648	0.270466	0.290069	0.298974	0.327775	0.349848	0.378432	0.338571	0.24818	1.13	0.0542	0.94
		M	0.336072	0.372778	0.324559	0.348083	0.358768	0.39333	0.419818	0.454118	0.406285	0.297816	1.13	0.0542	0.39
**Myristic acid (14:0), total**	mg/g brain	F	0.355189	0.39684	0.340828	0.36286	0.38402	0.515157	0.534629	0.531362	0.46036	0.370047	1.31	0.1175	0.0034
		M	0.426226	0.476207	0.408994	0.435432	0.460824	0.618188	0.641555	0.637634	0.552432	0.444056	1.31	0.1175	0.0002
**Palmitic acid (16:0), free**	mg/g brain	F	0.735801	0.708321	0.724687	0.647298	0.746561	0.825455	0.795912	0.792952	0.743091	0.906672	1.14	0.0572	>1.0
		M	1.471602	1.416643	1.449374	1.294596	1.493123	2.313636	2.48978	2.573788	2.577278	2.266679	1.72	0.2343	<0.0001
**Palmitic acid (16:0), esterified**	mg/g brain	F	3.438433	3.400274	3.3411	2.84779	3.287766	3.551564	3.214926	3.257	3.240615	3.720603	1.04	0.0175	>1.0
		M	6.042019	5.978815	5.869037	4.996556	5.768662	5.744993	5.089726	5.141937	5.111463	6.06241	0.95	-0.0234	0.86
**Palmitic acid (16:0), total**	mg/g brain	F	4.174234	4.108595	4.065787	3.495088	4.034328	4.477019	4.210838	4.286515	4.271526	4.627274	1.10	0.0415	0.52
		M	7.513621	7.395458	7.318411	6.291152	7.261785	8.058629	8.579506	8.715726	8.688741	8.729089	1.20	0.0775	0.048
**Stearic (18:0), free**	mg/g brain	F	1.13291	1.12654	1.14323	0.94502	1.19478	1.34064	1.29536	1.34277	1.3778	1.3115	1.20	0.0803	0.87
		M	1.35949	1.35185	1.32387	1.13403	1.43373	1.54064	1.49536	1.34277	1.5778	1.6115	1.15	0.0592	0.99
**Stearic (18:0), esterified**	mg/g brain	F	3.97575	4.35808	4.6021	4.86031	4.90739	5.76097	5.74898	6.58484	6.06516	6.03778	1.33	0.1239	<0.0001
		M	7.06979	7.69777	8.08991	8.44477	8.63484	11.6022	11.5445	12.9269	12.1795	12.1572	1.51	0.1797	<0.0001
**Stearic (18:0), total**	mg/g brain	F	5.10866	5.48462	5.70532	5.80533	6.10217	7.3016	7.24434	7.92761	7.64296	7.64929	1.34	0.1268	<0.0001
		M	8.42928	9.04962	9.41378	9.57879	10.0686	13.1429	13.0398	14.2697	13.7573	13.7687	1.46	0.1645	<0.0001
**Arachidic acid (20:0), free**	mg/g brain	F	0.0302	0.04365	0.0373	0.03929	0.03964	0.04398	0.04792	0.04118	0.04419	0.04228	1.16	0.0626	0.99
		M	0.04832	0.06985	0.05968	0.06286	0.06343	0.07037	0.07667	0.06589	0.0707	0.06764	1.16	0.0626	0.97
**Arachidic acid (20:0), esterified**	mg/g brain	F	0.13647	0.1463	0.12269	0.13564	0.1479	0.13369	0.13342	0.14692	0.15321	0.13744	1.02	0.0098	>1.0
		M	0.21835	0.23408	0.1963	0.21702	0.23664	0.2139	0.21347	0.23506	0.24514	0.2199	1.02	0.0098	>1.0
**Arachidic acid (20:0), total**	mg/g brain	F	0.16666	0.18995	0.15999	0.17493	0.18754	0.17767	0.18134	0.1881	0.1974	0.17972	1.05	0.0218	0.98
		M	0.26666	0.30393	0.25598	0.27988	0.30007	0.28427	0.29014	0.30096	0.31584	0.28755	1.05	0.0218	0.69
**11-Hexadecenoic acid (16:1), free**	mg/g brain	F	0.053212	0.051285	0.041507	0.044439	0.048041	0.048616	0.059003	0.05003	0.045098	0.044813	1.04	0.0162	>1.0
		M	0.053212	0.051285	0.041507	0.044439	0.048041	0.048616	0.059003	0.05003	0.045098	0.044813	1.04	0.0162	>1.0
**11-Hexadecenoic acid (16:1), esterified**	mg/g brain	F	0.154975	0.131788	0.132583	0.11146	0.121927	0.143161	0.125828	0.164415	0.136549	0.119307	1.06	0.0236	>1.0
		M	0.154975	0.131788	0.132583	0.11146	0.121927	0.143161	0.125828	0.164415	0.136549	0.119307	1.06	0.0236	0.99
**11-Hexadecenoic acid (16:1), total**	mg/g brain	F	0.208187	0.183073	0.17409	0.1559	0.169967	0.191778	0.184832	0.214445	0.181647	0.16412	1.05	0.0217	0.99
		M	0.208187	0.183073	0.17409	0.1559	0.169967	0.191778	0.184832	0.214445	0.181647	0.16412	1.05	0.0217	0.99
**Oleic/Elaidic acid (18:1), free**	mg/g brain	F	0.059154	0.063805	0.062914	0.059506	0.069746	0.066586	0.064764	0.046819	0.044557	0.048995	0.86	-0.0644	0.99
		M	0.068027	0.073376	0.072351	0.068432	0.080207	0.069879	0.082147	0.070228	0.066835	0.073493	1.00	0.0002	>1.0
**Oleic/Elaidic acid (18:1), esterified**	mg/g brain	F	0.141949	0.137923	0.105579	0.108836	0.128384	0.136086	0.156955	0.166234	0.143023	0.127594	1.17	0.0690	0.42
		M	0.133076	0.128352	0.096142	0.09991	0.117922	0.112793	0.129573	0.142824	0.120745	0.103096	1.06	0.0247	0.99
**Oleic/Elaidic acid (18:1), total**	mg/g brain	F	0.201102	0.201729	0.168494	0.168342	0.198129	0.182672	0.211719	0.213052	0.18758	0.176589	1.04	0.0154	0.99
		M	0.201102	0.201729	0.168494	0.168342	0.198129	0.182672	0.211719	0.213052	0.18758	0.176589	1.04	0.0154	0.99
**Linoleic acid (18:2), free**	mg/g brain	F	0.025384	0.023184	0.020483	0.023721	0.026598	0.023663	0.026906	0.025986	0.022557	0.020876	1.01	0.0022	>1.0
		M	0.029954	0.027358	0.02417	0.027991	0.031385	0.027923	0.031749	0.030664	0.026617	0.024634	1.01	0.0022	>1.0
**Linoleic acid (18:2), esterified**	mg/g brain	F	0.098429	0.081763	0.082482	0.070795	0.113066	0.092095	0.077307	0.089928	0.077326	0.069363	0.91	-0.0413	0.99
		M	0.116146	0.09648	0.097329	0.083538	0.133417	0.108672	0.091222	0.106116	0.091244	0.081848	0.91	-0.0413	0.98
**Linoleic acid (18:2), total**	mg/g brain	F	0.123813	0.104947	0.102965	0.094516	0.139663	0.115758	0.104213	0.115915	0.099883	0.090239	0.93	-0.0318	0.99
		M	0.1461	0.123838	0.121499	0.111529	0.164803	0.136594	0.122971	0.136779	0.117862	0.106482	0.93	-0.0318	0.98
**Gondoic acid (20:1), free**	mg/g brain	F	0.183758	0.182928	0.192499	0.187057	0.200948	0.183011	0.123818	0.276607	0.277904	0.254456	1.18	0.0711	0.99
		M	0.211322	0.210367	0.221374	0.215115	0.23109	0.290963	0.27239	0.218098	0.24959	0.222625	1.15	0.0610	0.99
**Gondoic acid (20:1), esterified**	mg/g brain	F	0.745646	0.88925	0.680566	0.789861	0.721123	0.955852	0.919649	0.879615	0.960008	0.858117	1.20	0.0774	0.15
		M	0.857493	1.022637	0.782651	0.90834	0.829291	1.29923	1.142597	1.211557	1.104009	0.986835	1.31	0.1157	0.012
**Gondoic acid (20:1), total**	mg/g brain	F	0.929404	0.917218	0.873065	0.976918	0.92207	1.138863	1.043467	1.156222	1.237912	1.112573	1.23	0.0905	0.0005
		M	1.068815	1.233005	1.004025	1.123455	1.060381	1.590193	1.414987	1.429655	1.353599	1.209459	1.27	0.1054	<0.0001
**Arachidonic (20:4), free**	mg/g brain	F	0.89595	0.973894	0.937056	0.843081	0.941385	0.944184	1.101449	1.038916	0.920438	0.915794	1.07	0.0301	>1.0
		M	0.89595	0.973894	0.937056	0.843081	0.941385	0.944184	1.101449	1.038916	0.920438	0.915794	1.07	0.0301	>1.0
**Arachidonic (20:4), esterified**	mg/g brain	F	4.582851	4.943535	5.191019	4.638556	4.477092	4.576107	4.25102	4.168363	5.210701	4.255976	0.94	-0.0257	0.88
		M	3.017479	3.252841	3.44014	3.072374	2.928956	3.302194	3.015835	2.966684	3.795823	3.062491	1.03	0.0118	>1.0
**Arachidonic (20:4), total**	mg/g brain	F	5.478801	5.917429	6.128075	5.481637	5.418477	5.520291	5.352469	5.20728	6.13114	5.171771	0.96	-0.0162	0.98
		M	3.913429	4.226735	4.377197	3.915455	3.87034	4.246377	4.117284	4.0056	4.716261	3.978285	1.04	0.0160	0.99
**Docosahexaenoic acid (22:6), free**	mg/g brain	F	0.455501	0.48949	0.451214	0.420545	0.448724	0.521666	0.515276	0.50886	0.594975	0.558618	1.19	0.0761	0.95
		M	0.379584	0.407909	0.376011	0.350454	0.373936	0.394444	0.410184	0.37924	0.39665	0.425745	1.06	0.0264	>1.0
**Docosahexaenoic acid (22:6), esterified**	mg/g brain	F	2.020406	1.873455	1.99644	1.875144	1.877061	2.157404	2.007455	2.292481	2.047582	1.733466	1.06	0.0260	0.73
		M	1.52496	1.409741	1.506799	1.415461	1.415129	1.720225	1.607302	1.821791	1.636086	1.398934	1.13	0.0513	0.15
**Docosahexaenoic acid (22:6), total**	mg/g brain	F	2.275907	2.262945	2.147654	2.295689	2.325785	2.79907	2.727313	2.801341	2.742557	2.592084	1.21	0.0821	0.025
		M	1.904544	1.81765	1.882811	1.765915	1.789065	2.114669	2.017486	2.201032	2.032736	1.82468	1.11	0.0463	0.062
**Nervoric acid (24:1), free**	mg/g brain	F	0.029407	0.030123	0.030728	0.033758	0.035779	0.047091	0.054551	0.051633	0.050605	0.053771	1.61	0.2075	0.047
		M	0.052344	0.053619	0.054695	0.060089	0.063687	0.083821	0.097101	0.091906	0.090077	0.095712	1.61	0.2075	<0.0001
**Nervoric acid (24:1), esterified**	mg/g brain	F	0.173746	0.176375	0.162236	0.182948	0.176349	0.15784	0.160696	0.16495	0.1538	0.160791	0.92	-0.0383	0.31
		M	0.309267	0.313948	0.28878	0.325648	0.313901	0.376533	0.384918	0.362918	0.369175	0.384868	1.21	0.0830	<0.0001
**Nervoric acid (24:1), total**	mg/g brain	F	0.203152	0.206498	0.192964	0.216706	0.212128	0.20493	0.215247	0.216583	0.204406	0.214562	1.02	0.0101	0.99
		M	0.361611	0.367567	0.343475	0.385737	0.377589	0.460354	0.482019	0.454824	0.459252	0.48058	1.27	0.1048	<0.0001

Supplementary Table 4. Notable protein enrichments in EFV-treated *vs* control 5XFAD mice. ALS, amyotrophic lateral sclerosis; **Ctr**, control mice; **ETC,** mitochondrial electron transport chain; **F**, female mice; **GB**, glioblastoma; **GL**, glioma; **FTD**, frontotemporal dementia; **M**, male mice; **MS**, multiple sclerosis; **PD**, Parkinson's disease, **Tx**, EFV-treated mice.

Protein/sex	Tx/Ctr, fold change (x)		Protein function	Disease
	DEP	DAP (site), x		
**The Brain Diseases Group**				
**Miscellaneous proteins**				
BACE1/F	Ctr only		Initiates the formation of the Aβ peptides; expression and activity are elevated in AD[1-3].	AD
MBP/F		(72), 0.5x	The second most abundant protein in myelin sheath; MBP acetylation leads to myelin damage[4-6].	MS, AD
PIK3CA/F	Ctr only		Phosphorylates phosphatidylinositol 4,5-bisphosphate to yield phosphatidylinositol 3,4,5-triphosphate; one of the activating drivers of cancers, including glioblastoma (GB)[7-9].	GB
RAB39B/F		(148), 0.2x	Small GTPase involved in autophagy; mutations in *RAB39B* cause early onset PD and may contribute to ALS and FTD[10-12].	PD, ALS, FTD
SNCA/F		(96), 3.0x	Regulates synaptic vesicle trafficking and neurotransmitter release; acetylation decreases SNCA propensity to aggregate[13].	PD
NOL3/M	0.8x		Endogenous apoptosis repressor that blocks multiple modes of cell death; markedly induced in a variety of cancers including glioma (GL)[14-16].	GL
NRAS/M	0.8x		Small GTPase and a proto-oncogene; protein levels of NRAS are increased in glioma specimens; mutations in *NRAS* lead to various cancers including GL[17].	GL
RAB18/M	1.3x		Small GTPase; regulates membrane traffic between the endoplasmic reticulum and lipid droplets; increased RAB18 expression is associated with a poor prognosis of GL due to increased cell proliferation and decreased apoptosis[18-20]	GL
**Cytoskeleton-related proteins**				
ACTB/M		(326), 1.9x	Isoforms 1 and 2, respectively, of actin, which forms microfilaments; actin acetylation could be a cytoskeletal regulatory mechanism with acetylation of ACTG1 at Lys61 stabilizing stress fiber[21-23].	AD, PD, HD
ACTG1/M		(81), 1.8x		
SEPTIN5/F	0.5x		Filament forming cytoskeletal GTPase[24]; could increase the Aβ levels; accumulated in dopaminergic neurons due to parkin dysfunction leading to neurotoxicity[25, 26].	AD, PD
SPTBN1/F		(1809), 0.8x	Cytoskeletal protein spectrin; contributes to mechanical support of plasma membranes; mutations in *SPTBN1* cause autism spectrum disorder, attention deficit hyperactivity disorder and other neurologic conditions[27].	ASD
SPTBN2/M		(1478), 1.6x	Cytoskeletal protein spectrin; contributes to mechanical support of plasma membranes; mutations in *SPTBN2* cause SCA5[27].	SCA5
TUBA4A/M		(40), 2.6x	Cytoskeletal protein tubulin; polymerizes into microtubules; acetylation of tubulins increases microtubule stability, which is impaired in AD, PD, and ALS[28].	AD, PD, ALS
TUBB4B/F	0.8x		Cytoskeletal protein tubulin; polymerizes into microtubules; TUBB4B expression is decreased in AD and increased in PD[29-31].	AD, PD
TUBB6/M	Ctr only		Cytoskeletal protein tubulin; polymerizes into microtubules; TUBB6 expression is decreased in AD and high expression is linked to a poor prognosis in various cancers including glioblastoma[32-34]	AD, GB
TPPP3/F		(37, 77), 0.8x; (218), 3.9x	Promotes tubulin polymerization; protein levels are increased with GB grade[35].	GB
**The mitochondrial electron transport chain (ETC) proteins**				
NDUFA7/F		(48), 0.8x	Accessory subunit of NADH dehydrogenase 1 in Complex 1[36].	AD, PD. HD
NDUFB7/F		(97), 0.7x	Accessory subunit of NADH dehydrogenase 1 in Complex 1; reduced abundance is fatal due to multiple manifestations including encephalopathy[36,37]	
NDUFA13/M	0.8x		Accessory subunit of NADH dehydrogenase 1 in Complex 1; mutations in NDUFA13 destabilize Complex 1 due to impaired interaction with NDUFA7 and slowly progressive neurological symptoms[36,38,39].	
SDHA/M		(633), 1.4x	Flavoprotein subunit of succinate dehydrogenase in Complex 2; acetylation of SDHA generally inhibits its activity[40].	
UQCRC1/M		(138), 1.3x	Component of the ubiquinol-cytochrome c oxidoreductase in Complex 3; downregulated in the AD & cause PD when mutated[41,42].	
UQCR11/F	0.8x		Component of the ubiquinol-cytochrome c oxidoreductase in Complex 3; weakens mitochondrial respiration[43] when suppressed.	
COA5/F	Tx only		Essential for the Complex 4 assembly[44].	
ATP5H/M		(32), 0.7x; (63), 1.2x	Subunit d of ATP synthase in Complex 5[45].	
ATP5MG/F		(35), 0.7x	Subunit g of ATP synthase in Complex 5[45].	

ATP5PB/**M**		(159), 1.7x; (194), 1.8x;	Subunit b of ATP synthase in Complex 5[45].
ATP5PF/**F**		(41), 0.5x	Subunit F6 of ATP synthase in Complex 5[45]
**The Neurotransmission Group**			
**Glutamatergic neurotransmission**			
DLGAP1/**M**	0.8x		Act as scaffold proteins in the brain postsynaptic density of glutamatergic neurons and contribute to synaptic scaling, i.e., reset of neuronal firing to "normal' levels[46].
DLGAP4/**F**		(153), 0.2x	
GLS/**F**		(169), 3.3x	Generates Glu from Gln and controls the brain Glu levels; acetylations at sites other than Lys169 inhibit enzyme activity[47,48].
GRM2,4/**M**	1.5x		Metabotropic glutamate receptors 2 and/or 4, respectively; activated by Glu and suppress excessive neurotransmission by inhibiting Glu or γGABA release[49].
NETO1/**F**	Ctr only		An auxiliary protein required for normal abundance of NMDARs[50].
**Glycinergic neurotransmission**			
GLRB/**F**	Ctr only		Anchors Gly receptors at synaptic sites and determines their ligand binding properties; mutations in *GLRB* disrupt glycinergic neurotransmission[51-53].
SLC6A9/**F**	0.5x		Removes Gly from the synaptic cleft; SLC6A9 lack leads to a Gly accumulation and enhances glycinergic neuro-transmission[54].
GLDC/**M**		(519), 3.5x	Initiates Gly breakdown; mutations in *GLDC* cause Gly accumulation in all body tissues; Lys519 acetylation impairs enzyme activity[55,56].
ABHD4/**F**	Ctr only		Contributes to the biosynthesis of anandamides by hydrolyzing acyl chains from N-acyl phosphatidylethanolamine[57].
**Cannabinoid-mediated**			
ABHD4/**F**	Ctr only		Contributes to the biosynthesis anandamides by hydrolyzing acyl chains from N-acyl phosphatidylethanolamine[57].
**GABAergic neurotransmission**			
CBLN4/**F**	0.7x		Acts as a synaptic organizer but is not a major contributor to the formation or maintenance of GABAergic and other synapses; yet, *Cbln4*$^{-/-}$ mice exhibit major behavioral changes[58].
GABRA1/**F**		(417), 1.2x	$GABA_A$ receptor subunit α-1; contributes to synaptic contact formation[59].
SLC4A10/**F**		(960), Ctr only	Localizes to inhibitory presynapses and is involved in regulation of intracellular pH; promotes GABA release, reduces the excitability of CA1 pyramidal neurons, and modulates short-term synaptic plasticity[60].
SLC6A1/**M**		(26), 1.2x	Found primarily in the presynaptic membrane and is responsible for the reuptake of GABA from the synapse[61].
**The Vascular System Group**			
FSTL1/**F**	Ctr only		Secreted muscle protein involved in various physiological processes such as angiogenesis, afferent synaptic transmission, immune response, cell proliferation and differentiation[62-65].
MYLK/**F**	1.3x		Phosphorylates the regulatory myosin light chain and induces actomyosin contraction; contributes to capillary/venule permeability; and regulates arteriolar vasoconstriction[66,67].
PIK3CA/**F**	Ctr only		Converts phosphatidylinositol 4,5-bisphosphate into phosphatidylinositol 3,4,5-trisphosphate; a key player in a signaling pathway responsible for regulation of cell survival, proliferation, apoptosis, angiogenesis, and metabolism. Activating mutations lead to aberrant vascular growth and vascular malformations[68-70].
PRMT7/**F**	2.5x		Transfers methyl groups from S-adenosylmethionine to specific arginine residues on protein substrates; was shown to prevent neurovascular uncoupling, blood-brain barrier permeability, and mitochondrial dysfunction in repetitive and mild traumatic brain injury[71].
ZNF148/**F**	Ctr only		A transcription factor acting as both an activator and repressor of gene expression; linked to various cancers and developmental disorders; ZNF148 deletion attenuates abdominal aortic aneurysm formation and malignant phenotype suggesting involvement in vascular stability and tumor microenvironments[72,73].
EMC10/**M**	Ctr only		Part of the protein complex that enables insertion of various proteins into the endoplasmic reticulum membranes, thus indirectly regulating many cellular processes; angiogenic growth factor promoting tissue repair after myocardial infarction[74].
VEGFA/**M**		(146), 1.2x	A key protein, which promotes vasculogenesis and angiogenesis and increases vascular permeability; acts by binding to receptors and is involved in both normal physiological processes and pathological conditions [75,76]. Lys acetylation can impair VEGF signaling as Lys residues participate in the interaction with the VEGF receptors[77,78].

Supplementary References

1. Hussain I, Powell D, Howlett DR*, et al*. Identification of a novel aspartic protease (Asp 2) as beta-secretase. *Mol Cell Neurosci*. 1999;14:419-427.
2. Ohno M. BACE1 as an early biomarker and its relevance to risk factors for Alzheimer's disease. *Brain Research Bulletin*. 2025;230:111475.
3. Zacchetti D, Chieregatti E, Bettegazzi B*, et al*. BACE1 expression and activity: relevance in Alzheimer's disease. *Neurodegener Dis*. 2007;4:117-126.
4. Zhang C, Walker AK, Zand R, Moscarello MA, Yan JM, Andrews PC. Myelin basic protein undergoes a broader range of modifications in mammals than in lower vertebrates. *J Proteome Res*. 2012;11:4791-4802.
5. Lillico R, Zhou T, Khorshid Ahmad T*, et al*. Increased Post-Translational Lysine Acetylation of Myelin Basic Protein Is Associated with Peak Neurological Disability in a Mouse Experimental Autoimmune Encephalomyelitis Model of Multiple Sclerosis. *J Proteome Res*. 2018;17:55-62.
6. Jahn O, Siems SB, Kusch K*, et al*. The CNS Myelin Proteome: Deep Profile and Persistence After Post-mortem Delay. *Front Cell Neurosci*. 2020;14:239.
7. Meier TI, Cook JA, Thomas JE*, et al*. Cloning, expression, purification, and characterization of the human Class Ia phosphoinositide 3-kinase isoforms. *Protein Expr Purif*. 2004;35:218-224.
8. Parsons DW, Jones S, Zhang X*, et al*. An Integrated Genomic Analysis of Human Glioblastoma Multiforme. *Science*. 2008;321:1807-1812.
9. Hoxhaj G, Manning BD. The PI3K–AKT network at the interface of oncogenic signalling and cancer metabolism. *Nature Reviews Cancer*. 2020;20:74-88.
10. Sellier C, Campanari ML, Julie Corbier C*, et al*. Loss of C9ORF72 impairs autophagy and synergizes with polyQ Ataxin-2 to induce motor neuron dysfunction and cell death. *Embo j*. 2016;35:1276-1297.
11. Corbier C, Sellier C. C9ORF72 is a GDP/GTP exchange factor for Rab8 and Rab39 and regulates autophagy. *Small GTPases*. 2017;8:181-186.
12. Wilson GR, Sim JCH, McLean C*, et al*. Mutations in RAB39B cause X-linked intellectual disability and early-onset parkinson disease with α-synuclein pathology. *American Journal of Human Genetics*. 2014;95:729-735.
13. Hassanzadeh K, Liu J, Maddila S, Mouradian MM. Posttranslational Modifications of α-Synuclein, Their Therapeutic Potential, and Crosstalk in Health and Neurodegenerative Diseases. *Pharmacol Rev*. 2024;76:1254-1290.
14. Koseki T, Inohara N, Chen S, Nunez G. ARC, an inhibitor of apoptosis expressed in skeletal muscle and heart that interacts selectively with caspases. *Proc Natl Acad Sci U S A*. 1998;95:5156-5160.
15. Mercier I, Vuolo M, Jasmin JF*, et al*. ARC (apoptosis repressor with caspase recruitment domain) is a novel marker of human colon cancer. *Cell Cycle*. 2008;7:1640-1647.
16. Wang Q, Li A, Wang H, Wang J. Knockdown of apoptosis repressor with caspase recruitment domain (ARC) increases the sensitivity of human glioma cell line U251MG to VM-26. *Int J Clin Exp Pathol*. 2012;5:555-561.
17. Zhi T, Jiang K, Zhang C*, et al*. MicroRNA-1301 inhibits proliferation of human glioma cells by directly targeting N-Ras. *Am J Cancer Res*. 2017;7:982-998.
18. Martin S, Driessen K, Nixon SJ, Zerial M, Parton RG. Regulated Localization of Rab18 to Lipid Droplets: EFFECTS OF LIPOLYTIC STIMULATION AND INHIBITION OF LIPID DROPLET CATABOLISM*. *Journal of Biological Chemistry*. 2005;280:42325-42335.
19. Liu Q, Tang H, Liu X*, et al*. miR-200b as a prognostic factor targets multiple members of RAB family in glioma. *Med Oncol*. 2014;31:859.
20. Yang K, Wang Z. Rab18 interacted with V-set and immunoglobulin domain-containing 4 (VSIG4) to involve in the apoptosis of glioma and the sensitivity to temozolomide. *Bioengineered*. 2021;12:1391-1402.
21. A M, Latario CJ, Pickrell LE, Higgs HN. Lysine acetylation of cytoskeletal proteins: Emergence of an actin code. *Journal of Cell Biology*. 2020;219

22. Bernstein BW, Maloney MT, Bamburg JR. Actin and Diseases of the Nervous System. *Adv Neurobiol*. 2011;5:201-234.
23. Mann N, Surabhi K, Sharp J*, et al*. Identification of actin mutants with neurodegenerative disease-like phenotypes via mutagenesis of the actin-ATP interface. *Frontiers in Cellular Neuroscience*. 2025;Volume 19 - 2025
24. Alkhanjari RR, Alhajeri MM, Bhamidimarri PM*, et al*. Septins in the nervous system: from cytoskeletal dynamics to neurological disorders. *Cell Commun Signal*. 2025;23:425.
25. Marttinen M, Ferreira CB, Paldanius KMA*, et al*. Presynaptic Vesicle Protein SEPTIN5 Regulates the Degradation of APP C-Terminal Fragments and the Levels of Aβ. *Cells*. 2020;9
26. Son JH, Kawamata H, Yoo MS*, et al*. Neurotoxicity and behavioral deficits associated with Septin 5 accumulation in dopaminergic neurons. *J Neurochem*. 2005;94:1040-1053.
27. Lorenzo DN, Edwards RJ, Slavutsky AL. Spectrins: molecular organizers and targets of neurological disorders. *Nat Rev Neurosci*. 2023;24:195-212.
28. Li L, Yang XJ. Tubulin acetylation: responsible enzymes, biological functions and human diseases. *Cell Mol Life Sci*. 2015;72:4237-4255.
29. Sanzhaeva U, Boyd-Pratt H, Bender PTR*, et al*. TUBB4B is essential for the cytoskeletal architecture of cochlear supporting cells and motile cilia development. *Communications Biology*. 2024;7:1146.
30. Shu Q, Liu R, Pang X, Huang X, Pang C. The role of microtubule proteins TUBB2A, TUBB3, and TUBB4B in neuronal dysfunction in Alzheimer's disease: a bioinformatics analysis. *Aging Advances*. 2025;2
31. López-Cerdán A, Andreu Z, Hidalgo MR*, et al*. Unveiling sex-based differences in Parkinson's disease: a comprehensive meta-analysis of transcriptomic studies. *Biol Sex Differ*. 2022;13:68.
32. Li Y, Shao Z, Jiang J, Wang H, Zhang M. Transcription factor NFKB1 mediates TUBB6 to promote the proliferation and suppress apoptosis in glioma via Wnt/β-catenin signaling pathway. *Discov Oncol*. 2025;16:444.
33. Jiang L, Zhu X, Yang H, Chen T, Lv K. Bioinformatics Analysis Discovers Microtubular Tubulin Beta 6 Class V (TUBB6) as a Potential Therapeutic Target in Glioblastoma. *Front Genet*. 2020;11:566579.
34. Liang WS, Dunckley T, Beach TG*, et al*. Altered neuronal gene expression in brain regions differentially affected by Alzheimer's disease: a reference data set. *Physiol Genomics*. 2008;33:240-256.
35. Xu X, Hou Y, Long N*, et al*. TPPP3 promote epithelial-mesenchymal transition via Snail1 in glioblastoma. *Sci Rep*. 2023;13:17960.
36. Stroud DA, Surgenor EE, Formosa LE*, et al*. Accessory subunits are integral for assembly and function of human mitochondrial complex I. *Nature*. 2016;538:123-126.
37. Correia SP, Moedas MF, Naess K*, et al*. Severe congenital lactic acidosis and hypertrophic cardiomyopathy caused by an intronic variant in NDUFB7. *Hum Mutat*. 2021;42:378-384.
38. Angebault C, Charif M, Guegen N*, et al*. Mutation in NDUFA13/GRIM19 leads to early onset hypotonia, dyskinesia and sensorial deficiencies, and mitochondrial complex I instability. *Hum Mol Genet*. 2015;24:3948-3955.
39. Máximo V, Botelho T, Capela J*, et al*. Somatic and germline mutation in GRIM-19, a dual function gene involved in mitochondrial metabolism and cell death, is linked to mitochondrion-rich (Hürthle cell) tumours of the thyroid. *British Journal of Cancer*. 2005;92:1892-1898.
40. Finley LW, Haas W, Desquiret-Dumas V*, et al*. Succinate dehydrogenase is a direct target of sirtuin 3 deacetylase activity. *PLoS One*. 2011;6:e23295.
41. Lin CH, Tsai PI, Lin HY*, et al*. Mitochondrial UQCRC1 mutations cause autosomal dominant parkinsonism with polyneuropathy. *Brain*. 2020;143:3352-3373.
42. Zhang J, Wu Z, Long Z, Feng C, Bai F, Li H. UQCRC1 is a Key Pathogenic Determinant and Potential Therapeutic Target for Cognitive Impairment in Alzheimer's Disease. *Mol Neurobiol*. 2025;62:13786-13806.
43. Ma W, Tian Y, Shi L*, et al*. N-Acetyltransferase 10 represses Uqcr11 and Uqcrb independently of ac4C modification to promote heart regeneration. *Nat Commun*. 2024;15:2137.

44. Tang JX, Cabrera-Orefice A, Meisterknecht J, *et al*. COA5 has an essential role in the early stage of mitochondrial complex IV assembly. *Life Sci Alliance*. 2025;8
45. Lai Y, Zhang Y, Zhou S, *et al*. Structure of the human ATP synthase. *Mol Cell*. 2023;83:2137-2147.e2134.
46. Rasmussen AH, Rasmussen HB, Silahtaroglu A. The DLGAP family: neuronal expression, function and role in brain disorders. *Mol Brain*. 2017;10:43.
47. Xu G, Qu J, Zhang M. HDAC4-mediated Deacetylation of Glutaminase Facilitates Glioma Stemness. *Curr Cancer Drug Targets*. 2023;23:742-750.
48. El Hage M, Masson J, Conjard-Duplany A, Ferrier B, Baverel G, Martin G. Brain slices from glutaminase-deficient mice metabolize less glutamine: a cellular metabolomic study with carbon 13 NMR. *J Cereb Blood Flow Metab*. 2012;32:816-824.
49. Ribeiro FM, Vieira LB, Pires RGW, Olmo RP, Ferguson SSG. Metabotropic glutamate receptors and neurodegenerative diseases. *Pharm Res*. 2017;115:179-191.
50. Ng D, Pitcher GM, Szilard RK, *et al*. Neto1 is a novel CUB-domain NMDA receptor-interacting protein required for synaptic plasticity and learning. *PLoS Biol*. 2009;7:e41.
51. Xu T-L, Gong N. Glycine and glycine receptor signaling in hippocampal neurons: Diversity, function and regulation. *Prog Neurobiol*. 2010;91:349-361.
52. Rees MI, Lewis TM, Kwok JB, *et al*. Hyperekplexia associated with compound heterozygote mutations in the beta-subunit of the human inhibitory glycine receptor (GLRB). *Hum Mol Genet*. 2002;11:853-860.
53. Chung SK, Bode A, Cushion TD, *et al*. GLRB is the third major gene of effect in hyperekplexia. *Hum Mol Genet*. 2013;22:927-940.
54. Eulenburg V, Hülsmann S. Synergistic Control of Transmitter Turnover at Glycinergic Synapses by GlyT1, GlyT2, and ASC-1. *International Journal of Molecular Sciences*. 2022;23(5):2561. doi:10.3390/ijms23052561 https://pmc.ncbi.nlm.nih.gov/articles/PMC8909939/
55. Dinopoulos A, Kure S, Chuck G, *et al*. Glycine decarboxylase mutations: a distinctive phenotype of nonketotic hyperglycinemia in adults. *Neurology*. 2005;64:1255-1257.
56. Liu R, Zeng LW, Gong R, Yuan F, Shu HB, Li S. mTORC1 activity regulates post-translational modifications of glycine decarboxylase to modulate glycine metabolism and tumorigenesis. *Nat Commun*. 2021;12:4227.
57. Lee HC, Simon GM, Cravatt BF. ABHD4 regulates multiple classes of N-acyl phospholipids in the mammalian central nervous system. *Biochemistry*. 2015;54:2539-2549.
58. Südhof TC. Cerebellin–neurexin complexes instructing synapse properties. *Curr Opin Neurobiol*. 2023;81:102727.
59. Brown LE, Nicholson MW, Arama JE, Mercer A, Thomson AM, Jovanovic JN. γ-Aminobutyric Acid Type A (GABAA) Receptor Subunits Play a Direct Structural Role in Synaptic Contact Formation via Their N-terminal Extracellular Domains*. *Journal of Biological Chemistry*. 2016;291:13926-13942.
60. Fasham J, Huebner AK, Liebmann L, *et al*. SLC4A10 mutation causes a neurological disorder associated with impaired GABAergic transmission. *Brain*. 2023;146:4547-4561.
61. Chiu CS, Jensen K, Sokolova I, *et al*. Number, density, and surface/cytoplasmic distribution of GABA transporters at presynaptic structures of knock-in mice carrying GABA transporter subtype 1-green fluorescent protein fusions. *J Neurosci*. 2002;22:10251-10266.
62. Ouchi N, Oshima Y, Ohashi K, *et al*. Follistatin-like 1, a secreted muscle protein, promotes endothelial cell function and revascularization in ischemic tissue through a nitric-oxide synthase-dependent mechanism. *J Biol Chem*. 2008;283:32802-32811.
63. Li K-C, Zhang F-X, Li C-L, *et al*. Follistatin-like 1 Suppresses Sensory Afferent Transmission by Activating Na+,K+-ATPase. *Neuron*. 2011;69:974-987.
64. Xi Y, Hao M, Liang Q, Li Y, Gong DW, Tian Z. Dynamic resistance exercise increases skeletal muscle-derived FSTL1 inducing cardiac angiogenesis via DIP2A-Smad2/3 in rats following myocardial infarction. *J Sport Health Sci*. 2021;10:594-603.

65. Li M-Y, Gao R-P, Zhu Q, Chen Y, Tao B-B, Zhu Y-C. Skeletal muscle-derived FSTL1 starting up angiogenesis by regulating endothelial junction via activating Src pathway can be upregulated by hydrogen sulfide. *Am J Physiol*. 2023;325:C1252-C1266.
66. Shen Q, Rigor RR, Pivetti CD, Wu MH, Yuan SY. Myosin light chain kinase in microvascular endothelial barrier function. *Cardiovasc Res*. 2010;87:272-280.
67. Rigor RR, Shen Q, Pivetti CD, Wu MH, Yuan SY. Myosin Light Chain Kinase Signaling in Endothelial Barrier Dysfunction. *Medicinal Research Reviews*. 2013;33:911-933.
68. Peyre M, Miyagishima D, Bielle F, *et al*. Somatic PIK3CA Mutations in Sporadic Cerebral Cavernous Malformations. *New England J Med*. 2021;385:996-1004.
69. Tomás A, Pojo M. PIK3CA Mutations: Are They a Relevant Target in Adult Diffuse Gliomas? *Int J Mol Sci*. 2025;26
70. Hong T, Xiao X, Ren J, *et al*. Somatic MAP3K3 and PIK3CA mutations in sporadic cerebral and spinal cord cavernous malformations. *Brain*. 2021;144:2648-2658.
71. Acosta CH, Clemons GA, Citadin CT, *et al*. PRMT7 can prevent neurovascular uncoupling, blood-brain barrier permeability, and mitochondrial dysfunction in repetitive and mild traumatic brain injury. *Exp Neurol*. 2023;366:114445.
72. Salmon M, Schaheen B, Spinosa M, *et al*. ZFP148 (Zinc-Finger Protein 148) Binds Cooperatively With NF-1 (Neurofibromin 1) to Inhibit Smooth Muscle Marker Gene Expression During Abdominal Aortic Aneurysm Formation. *Arterioscler Thromb Vasc Biol*. 2019;39:73-88.
73. Cheng S, Liu L, Wang D, *et al*. Upregulation of the ZNF148/PTX3 axis promotes malignant transformation of dendritic cells in glioma stem-like cells microenvironment. *CNS Neurosci Ther*. 2023;29:2690-2704.
74. Reboll MR, Korf-Klingebiel M, Klede S, *et al*. EMC10 (Endoplasmic Reticulum Membrane Protein Complex Subunit 10) Is a Bone Marrow-Derived Angiogenic Growth Factor Promoting Tissue Repair After Myocardial Infarction. *Circulation*. 2017;136:1809-1823.
75. Apte RS, Chen DS, Ferrara N. VEGF in Signaling and Disease: Beyond Discovery and Development. *Cell*. 2019;176:1248-1264.
76. Shaik F, Cuthbert GA, Homer-Vanniasinkam S, Muench SP, Ponnambalam S, Harrison MA. Structural Basis for Vascular Endothelial Growth Factor Receptor Activation and Implications for Disease Therapy. *Biomolecules*. 2020;10(12).
77. Markovic-Mueller S, Stuttfeld E, Asthana M, *et al*. Structure of the Full-length VEGFR-1 Extracellular Domain in Complex with VEGF-A. *Structure*. 2017;25:341-352.
78. Brozzo MS, Bjelić S, Kisko K, *et al*. Thermodynamic and structural description of allosterically regulated VEGFR-2 dimerization. *Blood*. 2012;119:1781-1788.
